# Efficient automated solid-phase synthesis of recognition-encoded melamine oligomers†

**DOI:** 10.1039/d4sc00973h

**Published:** 2024-03-27

**Authors:** Mohit Dhiman, Rafel Cabot, Christopher A. Hunter

**Affiliations:** a Yusuf Hamied Department of Chemistry, University of Cambridge Lensfield Road Cambridge CB2 1EW UK herchelsmith.orgchem@ch.cam.ac.uk

## Abstract

Recognition-encoded melamine oligomers (REMO) are synthetic polymers with an alternating 1,3,5-triazine-piperazine backbone and side chains equipped with either a phenol or phosphine oxide recognition unit. Here, we describe an automated method for highly efficient solid-phase synthesis (SPS) of REMO of any specified length and sequence. These SPS protocols are amongst the most robust reported to date, as demonstrated by the synthesis of a mixed-sequence 42-mer, which was obtained in excellent crude purity on a 100 mg scale. Starting from loaded Wang resin and dichlorotriazine monomer building blocks, the SPS methods were automated and optimised on a commercial peptide synthesiser. Major side products were identified using LCMS, and the undesired side reactions were suppressed by the choice of resin, solvent and coupling conditions. REMO have been shown to form high-fidelity length- and sequence-selective H-bonded duplexes, analogous to nucleic acids, and automated synthesis will facilitate exploration of related functional properties, such as molecular replication and programmable self-assembly.

## Abstract 2

An automated solid-phase synthesis route has been developed to rapidly synthesise recognition-encoded melamine oligomer sequences up to 42 monomer units long in remarkably high yield and excellent crude purity.

## Graphical abstract

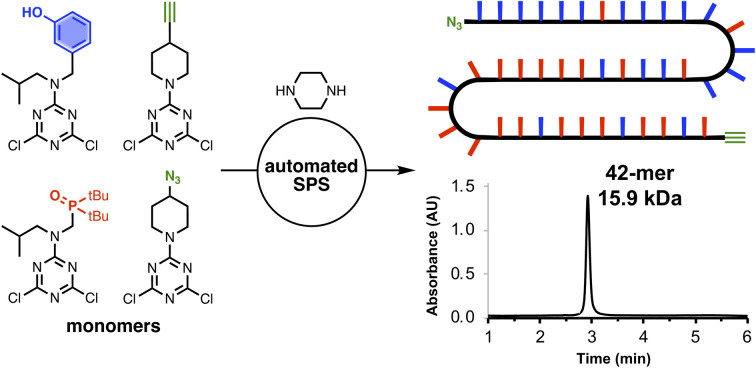

## Introduction

**Fig. 1:**
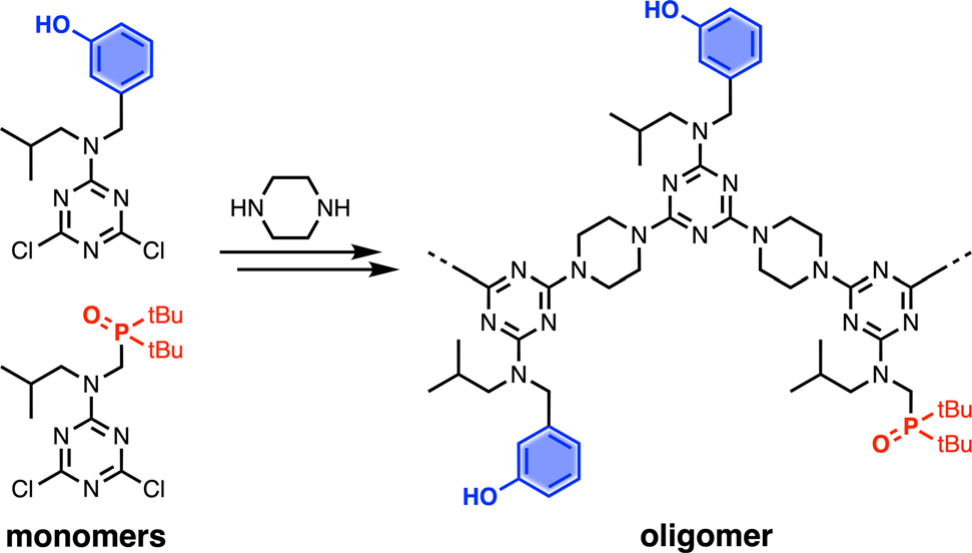
Recognition-Encoded Melamine Oligomers (REMO) synthesised from piperazine and dichlorotriazines equipped with complementary recognition units.

Recognition-Encoded Melamine Oligomers (REMO) are a new class of synthetic information molecule which form length- and sequence-selective H-bonded duplexes with remarkably high fidelity.^1^ As shown in Fig. 1, the oligomer backbone consists of alternating piperazine and triazine units, and the side chains carry the phenol or phosphine oxide recognition units that are responsible for base-pairing interactions between complementary sequences. Solution-phase synthesis has been used to make REMO up to four monomer units long, taking advantage of the high-yielding nucleophilic aromatic substitution (S_N_Ar) reactions of chlorotriazines.^1^ Starting from cyanuric chloride, sequential substitution of each chlorine with the nitrogen of a secondary amine reduces the reactivity.^2–4^ At low temperatures, only the first chlorine is substituted, which provides access to the dichlorotriazine building blocks shown in Fig. 1. Substitution of the second chlorine with piperazine occurs at room temperature, and substitution of the third chlorine to give an oligomer requires elevated temperatures. However, as the length of the REMO increases, the number of synthetic and purification steps rapidly becomes laborious. In addition, the number of different sequences that are possible increases exponentially with chain length. In order to explore this potentially rich chemical space, the development of a synthesis methodology that allows facile access to long oligomers with total sequence control is necessary.

Solid-phase synthesis (SPS) is well established as a method for efficiently making sequence-defined polymers. High yielding amide and phosphoramidite coupling reactions were originally developed for SPS of peptides^5^ and oligonucleotides,^6^ and this chemistry, as well as other coupling reactions, have been adapted for the synthesis of non-natural sequence-defined oligomers.^7–17^ The efficiency of the S_N_Ar coupling chemistry used for REMO synthesis makes these oligomers an attractive target for the development of an automated SPS method. When carried out under microwave conditions, S_N_Ar reactions of chlorotriazines are fast and quantitative and require no reagents other than a base to neutralise the hydrochloric acid by-product.^1^ In addition, the fact that triazines ionise well in ESI-MS provides a powerful tool for the detection of side products and optimisation of reaction conditions using LCMS.

There are a number of parameters that determine the success of an SPS route. Clearly the coupling yields must be very high (*i.e.* >99%), and suppressing side reactions will make the final purification easier. A frequently encountered issue in peptide SPS is folding or aggregation on resin, which can hinder accessibility of reactive sites for coupling steps.^18^ Reaction time, temperature, solvent, and resin are all variables that can be optimised to maximise the efficiency of the protocol. Here we describe a two-step iterative SPS route for REMO that has been automated using a CEM Liberty Blue Peptide Synthesiser. Side products were identified using LCMS and eliminated by changing the reaction conditions to obtain an extremely efficient protocol, which was used to synthesise REMO up to 42 monomer units long in remarkably high yield and purity.

## Results and discussion

### Building block synthesis

**Scheme 1:**
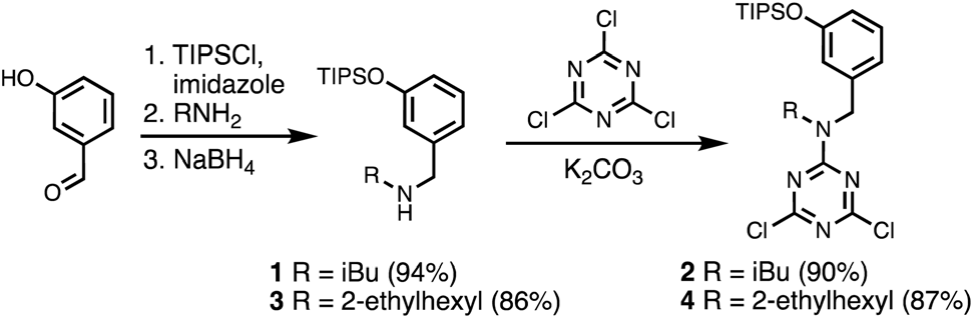
Synthesis of dichlorotriazine building blocks 2 and 4.

A set of dichlorotriazine building blocks were first synthesised for use in the SPS routes described below. These building blocks must be obtained in large amounts, because high concentrations of fresh reagents are needed for each SPS coupling cycle. Two different H-bond donor building blocks bearing either isobutyl or 2-ethylhexyl solubilising groups were synthesised on a multi-decagram scale (Scheme 1). 3-Hydroxybenzaldehyde was protected using triisopropylsilyl chloride followed by reductive amination with either isobutylamine or 2-ethylhexylamine to give amines 1 and 3 in very good yield. Reaction of 1 and 3 with cyanuric chloride at −10 °C yielded the dichlorotriazine building blocks 2 and 4, respectively.

**Scheme 2:**
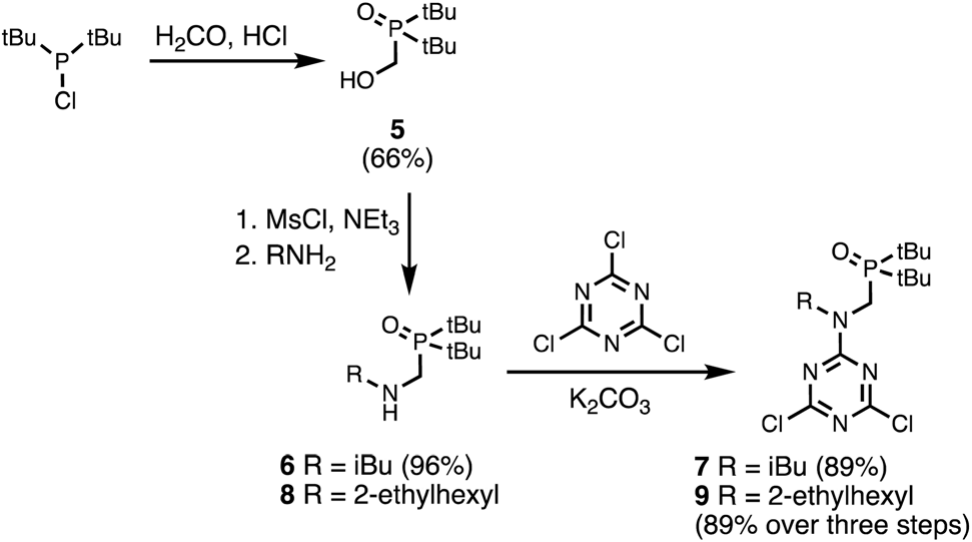
Synthesis of dichlorotriazine building blocks 7 and 9.

Two different H-bond acceptor building blocks bearing either isobutyl or 2-ethylhexyl solubilising groups were also synthesised on a multi-decagram scale (Scheme 2). Di-*tert*-butylchlorophosphine was heated with formaldehyde in aqueous acid to yield phosphine oxide 5. Mesylation of the hydroxyl group followed by microwave reaction with either isobutylamine or 2-ethylhexylamine gave amines 6 and 8. Reaction of 6 and 8 with cyanuric chloride at −10 °C yielded the dichlorotriazine building blocks 7 and 9, respectively.

**Scheme 3:**
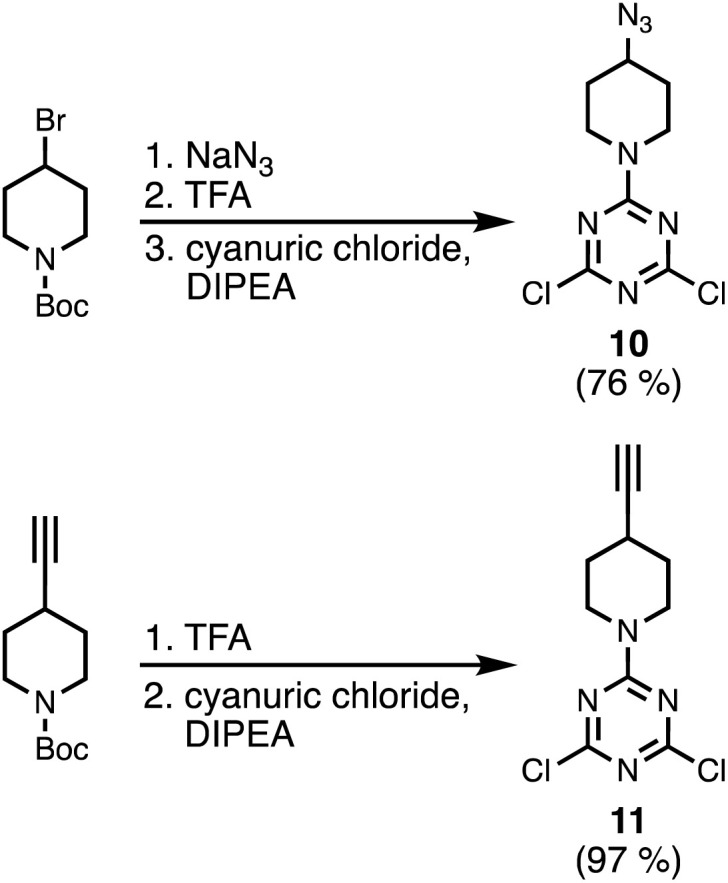
Synthesis of azide and alkyne dichlorotriazine building blocks.

Scheme 3 shows the synthesis of an azide-functionalised dichlorotriazine 10 from 1-Boc-4-bromopiperidine. Nucleophilic substitution with sodium azide, followed by deprotection of the Boc group with TFA gave the piperidine. Subsequent reaction with cyanuric chloride at −78 °C yielded 10. The alkyne-functionalised dichlorotriazine 11 was similarly synthesised by deprotection of 1-Boc-4-ethylnylpiperidine with TFA, followed by reaction with cyanuric chloride at −78 °C (Scheme 3).

### Functionalisation of the resin

**Scheme 4:**
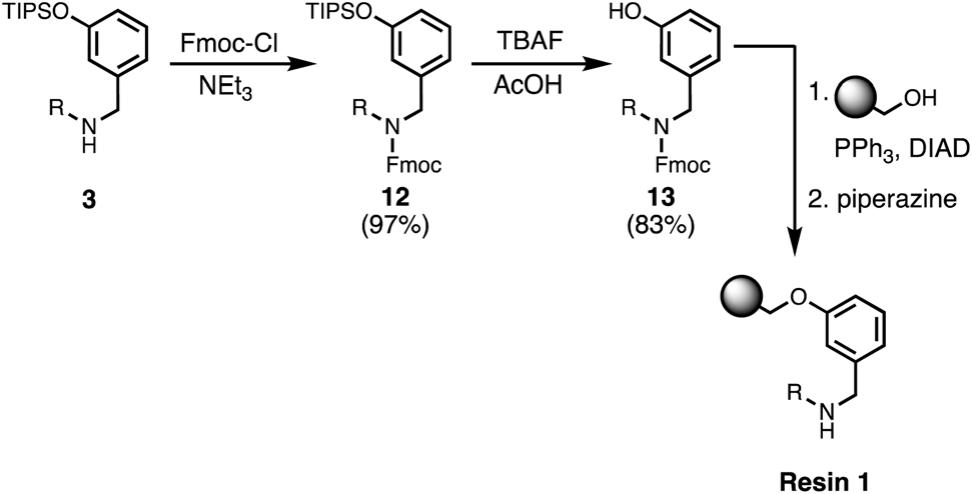
Functionalisation of Wang resin. R = 2-ethylhexyl.

Wang resin was chosen for SPS, since it provided a method for attachment of a phenol recognition unit *via* a base-stable linkage. The ether bond can withstand high microwave temperatures for many coupling cycles, and acidic cleavage is facile. Scheme 4 shows the route used for functionalisation of Wang resin. Amine 3 was Fmoc protected in excellent yield to give 12. Subsequent TBAF deprotection of the silyl protecting group was carried out under mildly acidic conditions, which was essential to prevent Fmoc deprotection. Wang resin was then reacted with 13 under Mitsunobu conditions followed by Fmoc deprotection with piperazine to give Resin 1. Treatment of a small amount of the Fmoc-protected resin with DBU allowed the loading to be determined (0.36 mmol g^−1^).

### Automated solid-phase synthesis

**Scheme 5:**
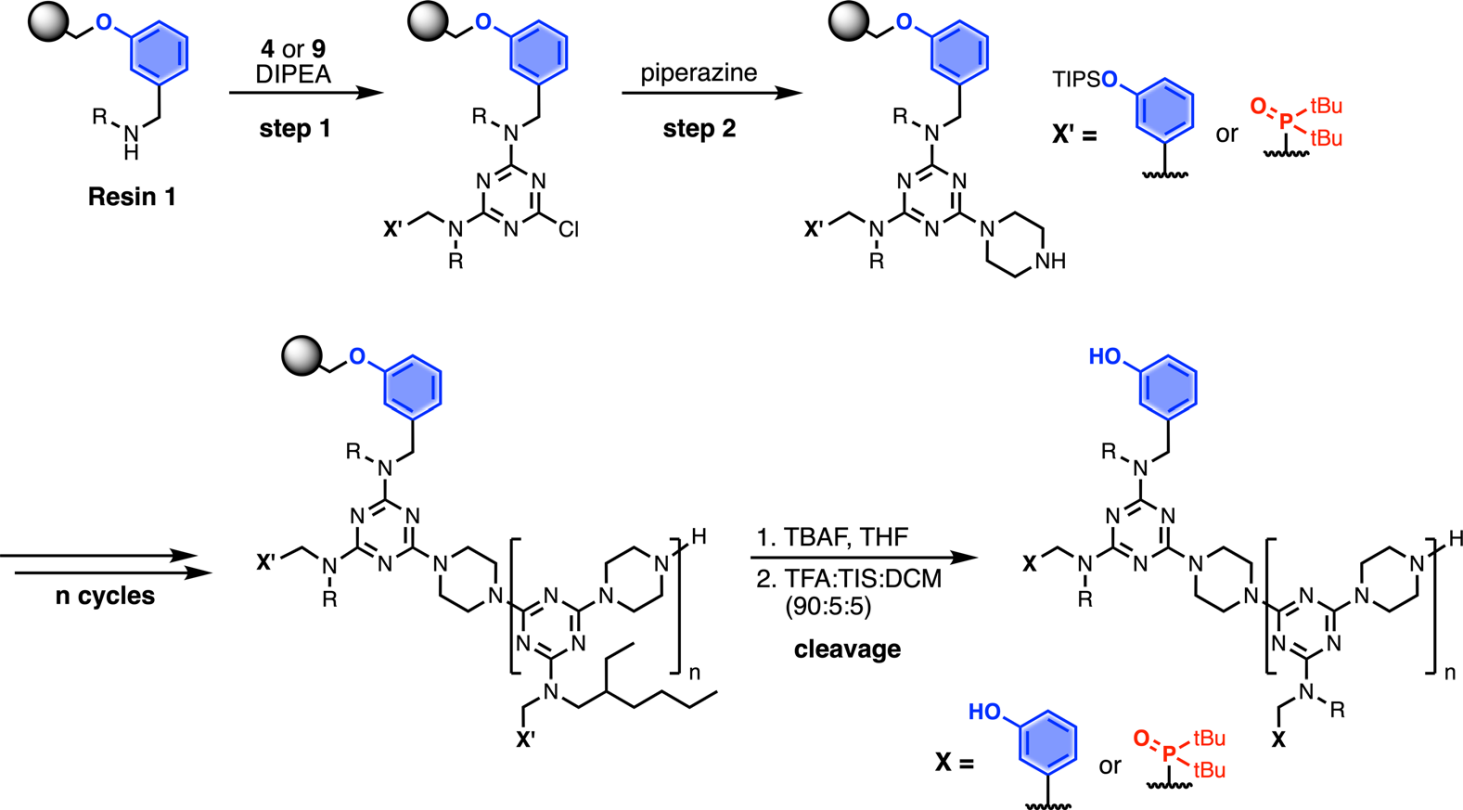
Automated SPS protocol for REMO synthesis *via* a two-step coupling cycle. Coupling reactions were carried out in DMF under microwave conditions. R = 2-ethylhexyl.

Automated SPS was carried out on a CEM Liberty Blue Peptide Synthesiser using custom reagents and microwave methods. For the method development, building blocks with 2-ethylhexyl groups were utilised to increase the solubility of the oligomeric products. Scheme 5 shows the route employed for method development. Resin 1 was reacted with either phenol dichlorotriazine 4 or phosphine oxide dichlorotriazine 9 in step 1, and then piperazine in step 2. After the desired number of coupling cycles, the phenol groups were deprotected on-resin by treatment with TBAF, followed by resin cleavage under acidic conditions. The initial tests described below identified significant side reactions, which enabled changes to the reaction conditions to optimise the protocol.

### Resin cross-reaction

**Fig. 2:**
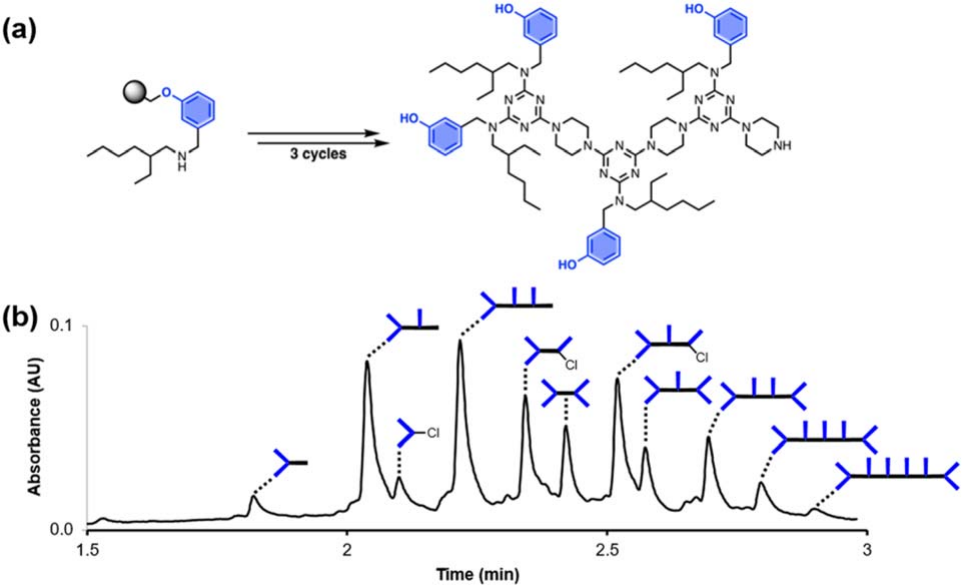
(a) Automated SPS of D^2^DD using the route shown in Scheme 5. Step 1 conditions: 0.1 M dichlorotriazine at 50 °C for 10 min. Step 2 conditions: 0.1 M piperazine at 50 °C for 10 min. (b) UPLC trace of the crude product. Cartoon representations of the products identified using ESI-MS are shown. UPLC Conditions: C4 column at 40 °C using a 5–100% gradient of MeCN/formic acid (0.1%) in water/formic acid (0.1%) over 2 minutes, then 100% MeCN/formic acid (0.1%) over 1 minute.

Fig. 2 shows the attempted synthesis of oligomer D^2^DD on Resin 1*via* three coupling cycles with the phenol dichlorotriazine building block (D^2^ represents a triazine functionalised with two donor recognition units). The UPLC trace of the crude product obtained after cleavage from the resin shows that many different oligomers are present (Fig. 2b). The target compound D^2^DD is formed (retention time of 2.22 min), but 10 different side products could be identified using ESI-MS. Five of the side products come from incomplete coupling in either step 1 or step 2 of the coupling cycle, giving rise to oligomers with recognition unit deletions or a terminal monochlorotriazine. This result suggests that higher temperatures and/or longer reaction times should be used, and increasing the microwave reaction temperature from 50 °C to 90 °C was found to significantly reduce these side products.

**Fig. 3:**
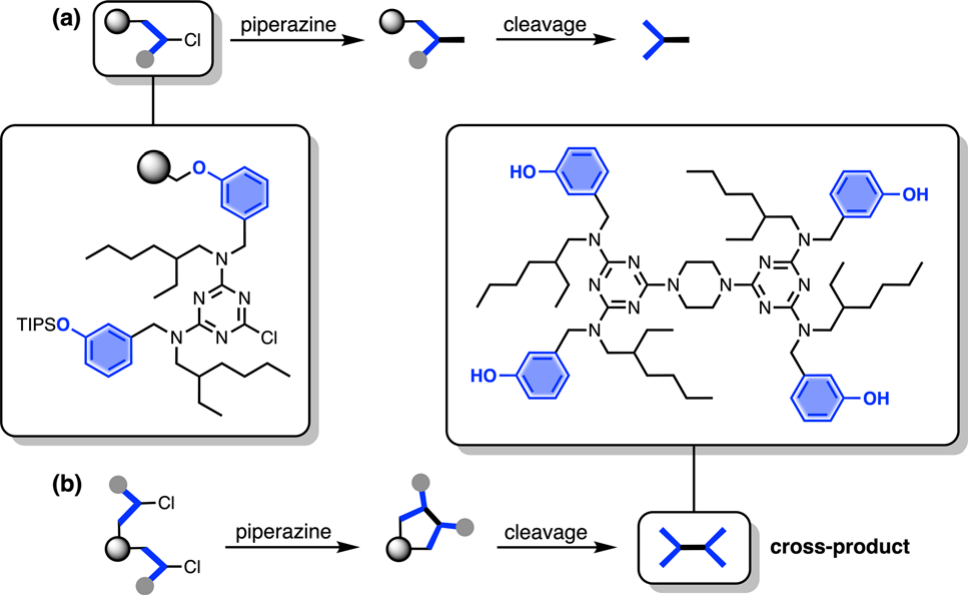
(a) Reaction pathway where each monochlorotriazine on the resin reacts with a molecule of piperazine from solution, leading to the product that propagates chain growth. (b) Reaction pathway where one equivalent of piperazine reacts with two monochlorotriazines on the same resin bead leading to the cross-product, which terminates chain growth.

The other five side products in Fig. 2b have D^2^ motifs at both ends of the oligomer. These compounds arise from coupling of two different oligomer chains on the same resin bead, as outlined in Fig. 3. During step 2 of the SPS coupling cycle, each growing oligomer chain should react with one equivalent of piperazine (Fig. 3a). However, if there are two oligomers in close proximity on a resin bead, then reaction of both of the monochlorotriazine end groups with the same molecule of piperazine will lead to cross-products (Fig. 3b). There are cross-products containing both odd and even numbers of recognition units due to the incomplete coupling. It should be possible to suppress the side products due to resin cross-reaction by increasing the concentration of piperazine in step 2 of the coupling cycle, and some success was achieved by using 0.7 M instead of 0.1 M piperazine.

### Dimethylamino capping

**Fig. 4:**
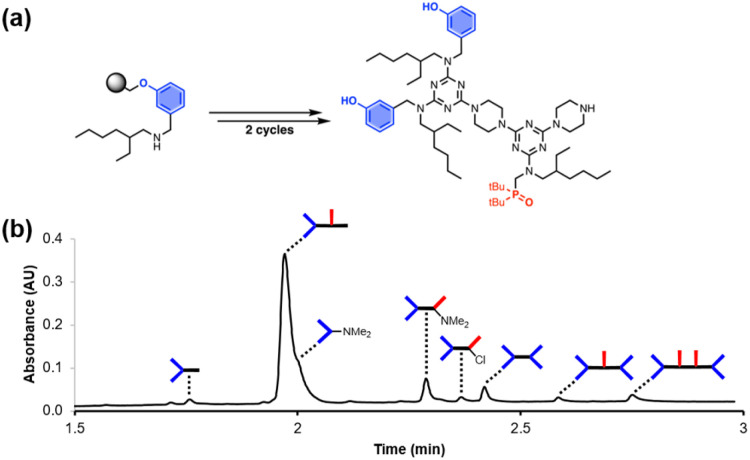
(a) Automated SPS of D^2^A using the route shown in Scheme 5. Step 1 conditions: 0.1 M dichlorotriazine at 90 °C for 10 min. Step 2 conditions: 0.7 M piperazine at 90 °C for 10 min. (b) UPLC trace of the crude product. Cartoon representations of the products identified using ESI-MS are shown. UPLC Conditions: C4 column at 40 °C using a 5–100% gradient of MeCN/formic acid (0.1%) in water/formic acid (0.1%) over 2 minutes, then 100% MeCN/formic acid (0.1%) over 1 minute.

These improved reaction conditions were then used to synthesise oligomer D^2^A on Resin 1*via* one coupling cycle with the phenol dichlorotriazine building block, followed by one coupling cycle with the phosphine oxide dichlorotriazine building block (Fig. 4). The UPLC trace of the crude product obtained after cleavage from the resin shows that the target compound is now the major product (Fig. 4b). Side products due to incomplete coupling have been largely eliminated, and although cross-products with D^2^ at both ends of the oligomer chain are still present, they have been significantly reduced compared with Fig. 2b. However, two side products due to reaction of a monochlorotriazine chain end with dimethylamine were identified. Dimethylamine is present as an impurity in the solvent (DMF) and appears to be present in sufficient quantities to compete with piperazine in step 2 of the coupling cycle. It proved possible to eliminate this side reaction by using high-purity peptide grade DMF.

### Optimised SPS protocol

**Scheme 6:**
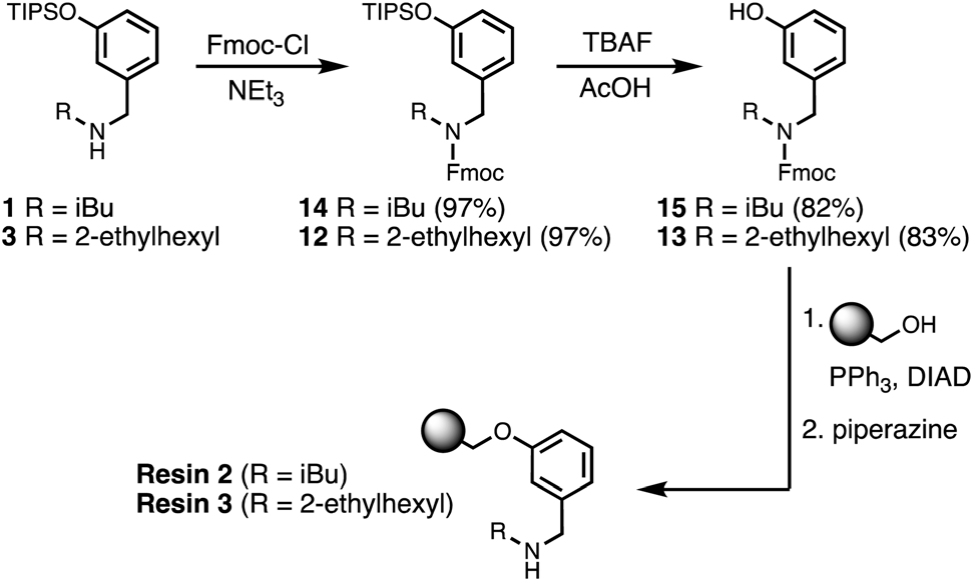
Functionalisation of TentaGel Wang resin with either R = isobutyl (loading = 0.14 mmol g^−1^) or R = 2-ethylhexyl (loading = 0.10 mmol g^−1^).

One further significant change was implemented for the final automated SPS route. Wang resin was replaced with TentaGel Wang resin, a low-loading resin with a PEG linker between the resin matrix and the 4-hydroxybenzyl group. This resin increases the distance between growing oligomer chains and completely eliminated all side products due to resin cross-reaction. Scheme 6 shows the route used for functionalisation of TentaGel resin with phenol units bearing two different solubilising groups.

**Scheme 7:**
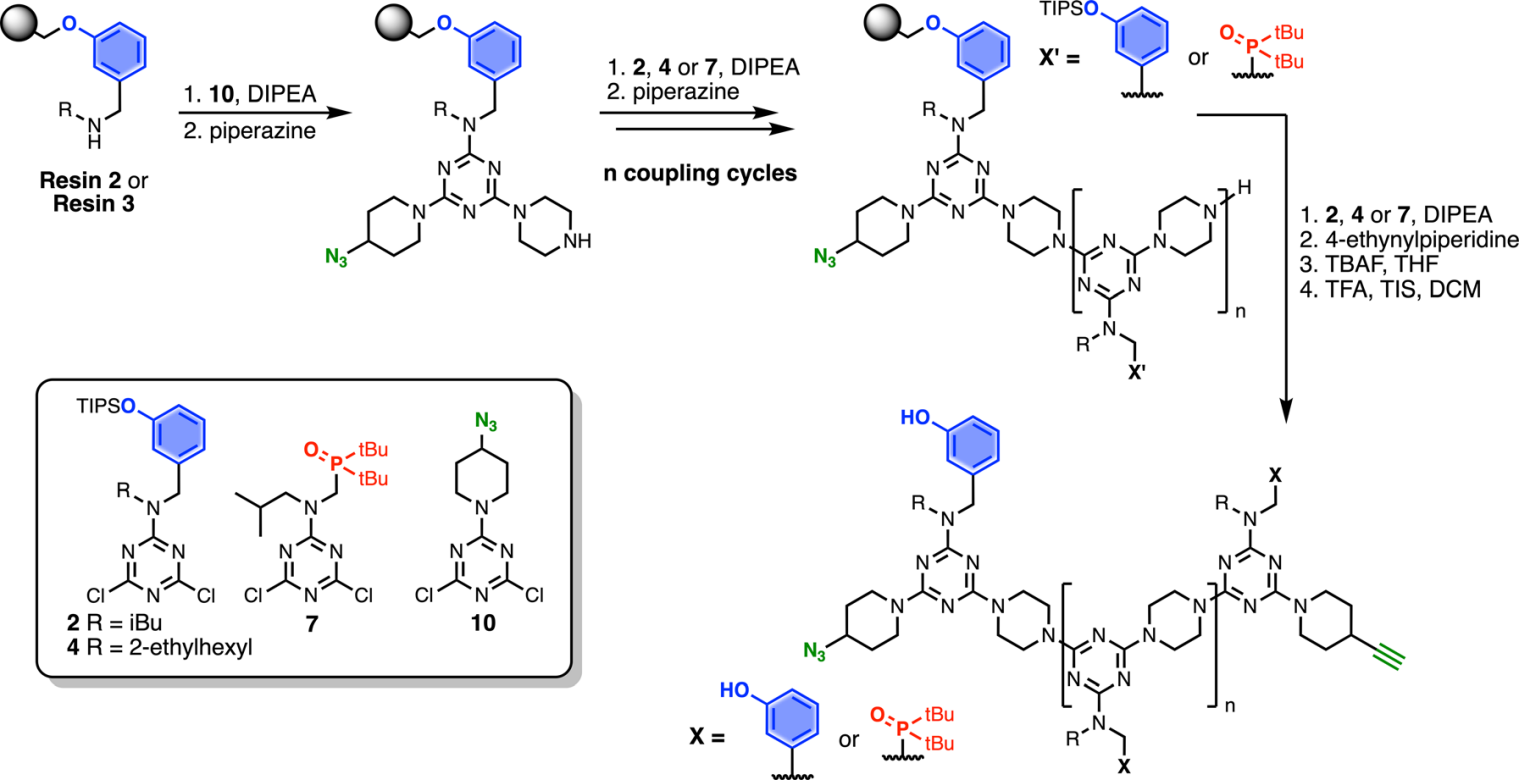
Route for automated SPS of REMO bearing terminal azide and alkyne groups. Coupling reactions were carried out in DMF under microwave conditions at 90 °C for 10 minutes (or 15 minutes for 7) using 0.1 M dichlorotriazine or 0.7 M piperazine. R = isobutyl or 2-ethylhexyl.

Scheme 7 shows the optimised, automated SPS route to REMO bearing terminal alkyne and azide groups. Functionalised TentaGel Wang resin was alternatingly reacted with a dichlorotriazine, then piperazine, under microwave conditions. The first coupling cycle utilised 10 to introduce a terminal azide group at one end of the oligomer. Subsequent coupling cycles with 2, 4 or 7 introduced the sequence of recognition units into the oligomer. In the final coupling step, the oligomer was capped with 4-ethynylpiperidine to introduce a terminal alkyne. Removal of silyl protecting groups was carried out on-resin before cleavage of the oligomer from the resin with TFA.

**Fig. 5:**
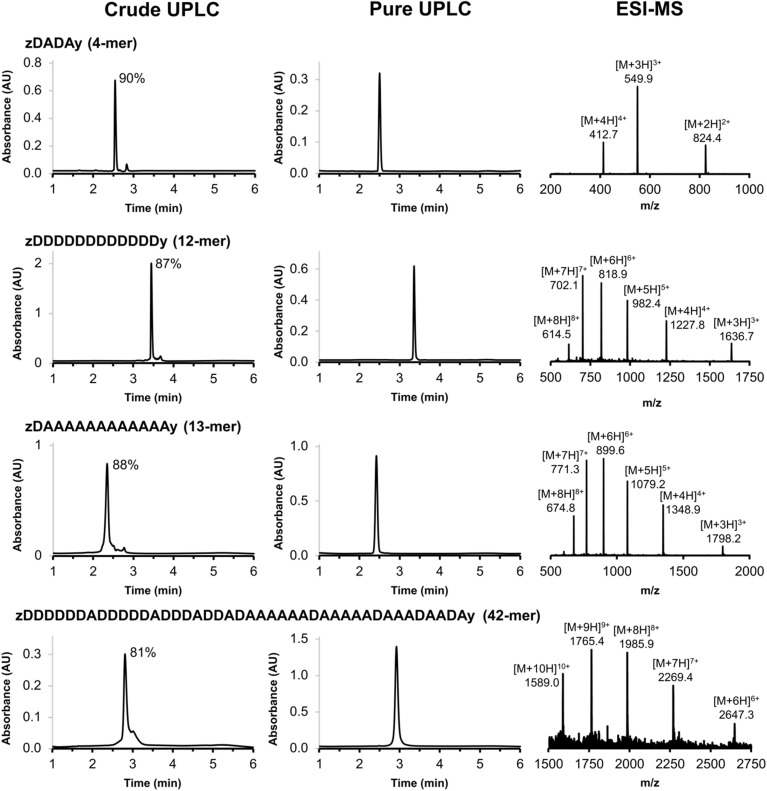
UPLC traces of the crude and purified products obtained using the automated SPS route shown in Scheme 7 for a 4-mer, 12-mer, 13-mer and 42-mer REMO. The sequences of the oligomers are described using upper case letters for the recognition units (D for phenol and A for phosphine oxide) and lower case letters for the end groups (z for azide, and y for alkyne). The crude purities are shown next to the major UPLC peaks, and the ESI-MS of the purified products are shown (see ESI† for calculated ESI^+^ masses). UPLC Conditions: C4 column at 40 °C using a 30–100% gradient of THF/formic acid (0.1%) in water/formic acid (0.1%) over 4 minutes, then 100% THF/formic acid (0.1%) over 2 minutes.

The scope of the automated SPS method was investigated by synthesising REMO of increasing length. Fig. 5 illustrates four REMO sequences, which were all synthesised on a 50 μmol scale. The crude UPLC traces show exceptionally high crude purity, at 90% for a 4-mer, 88% for a 13-mer, and 81% for a 42-mer. None of the impurities present corresponded to incomplete coupling, or any of the side products discussed above, even after 84 sequential S_N_Ar reactions on the resin, showing the robustness of the methodology. Preparative HPLC was used to isolate all four oligomers with greater than 99% purity (Fig. 5) and with isolated yields of 22–49% based on the initial resin loading (see ESI†). The ESI-MS mass envelopes for each oligomer were used in conjunction with HRMS, ^1^H and ^31^P NMR spectroscopy to characterise the products (see ESI† for details).

**Fig. 6:**
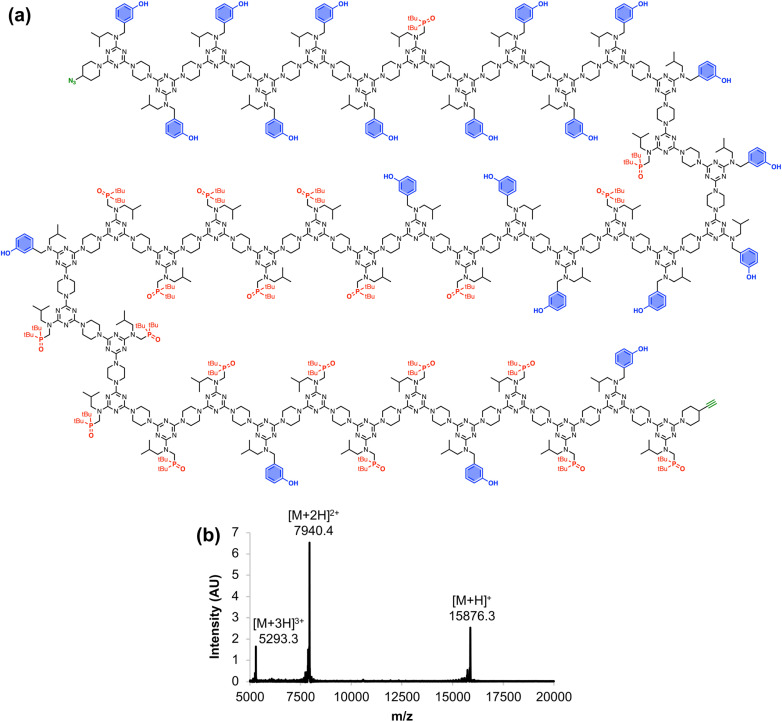
(a) Chemical structure and (b) MALDI-TOF mass spectrum of the 42-mer REMO, zDDDDDDADDDDDADDDADDADAAAAAADAAAAADAAADAADAy. Calculated masses: 15876.2 [M+H]^+^, 7940.1 [M+2H]^2+^, 5293.4 [M+3H]^3+^.

Fig. 6 shows the full chemical structure of the 42-mer REMO, along with the MALDI-TOF mass spectrum recorded using a DCTB matrix. Peaks attributable to the [M+H]^+^, [M+2H]^2+^, and [M+3H]^3+^ species can be clearly identified. This compound represents one of the longest non-natural, sequence-defined oligomers to be reported, and with a mass of almost 16 kDa, it is equivalent in size to a 130 amino acid protein.

## Conclusions

An automated solid-phase synthesis (SPS) route has been developed to rapidly synthesise recognition-encoded melamine oligomers (REMO) up to length 42 in excellent crude purity and sequence control. A two-step SPS protocol was automated on a commercial peptide synthesiser, using alternating S_N_Ar reactions with a dichlorotriazine building block and piperazine. The resin, reagents, concentrations and microwave conditions were optimised to obtain fast and quantitative coupling reactions. Two major side reactions were discovered by LCMS analysis and then eliminated by optimisation of the SPS parameters: cross-reaction of two growing oligomer chains on a resin bead was eliminated by using a low-loading TentaGel resin and high reagent concentrations; side reactions with dimethylamine impurities in the solvent were eliminated by using high-purity DMF. The scope of the automated SPS method was demonstrated by the synthesis of a series of mixed-sequence oligomers up to 42 building blocks long. There was no drop in the efficiency of the coupling reactions for longer oligomers. Crude purities of 81–90% make this method one of the most robust SPS protocols for sequence-defined oligomer synthesis reported to date. HPLC purification was used to isolate the final products with greater than 99% purity.

REMO have already been shown to form sequence-selective duplexes in a similar fashion to nucleic acids, and the ability to rapidly access REMO of any desired length and sequence will allow exploration of whether this new class of synthetic information molecule might show some of the other properties of nucleic acids, such as molecular replication or programmable self-assembly. The REMO sequences described in this work include a self-complementary 4-mer that could form a duplex with four base-pairs, and mutually complementary 12- and 13-mers that could form a duplex with twelve base-pairs and an overhanging unpaired base. Equipping these REMO with terminal azide and alkyne groups also opens opportunities for incorporation of new functionality through copper-catalysed alkyne–azide cycloaddition reactions, chain extension by ligation, and covalent trapping of supramolecular assemblies. The SPS methods reported here therefore pave the way for the development of a range of new tools for exploring the supramolecular properties of programmable synthetic macromolecules.

## Data availability

All supporting data is provided in the ESI.†

## Author contributions

The manuscript was written through contributions of all authors.

## Conflicts of interest

There are no conflicts to declare.

## Supplementary Material

SC-015-D4SC00973H-s001
