# Supplementary material for: Efficient automated solid-phase synthesis of recognition-encoded melamine oligomers
Source: Chem Sci. 2024 Mar 27;15(16):5957–63. doi: 10.1039/d4sc00973h (PMC11040639; doi:10.1039/d4sc00973h)
Supplement: SC-015-D4SC00973H-s001 [file SC-015-D4SC00973H-s001.pdf]

Electronic Supplementary Material (ESI) for Chemical Science.
This journal is © The Royal Society of Chemistry 2024

Efficient Automated Solid-Phase Synthesis of Recognition-Encoded Melamine Oligomers

Mohit Dhiman,[1] Rafel Cabot,[1] Christopher A. Hunter [1,*]

[1]Yusuf Hamied Department of Chemistry, University of Cambridge, Cambridge CB2 1EW, U.K. Email: herchelsmith.orgchem@ch.cam.ac.uk

Supplementary Information

1. **General Experimental Details** ***S2***
2. **Synthesis of Building Blocks** ***S3***
3. **Functionalisation of Wang Resin** ***S35***
4. **Protocols for Optimised Automated Solid-Phase Synthesis** ***S36***
5. **Synthesis of Oligomers** ***S38***

1. General Experimental Details

All reagents and materials used in the syntheses described were bought from commercial sources and used without prior purification. Dry solvents were obtained from a Grubbs PS-MD-5 solvent purification system and used with no further degassing. Thin layer chromatography (TLC) was carried out using silica gel 60F (Merck) on glass plates. LCMS analyses of samples were performed using a Waters Acquity H-class UPLC coupled with a single quadrupole Waters SQD2. Two different UPLC columns were used: an Acquity UPLC CSH C18 Column (130 Å, 1.7 µm, 2.1 mm x 50 mm), and an Acquity UPLC PRM PR BEH C4 Column (300 Å, 1.7 µm, 2.1 mm x 50 mm).

Purification of compounds by silica column chromatography were performed using an automated system (Combiflash® Rf+ or Combiflash® Rf+ Lumen) with pre-packaged silica cartridges (25 µm or 50 µm PuriFlash® columns). All NMR spectra were recorded using a Bruker 500 MHz Avance III Smart Probe Spectrometer, a Bruker 400 MHz Avance III HD Spectrometer, a Bruker 400 MHz Avance III HD Smart Probe Spectrometer, or a Bruker 400 MHz Neo Prodigy Spectrometer at 298 ± 0.1 K. The residual ^{1}H form of the solvent was used as the internal standard for referencing. In $CDCl_3$, the ^{1}H spectra were referenced to δ 7.26 ppm and ^{13}C spectra referenced to δ 77.16 ppm. In DMSO-d_6, the ^{1}H spectra were referenced to δ 2.50 ppm and ^{13}C spectra referenced to δ 39.52 ppm. Chemical shifts (δ) are quoted in ppm and coupling constants (*J*) quoted in Hz. Splitting patterns are reported as: s (singlet), bs (broad singlet), d (doublet), t (triplet), q (quartet) and m (multiplet). FT-IR spectra were collected with an ALPHA FT-IR Spectrometer from Bruker. HRMS spectra were recorded using a Waters SQD2 with Waters H-Class UPLC, equipped with a Waters Acquity UPLC BEH C18 Column (130 Å, 1.7µm, 2.1 mm x 50 mm).

Analytical reverse-phase HPLC was performed on an Agilent HP-1100 Series HPLC system. Preparative reverse-phase HPLC was performed on an Agilent HP-1100 Series preparative HPLC system.

UV-vis spectra were collected on an Agilent Cary 60 UV-vis spectrophotometer controlled by Cary WinUV software.

2. Synthesis of Building Blocks

Synthesis of 1

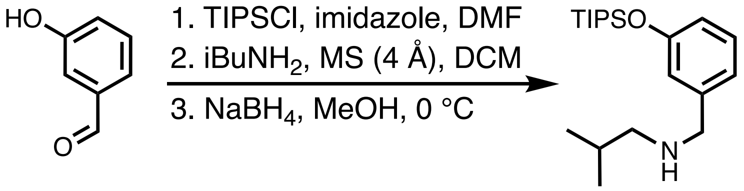

To a solution of 3-hydroxybenzaldehyde (11.1 g, 90.9 mmol) in dry DMF (100 mL) under N_2 at 0 °C was added imidazole (13.6 g, 199.8 mmol), then the mixture was stirred until a clear solution formed. Triisopropylsilyl chloride (21.5 mL, 19.3 g, 99.9 mmol) was added dropwise then the solution was stirred for 2 h at r.t. before it was quenched with water (100 mL). The aqueous layer was extracted with EtOAc (3x) before the combined organic layers were washed with water (3x), 5% LiCl soln. (3x) and brine. The organic phase was dried over $MgSO_4$ and then the solvent was removed *in vacuo* to yield a pale-yellow oil (28.8 g).

Isobutylamine (9.94 mL, 7.31 g, 99.9 mmol) and molecular sieves (4 Å) were added to a solution of the crude product from the previous step (28.8 g) in DCM (150 mL) at r.t. The solution was stirred at r.t. until complete conversion of the aldehyde, which was monitored by ^{1}H NMR. The molecular sieves were removed, and the solvent was evaporated *in vacuo* to obtain a colourless oil.

MeOH (200 mL) was added, and the solution was cooled down to 0 ºC before sodium borohydride (3.78 g, 99.9 mmol) was added. The reaction mixture was stirred at r.t. until the disappearance of the imine intermediate, as monitored by ^{1}H NMR. The solvent was removed *in vacuo* and the residue was dissolved in 1 M NaOH solution (500 mL). The solution was extracted with DCM (3x). The organic phase was dried over $MgSO_4$ and then the solvent was removed *in vacuo* to yield the crude product. The crude was purified by flash chromatography (SiO_2, 0-10% gradient of MeOH in DCM) to yield the pure product **1** as a pale-yellow oil (28.82 g, 85.8 mmol, 94% over three steps).

^{1}H NMR (400 MHz, chloroform-d): δ_H 7.15 (t, *J* = 7.8 Hz, 1H), 6.89-6.84 (m, 2H), 6.75 (ddd, *J* = 7.8, 2.5, 1.2 Hz, 1H), 3.73 (s, 2H), 2.40 (d, *J* = 6.8 Hz, 2H), 1.76 (non, *J* = 6.8 Hz, 1H), 1.32 (br s, 1H), 1.25 (hept, *J* = 7.1 Hz, 3H), 1.10 (d, *J* = 7.1 Hz, 18H), 0.90 (d, *J* = 6.7 Hz, 6H).

The spectroscopic data matches that previously reported in the literature at:

Troselj, P., Bolgar, P., Ballester, P., Hunter, C.A.

High Fidelity Sequence-Selective Duplex Formation by Recognition-Encoded Melamine Oligomers. *J. Am. Chem. Soc.* **2021**, *143,* 8669-8678.

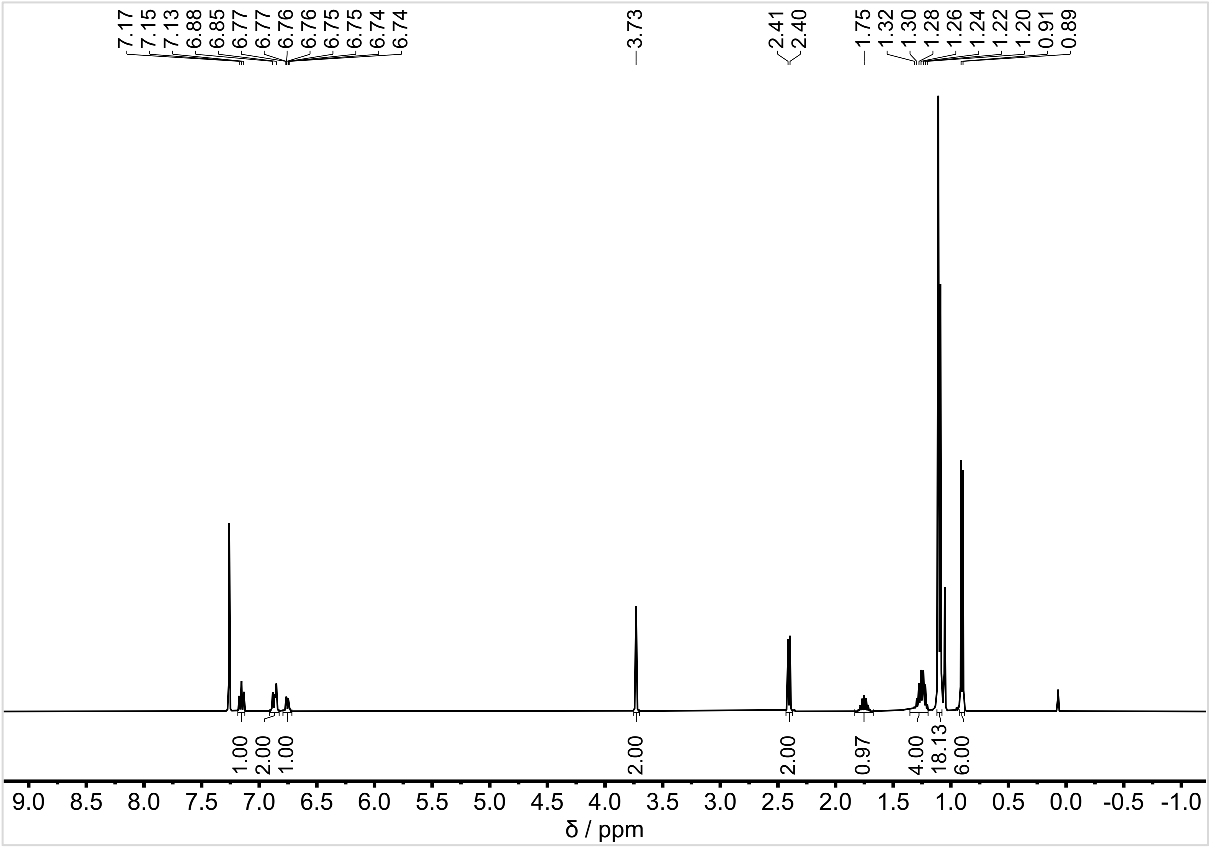

Figure S1: ^{1}H NMR (400 MHz, chloroform-d) spectrum of **1**.

Synthesis of 2

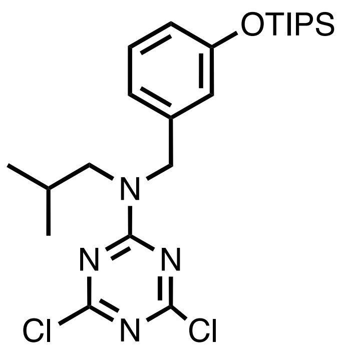

To a solution of cyanuric chloride (17.4 g, 94.4 mmol) and K_2CO_3 (23.7 g, 171.6 mmol) in THF (220 mL) at -10 °C was added dropwise a solution of **1** (28.82 g, 85.8 mmol) in THF (80 mL). The solution was stirred at -10 °C for 1 h before the solvent was removed *in vacuo* and the residue was dissolved in EtOAc. The solution was washed with 0.1 M HCl (3x) and brine before the organic phase was dried over $MgSO_4$, then the solvent was removed *in vacuo* to yield the crude product. The crude was purified by flash chromatography (SiO_2, 0-20% gradient of EtOAc in 40-60 petroleum ether) to yield the pure product **2** as a pale-yellow oil (37.31 g, 77.2 mmol, 90%).

^{1}H NMR (400 MHz, chloroform-d): δ_H 7.18 (t, *J* = 7.8 Hz, 1H), 6.85-6.75 (m, 2H), 6.71 (t, *J* = 2.0 Hz, 1H), 4.80 (s, 2H), 3.39 (d, *J* = 7.6 Hz, 2H), 2.12 (m, 1H), 1.19 (m, 3H), 1.07 (d, *J* = 7.2 Hz, 18H), 0.92 (d, *J* = 6.7 Hz, 6H).

The spectroscopic data matches that previously reported in the literature at:
Troselj, P., Bolgar, P., Ballester, P., Hunter, C.A.
High Fidelity Sequence-Selective Duplex Formation by Recognition-Encoded Melamine Oligomers. *J. Am. Chem. Soc.* **2021**, *143,* 8669–8678.

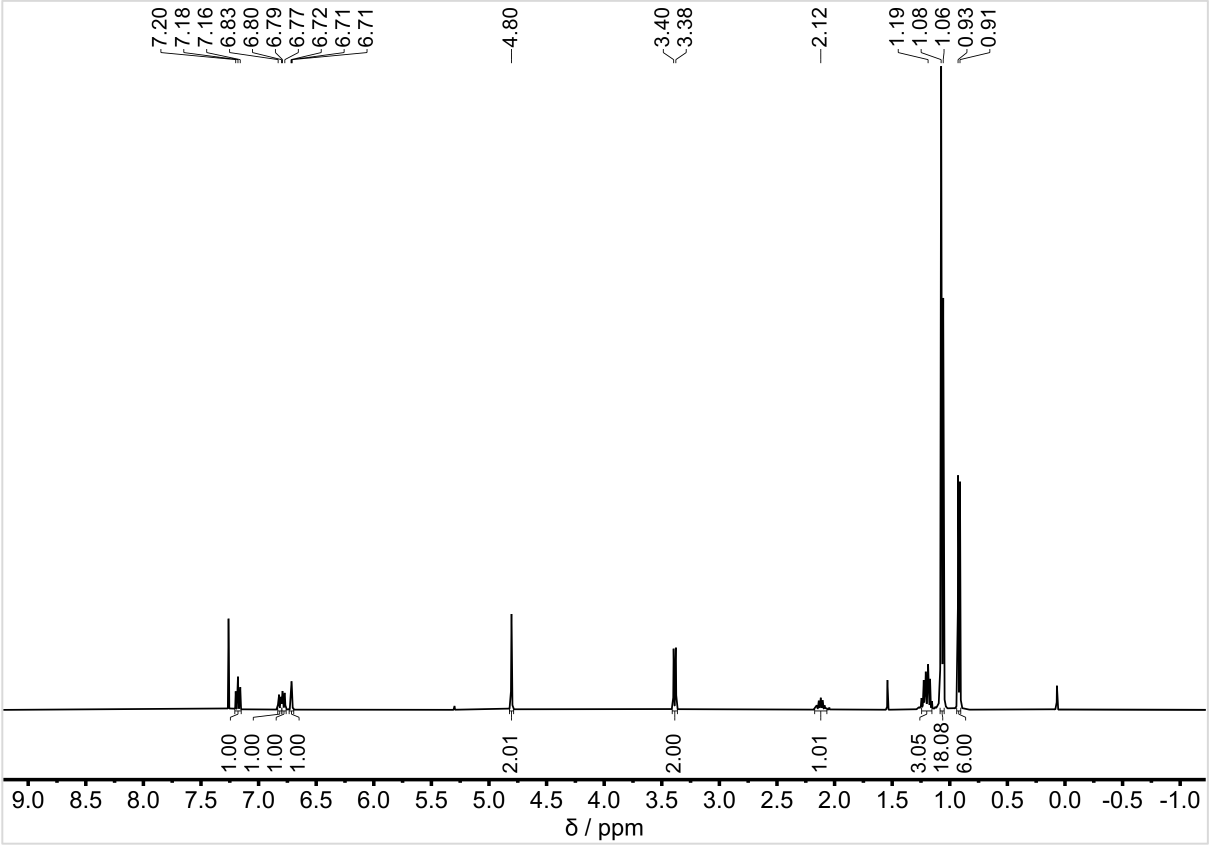

Figure S2: ^{1}H NMR (400 MHz, chloroform-d) spectrum of **2**.

Synthesis of 3

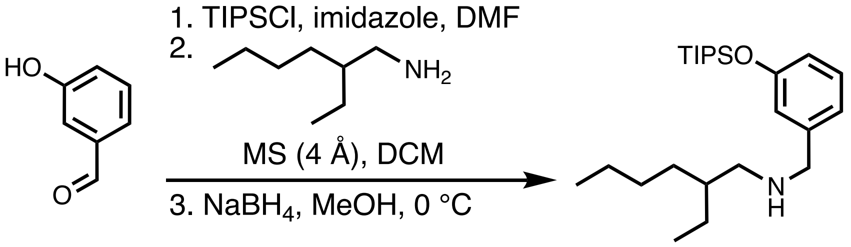

To a solution of 3-hydroxybenzaldehyde (9.0 g, 73.7 mmol) in dry DMF (50 mL) under N_2 at 0 °C was added imidazole (11.0 g, 162.1 mmol), then the mixture was stirred until a clear solution formed. Triisopropylsilyl chloride (17.3 mL, 15.6 g, 81.0 mmol) was added dropwise then the solution was stirred for 2 h at r.t. before it was quenched with water (100 mL). The aqueous layer was extracted with EtOAc (3x) before the combined organic layers were washed with water (3x), 5% LiCl soln. (3x) and brine. The organic phase was dried over $MgSO_4$ and then the solvent was removed *in vacuo* to yield a pale-yellow oil (20.5 g).

2-ethylhexylamine (13.3 mL, 10.5 g, 81.0 mmol) and molecular sieves (4 Å) were added to a solution of the crude product from the previous step (20.5 g) in DCM (150 mL) at room temperature. The solution was stirred at r.t. until complete conversion of the aldehyde, which was monitored by ^{1}H NMR. The molecular sieves were removed, and the solvent was evaporated *in vacuo* to obtain a colourless oil.

MeOH (175 mL) was added and the solution was cooled down to 0 ºC before sodium borohydride (3.06 g, 81.0 mmol) was added. The reaction mixture was stirred at r.t. until the disappearance of the imine intermediate, as monitored by ^{1}H NMR. The solvent was removed *in vacuo* and the residue was dissolved in 1 M NaOH solution (500 mL). The solution was extracted with DCM (3x). The organic phase was dried over $MgSO_4$ and then the solvent was removed *in vacuo* to yield the crude product. The crude was purified by flash chromatography (SiO_2, 0-10% gradient of MeOH in DCM) to yield the pure product **3** as a pale-yellow oil (24.83 g, 63.4 mmol, 86% over three steps).

^{1}H NMR (400 MHz, chloroform-d): δ_H 7.15 (t, *J* = 7.7 Hz, 1H), 6.94-6.83 (m, 2H), 6.76 (ddd, *J* = 8.1, 2.5, 1.0 Hz, 1H), 3.73 (s, 2H), 2.49 (d, *J* = 6.0 Hz, 2H), 1.51-1.16 (m, 13H), 1.10 (d, *J* = 7.3 Hz, 18H), 0.89 (t, *J* = 6.9 Hz, 3H), 0.85 (t, *J* = 7.4 Hz, 3H);

^{13}C NMR (101 MHz, chloroform-d) δ_C 156.2, 142.6, 129.3, 120.8, 119.7, 118.3, 54.2, 52.5, 39.7, 31.6, 29.2, 24.7, 23.3, 18.1, 14.3, 12.8, 11.0;

HRMS (ES+): calculated for $C_{24}H_{45}NOSi$ 414.3190 $[M+Na]^+$, found 414.3197 $[M+Na]^+$;

FT-IR (ATR): ν_{max} /cm^{-1} 2959, 2867, 1601, 1586, 1485, 1463, 1277, 1154, 1004, 882, 830, 782, 685.

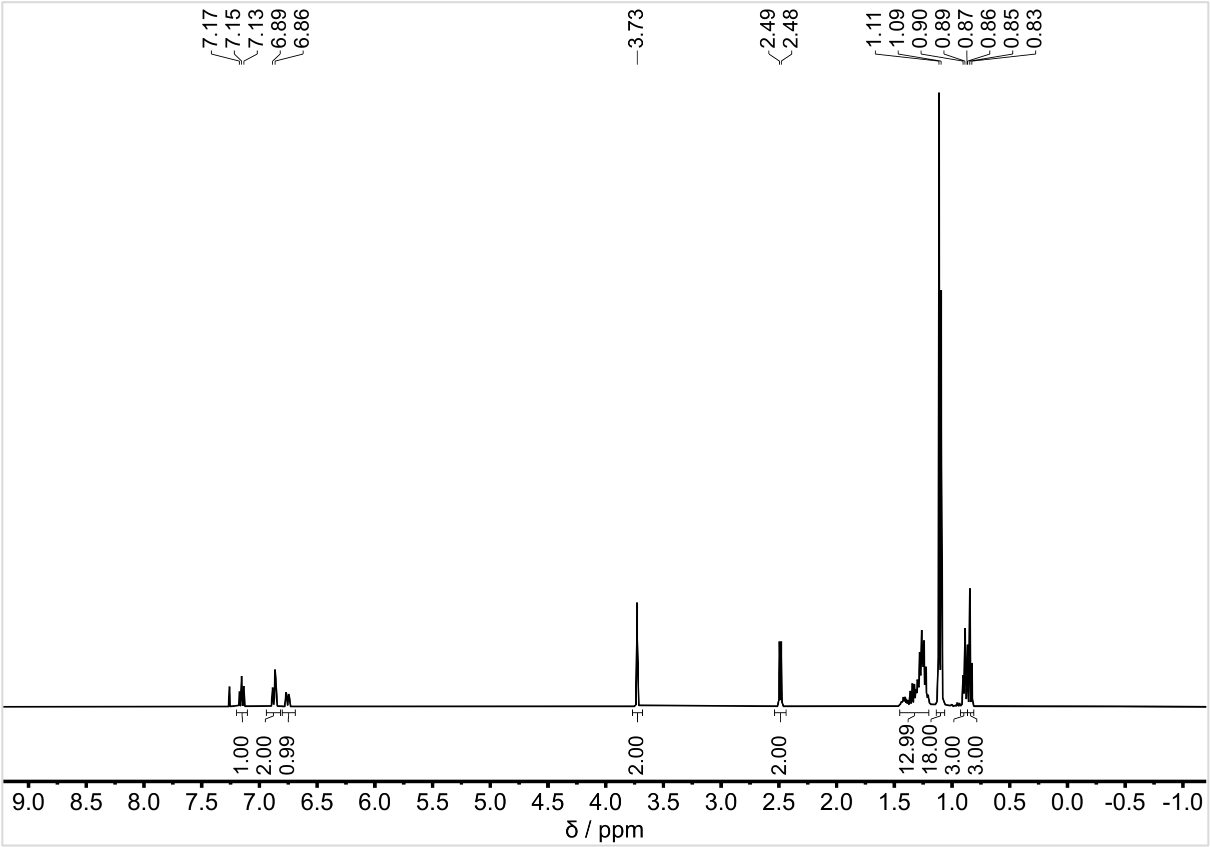

Figure S3: ^{1}H NMR (400 MHz, chloroform-d) spectrum of **3**.

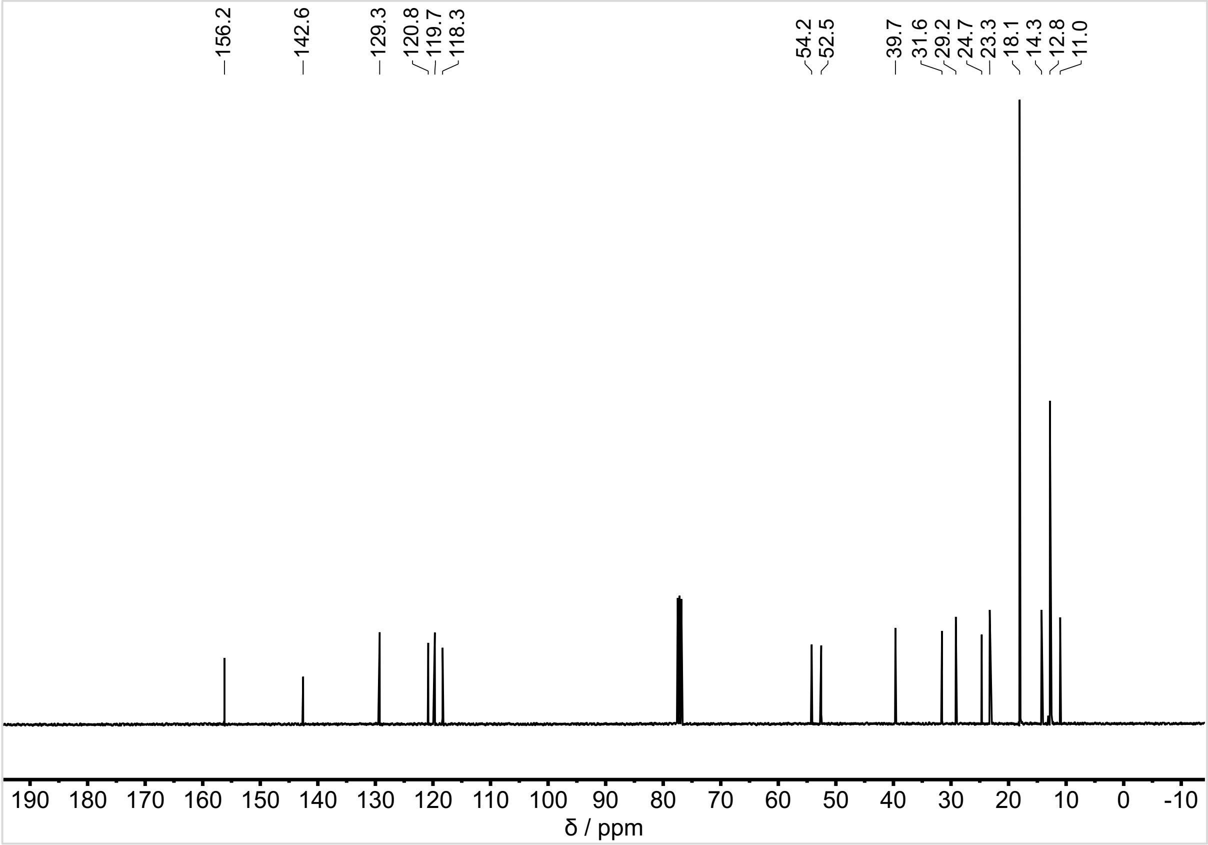

Figure S4: ^{13}C NMR (101 MHz, chloroform-d) spectrum of **3**.

Synthesis of 4

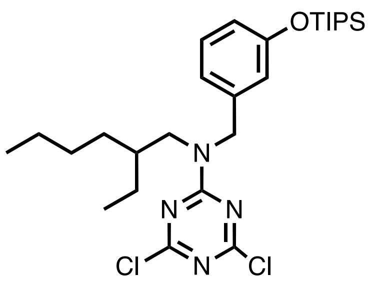

To a solution of cyanuric chloride (8.93 g, 48.4 mmol) and K_2CO_3 (12.2 g, 88.1 mmol) in THF (150 mL) at -10 °C was added dropwise a solution of **3** (17.25 g, 44.0 mmol) in THF (50 mL). The solution was stirred at -10 °C for 1 h before the solvent was removed *in vacuo* and the residue was dissolved in EtOAc. The solution was washed with 0.1 M HCl (3x) and brine before the organic phase was dried over $MgSO_4$, then the solvent was removed *in vacuo* to yield the crude product. The crude was purified by flash chromatography (SiO_2, 0-20% gradient of EtOAc in 40-60 petroleum ether) to yield the pure product **4** as a pale-yellow oil (22.76 g, 42.2 mmol, 87%).

^{1}H NMR (400 MHz, chloroform-d): δ_H 7.18 (t, *J* = 7.9 Hz, 1H), 6.88-6.75 (m, 2H), 6.72 (t, *J* = 2.1 Hz, 1H), 4.81 (d, *J* = 15.2 Hz, 1H), 4.77 (d, *J* = 15.2 Hz, 1H), 3.47 (d, *J* = 7.4 Hz, 2H), 1.86-1.72 (m, 1H), 1.39-1.13 (m, 11H), 1.07 (d, *J* = 7.2 Hz, 18H), 0.94-0.81 (m, 6H);
^{13}C NMR (101 MHz, chloroform-d) δ_C 170.2, 165.7, 156.6, 137.3, 129.9, 120.6, 119.7, 119.1, 50.5, 50.2, 37.3, 30.5, 28.5, 23.9, 23.1, 18.0, 14.2, 12.8, 10.7;
HRMS (ES+): calculated for $C_{27}H_{44}Cl_2N_4OSi$ 539.2734 $[M+H]^+$, found 539.2741 $[M+H]^+$;
FT-IR (ATR): ν_{max} /cm^{-1} 2942, 2867, 1564, 1478, 1442, 1328, 1281, 1235, 1168, 983, 884, 846, 690.

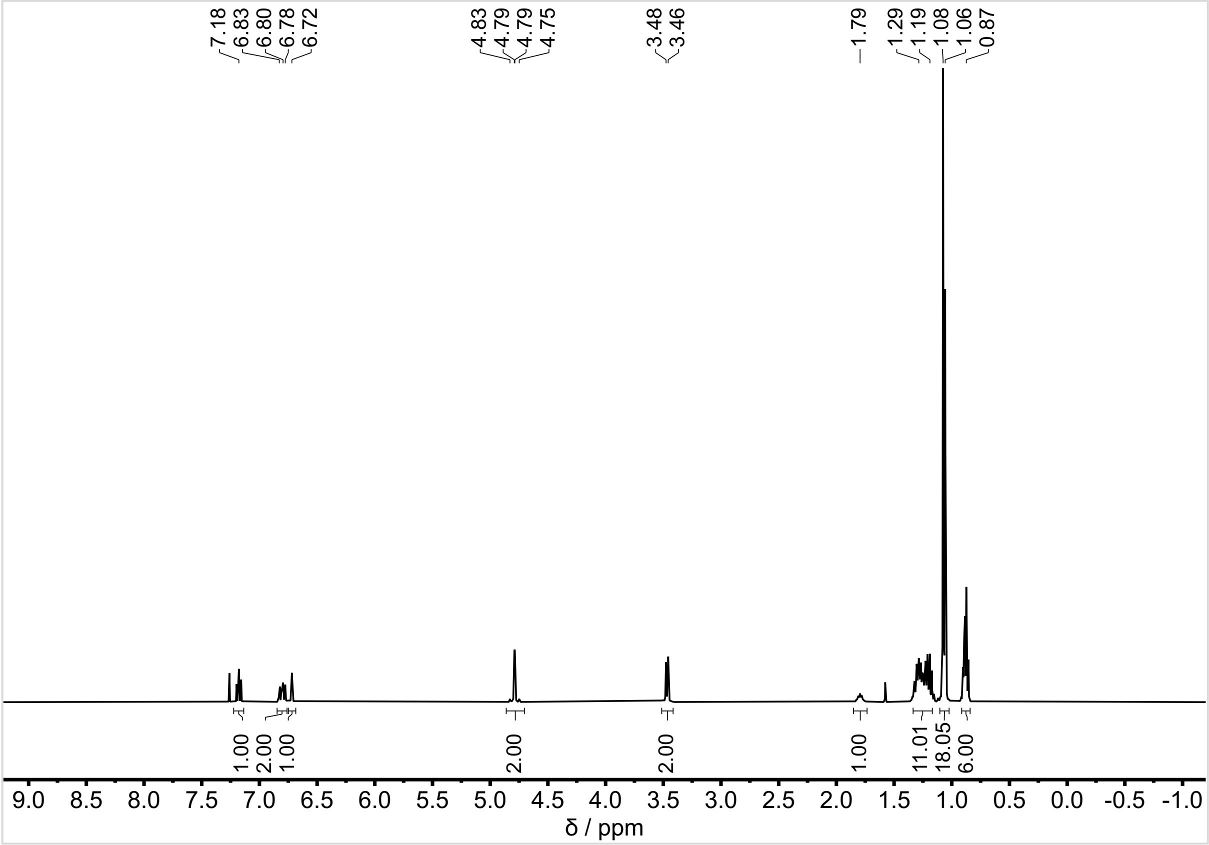

Figure S5: ^{1}H NMR (400 MHz, chloroform-d) spectrum of **4**.

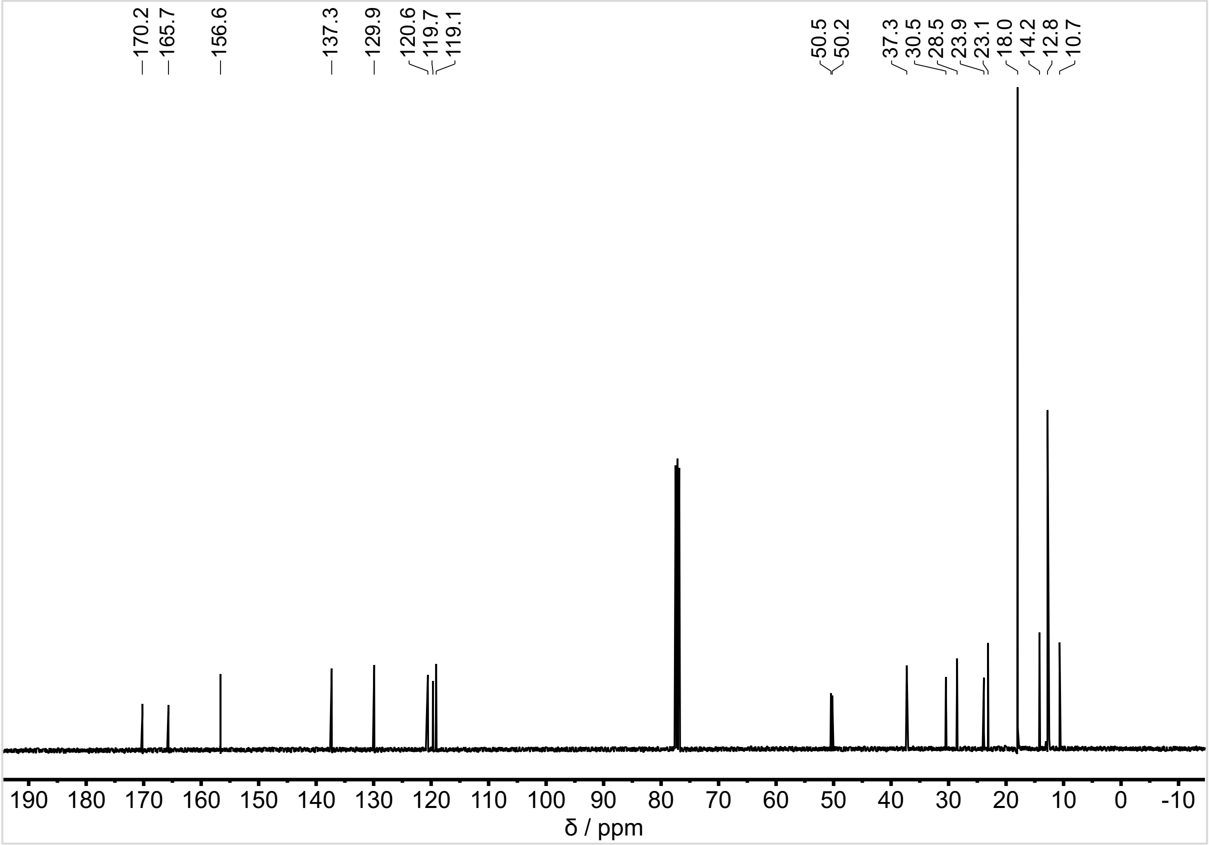

Figure S6: ^{13}C NMR (101 MHz, chloroform-d) spectrum of **4**.

Synthesis of 5

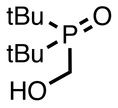

A solution of di-*tert*-butyl(chloro)phosphane (25 mL, 131.6 mmol), 37% aqueous formaldehyde (240 mL) and conc. HCl soln. (240 mL) was heated to 100 °C and refluxed overnight. The solution was cooled to 0 °C before being neutralised with NaOH (115 g) and $NaHCO_3$ (40 g), then extracted with DCM (3x). The combined organic phase was washed with brine before being dried over $MgSO_4$, then the solvent was removed *in vacuo* to yield the crude product. The crude was purified by recrystallisation with toluene to yield the pure product **5** as a white solid (16.82 g, 87.5 mmol, 66%).

^{1}H NMR (400 MHz, chloroform-d): δ_H 3.98 (d, J = 5.8 Hz, 2H), 3.85 (dd, $^3J_{PH}$ = 13 Hz, J = 5.8 Hz, 1H), 1.28 (d, $^3J_{PH}$ = 13.3 Hz, 18H).

The spectroscopic data matches that previously reported in the literature at:
Iadevaia, G., Stross, A. E., Neumann, A., Hunter, C. A.
Mix and Match Backbones for the Formation of H-bonded Duplexes. *Chem. Sci.*, **2016**, *7*, 1760.

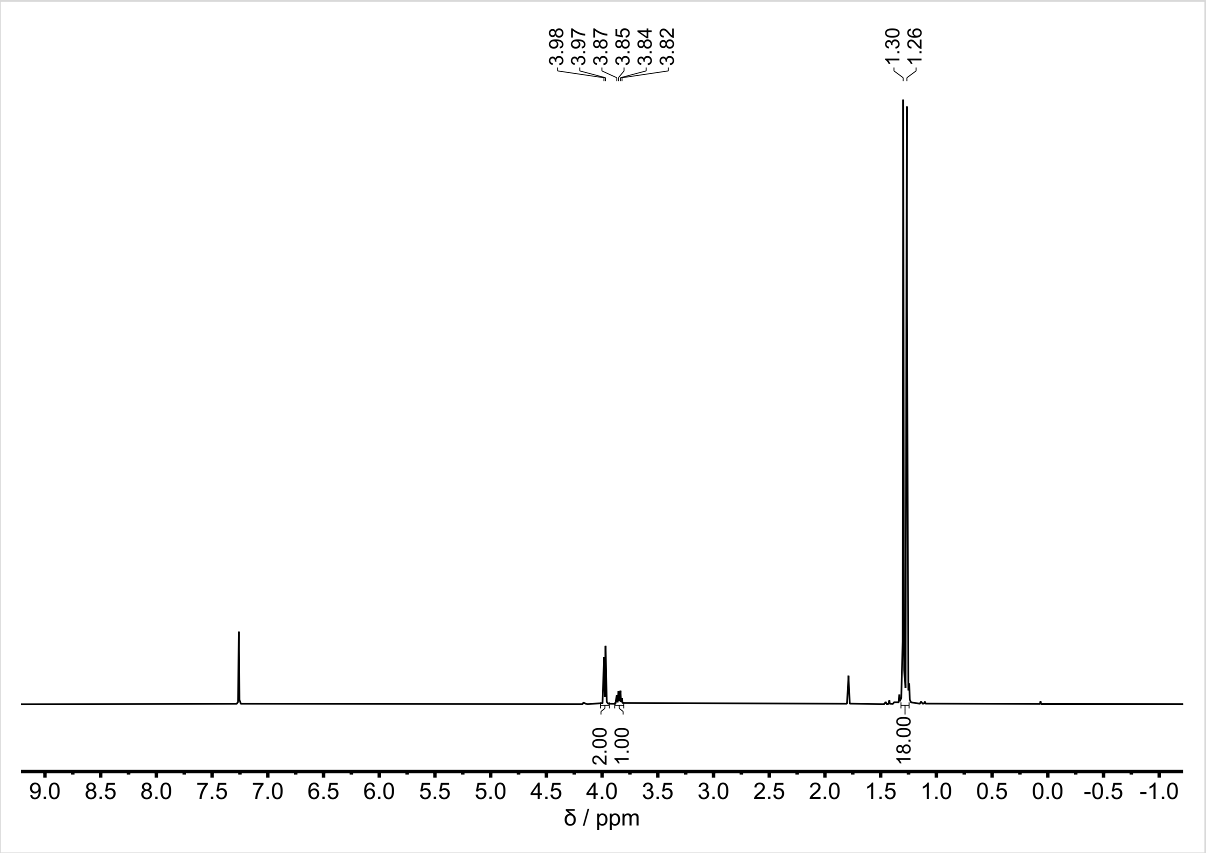

Figure S7: ^{1}H NMR (400 MHz, chloroform-d) spectrum of **5**.

Synthesis of 6

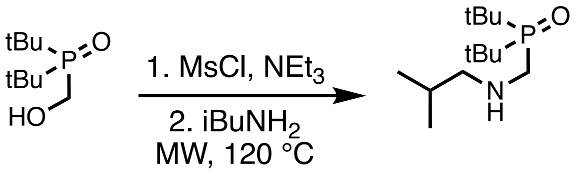

To a solution of **5** (16.82 g, 87.5 mmol) and triethylamine (18.3 mL, 13.3 g, 131.1 mmol) in dry DCM (200 mL) at 0 °C was added mesyl chloride (10.1 mL, 15.0 g, 131.1 mmol) dropwise under N_2. The reaction mixture was stirred at r.t. overnight, after which it was diluted with DCM (200 mL), washed with water (2x) and brine and then dried over $MgSO_4$. The solvent was removed *in vacuo*, and the crude was used in the next step without further purification.

The crude product obtained in the previous step was dissolved in isobutylamine (52.1 mL, 38.4 g, 524.4 mmol) and the reaction mixture was stirred at 120 °C in a microwave reactor for 2 h. The excess of isobutylamine was removed *in vacuo* and the residue was dissolved in EtOAc. The solution was washed with a sat. solution of Na_2CO_3 (3x) and then dried over $MgSO_4$ before the solvent was removed *in vacuo* to yield the product **6** as a yellow oil (20.7 g, 83.7 mmol, 96% over two steps).

The crude product was used in the next step without further purification.

^{1}H NMR (400 MHz, chloroform-d): δ_H 2.94 (d, $^2J_{PH}$ = 6.4 Hz, 2H), 2.41 (d, J = 6.7 Hz, 2H), 1.70 (non, J =6.7 Hz, 1H), 1.27 (d, $^3J_{PH}$ = 13.1 Hz, 18H), 0.89 (d, J = 6.7 Hz, 6H).

The spectroscopic data matches that previously reported in the literature at:
Troselj, P., Bolgar, P., Ballester, P., Hunter, C.A.
High Fidelity Sequence-Selective Duplex Formation by Recognition-Encoded Melamine Oligomers. *J. Am. Chem. Soc.* **2021**, *143,* 8669–8678.

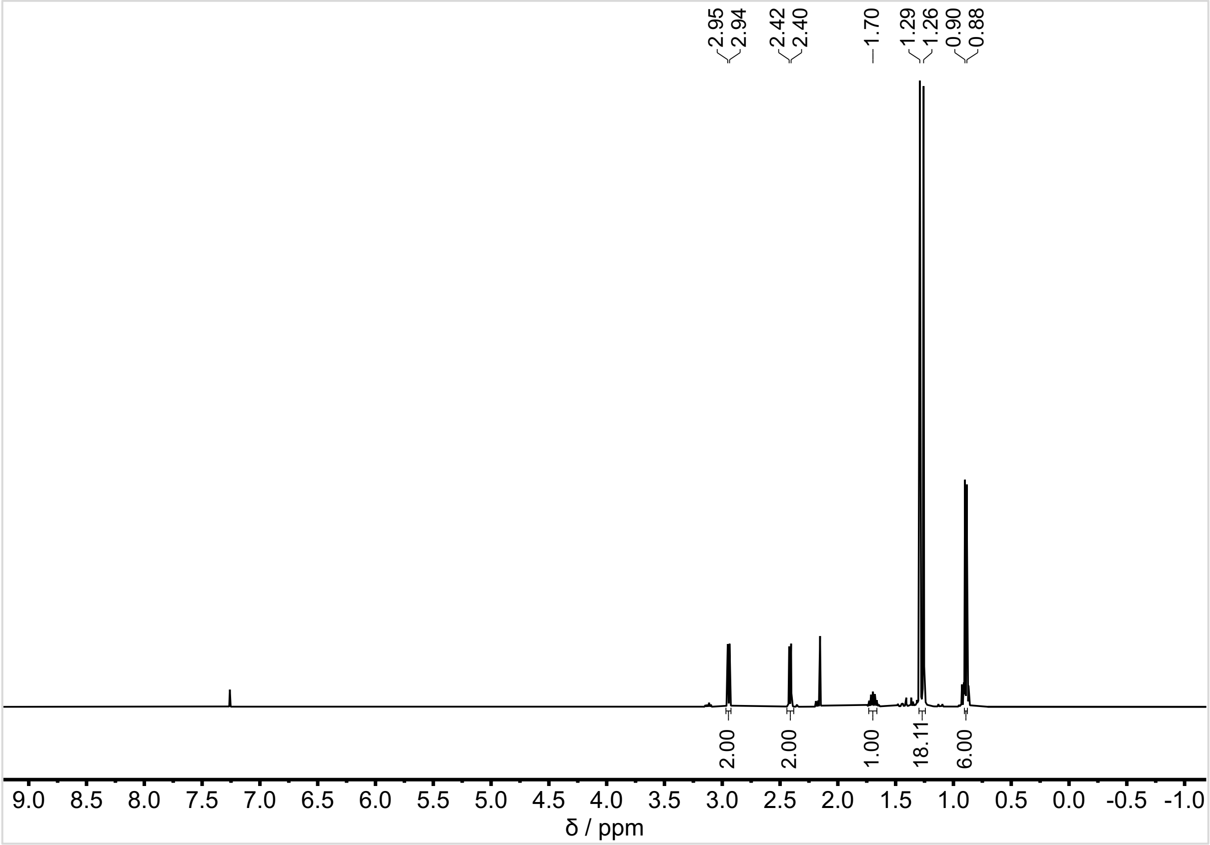

Figure S8: ^{1}H NMR (400 MHz, chloroform-d) spectrum of **6**.

Synthesis of 7

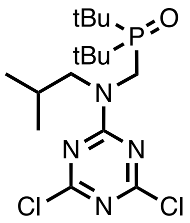

To a solution of cyanuric chloride (17.0 g, 92.1 mmol) and K_2CO_3 (23.1 g, 167.4 mmol) in THF (320 mL) at -10 °C was added dropwise a solution of **6** (20.7 g, 83.7 mmol) in THF (80 mL).The solution was stirred at -10 °C for 1 h. The solvent was removed *in vacuo* and the residue was dissolved in EtOAc. The solution was washed with 0.1 M HCl (3x) and brine before the organic phase was dried over $MgSO_4$, then the solvent was removed *in vacuo* to yield the crude product. The crude was purified by flash chromatography (SiO_2, 0-80% gradient of EtOAc in 40-60 petroleum ether) to yield the pure product **7** as a yellow foam (29.3 g, 74.1 mmol, 89%).

^{1}H NMR (400 MHz, chloroform-d): δ_H 4.30 (d, $^2J_{PH}$ = 3.7 Hz, 1H), 3.97 (d, J = 7.4 Hz, 2H), 2.18 (non, J = 6.9 Hz, 1H), 1.28 (d, $^3J_{PH}$ = 13.1 Hz, 18H), 0.92 (d, J = 6.9 Hz, 6H).

The spectroscopic data matches that previously reported in the literature at:
Troselj, P., Bolgar, P., Ballester, P., Hunter, C.A.
High Fidelity Sequence-Selective Duplex Formation by Recognition-Encoded Melamine Oligomers. *J. Am. Chem. Soc.* **2021**, *143,* 8669–8678.

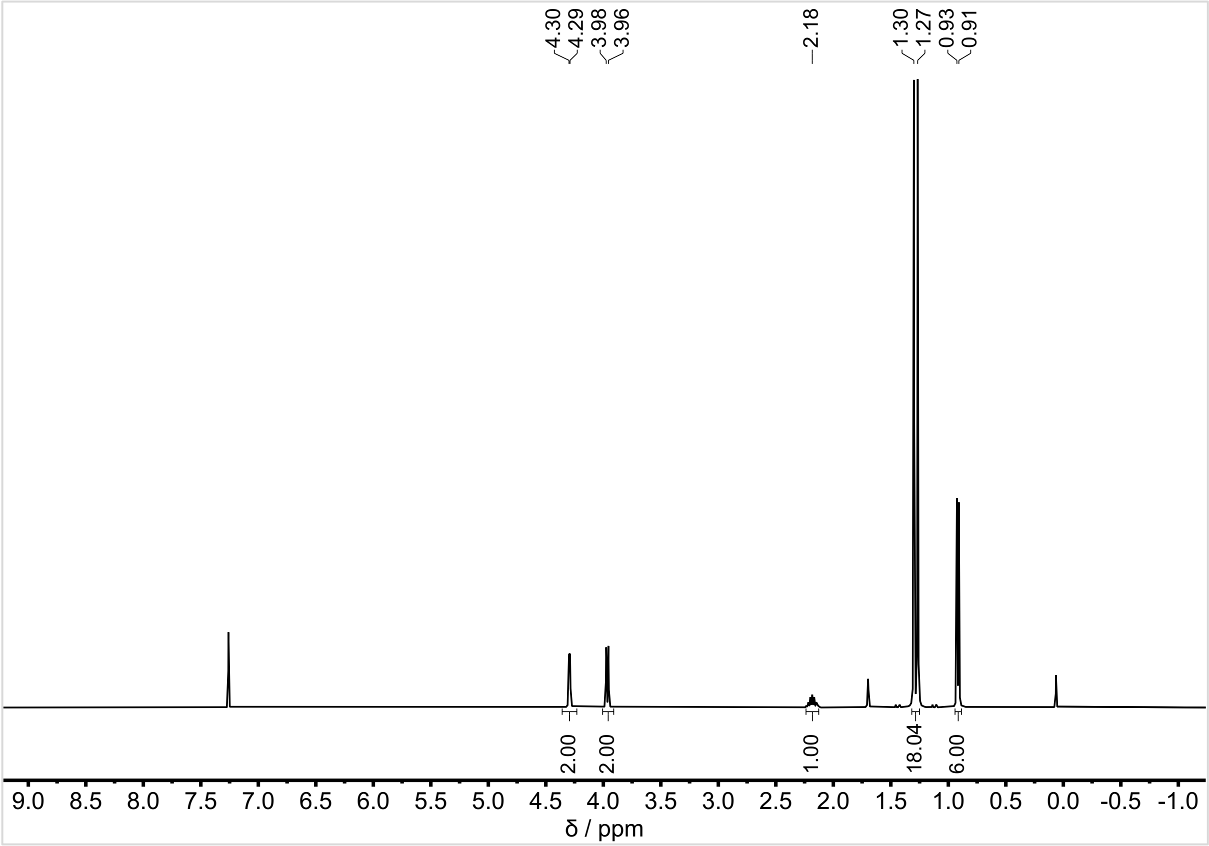

Figure S9: ^{1}H NMR (400 MHz, chloroform-d) spectrum of **7**.

Synthesis of 8

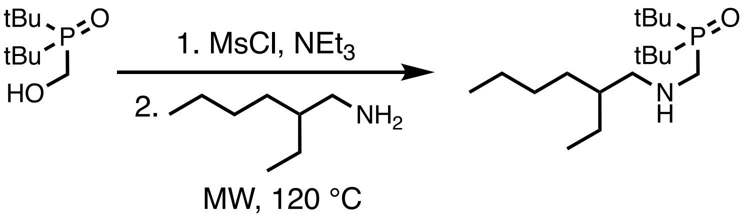

To a solution of **5** (1.55 g, 8.06 mmol) and triethylamine (1.68 mL, 1.22 g, 12.1 mmol) in dry DCM (20 mL) at 0 °C was added mesyl chloride (0.99 mL, 1.39 g, 12.1 mmol) dropwise under N_2. The reaction mixture was stirred at r.t. overnight, after which it was diluted with DCM (20 mL), washed with water (2x) and brine and then dried over $MgSO_4$. The solvent was removed *in vacuo*, and the crude was used in the next step without further purification.

The crude product obtained in the previous step was dissolved in 2-ethylhexylamine (9.12 mL, 7.19 g, 55.6 mmol) and the reaction mixture was stirred at 120 °C in a microwave for 2 h. The residue was dissolved in EtOAc and the resultant solution was washed with sat. solution of Na_2CO_3 (3x) then dried over $MgSO_4$ before the solvent was removed *in vacuo* to yield the crude product. The excess 2-ethylhexylamine was removed by flash chromatography (SiO_2, 0-10% gradient of MeOH in DCM) to yield the product **8** as a yellow oil (2.31 g).

The product contained some impurities and was used in the next step without further purification.

^{1}H NMR (400 MHz, chloroform-d): δ_H 2.91 (d, $^2J_{PH}$ = 6.4 Hz, 2H), 2.46 (d, J = 5.5 Hz, 2H), 1.43-1.11 (m, 9H), 1.24 (d, $^3J_{PH}$ = 13.1 Hz, 18H), 0.86-0.76 (m, 6H);
^{31}P NMR (162 MHz, chloroform-d): δ_P 58.01.

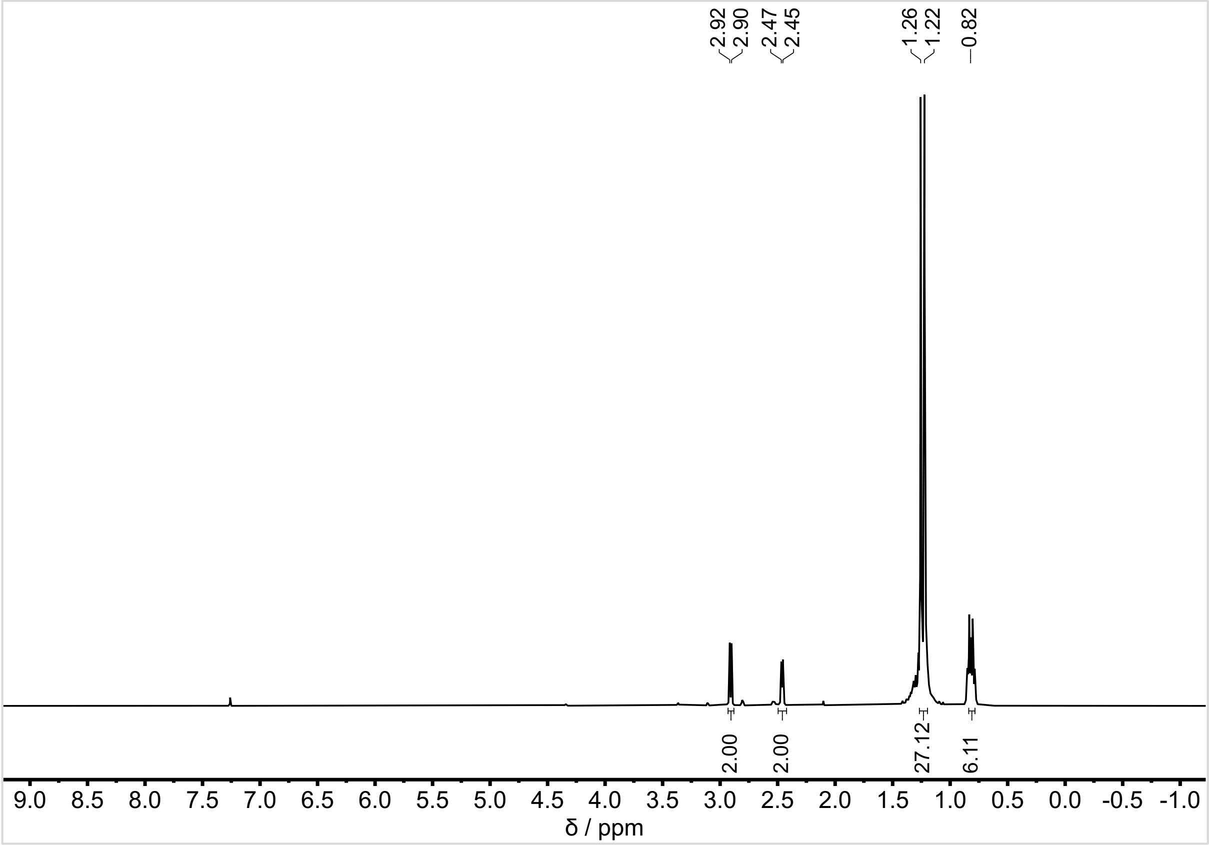

Figure S10: ^{1}H NMR (400 MHz, chloroform-d) spectrum of impure **8**.

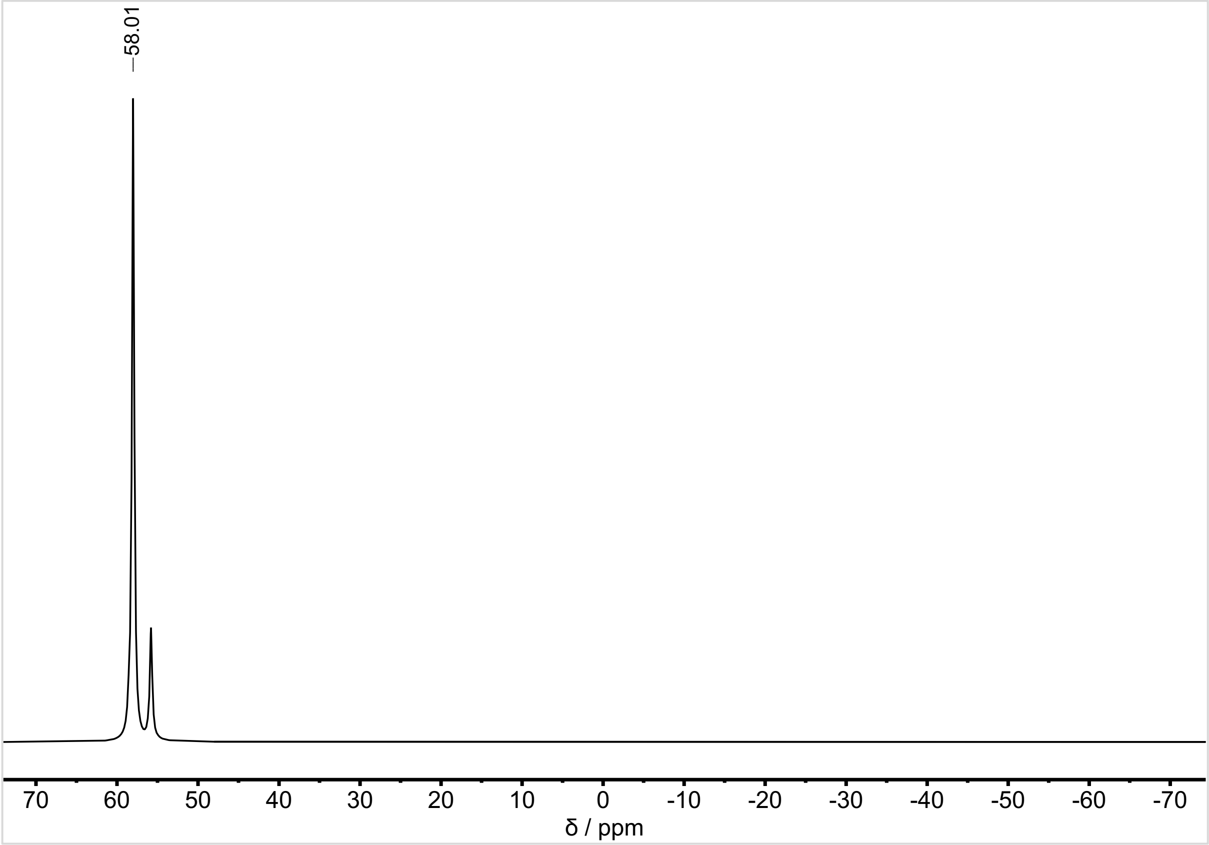

Figure S11: ^{31}P NMR (162 MHz, chloroform-d) spectrum of impure **8**.

Synthesis of 9

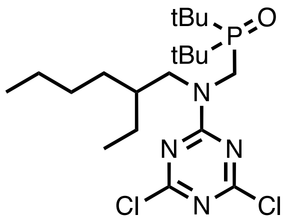

To a solution of cyanuric chloride (1.54 g, 8.37 mmol) and K_2CO_3 (2.10 g, 15.2 mmol) in THF (40 mL) at -10 °C was added dropwise a solution of **8** (2.31 g) in THF (10 mL).The solution was stirred at -10 °C for 1 h. The solvent was removed *in vacuo* and the residue was dissolved in EtOAc. The solution was washed with 0.1 M HCl (3x) and brine before the organic phase was dried over $MgSO_4$, then the solvent was removed *in vacuo* to yield the crude product. The crude was purified by flash chromatography (SiO_2, 0-50% gradient of EtOAc in 40-60 petroleum ether) to yield the pure product **9** as a yellow oil (3.25 g, 7.19 mmol, 89%).

^{1}H NMR (400 MHz, chloroform-d): δ_H 4.30 (dd, $^2J_{HH}$ = 15.7, $^2J_{PH}$ = 3.5 Hz, 1H), 4.25 (dd, $^2J_{HH}$ = 15.7, $^2J_{PH}$ = 3.5 Hz, 1H), 4.06 (d, J = 7.4 Hz, 2H), 1.88-1.81 (m, 1H), 1.39-1.17 (m, 8H), 1.27 (d, $^3J_{PH}$ = 13.4 Hz, 18H), 0.90 (t, J = 7.4 Hz, 3H), 0.87 (t, J = 6.7 Hz, 3H);
^{13}C NMR (101 MHz, chloroform-d) δ_C 170.4 & 170.0 (rotamers), 165.1 & 165.1 (rotamers), 50.7, 38.4 (d, $^1J_{PC}$ = 53.2 Hz), 36.7, 36.3 (d, $^1J_{PC}$ = 56.5 Hz), 30.4, 28.3, 26.8, 23.8, 23.2, 14.1, 10.7;
^{31}P NMR (162 MHz, chloroform-d) δ_P 59.29;
HRMS (ES+): calculated for $C_{20}H_{37}Cl_2N_4OP$ 451.2155 $[M+H]^+$, found 451.2156 $[M+H]^+$;
FT-IR (ATR): ν_{max} /cm^{-1} 2957, 2929, 2872, 1567, 1476, 1371, 1349, 1328, 1235, 1184, 1163, 1058, 846, 830, 796.

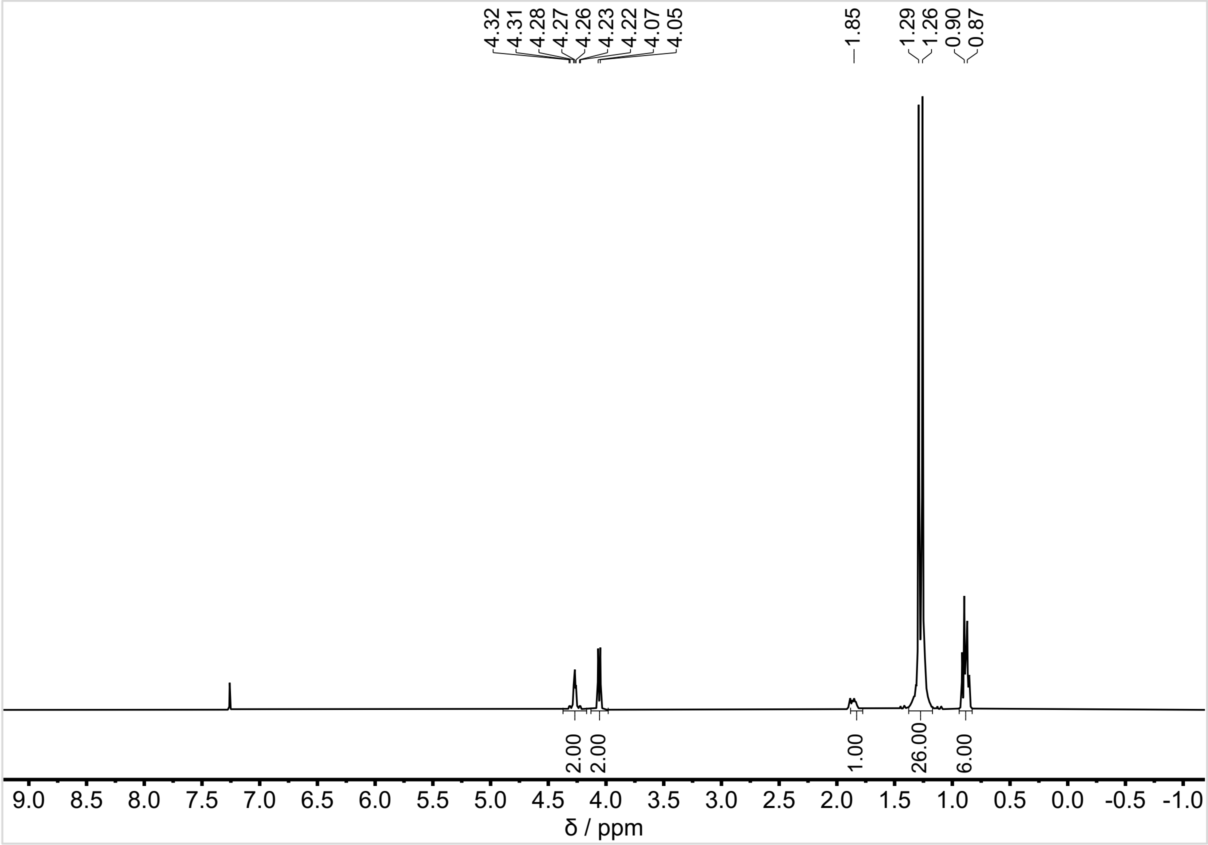

Figure S12: ^{1}H NMR (400 MHz, chloroform-d) spectrum of **9**.

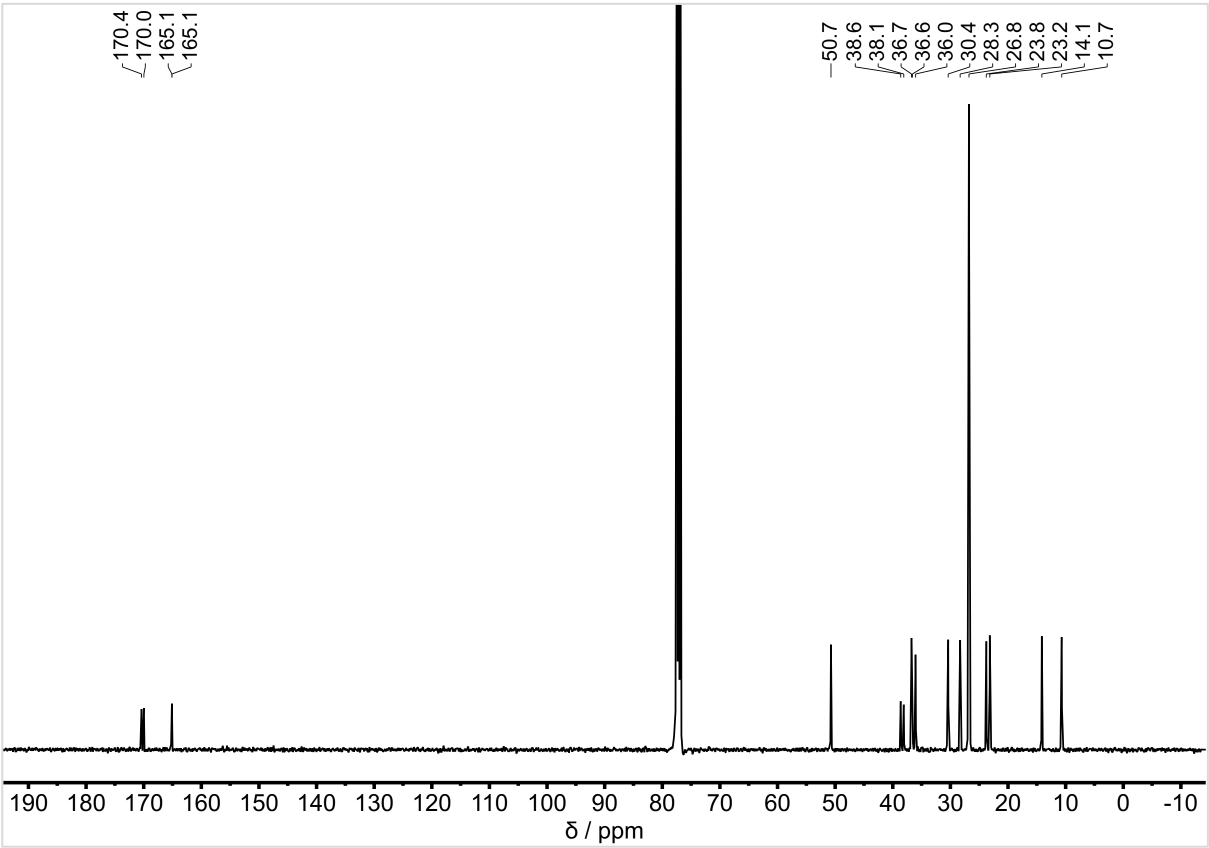

Figure S13: ^{13}C NMR (101 MHz, chloroform-d) spectrum of **9**.

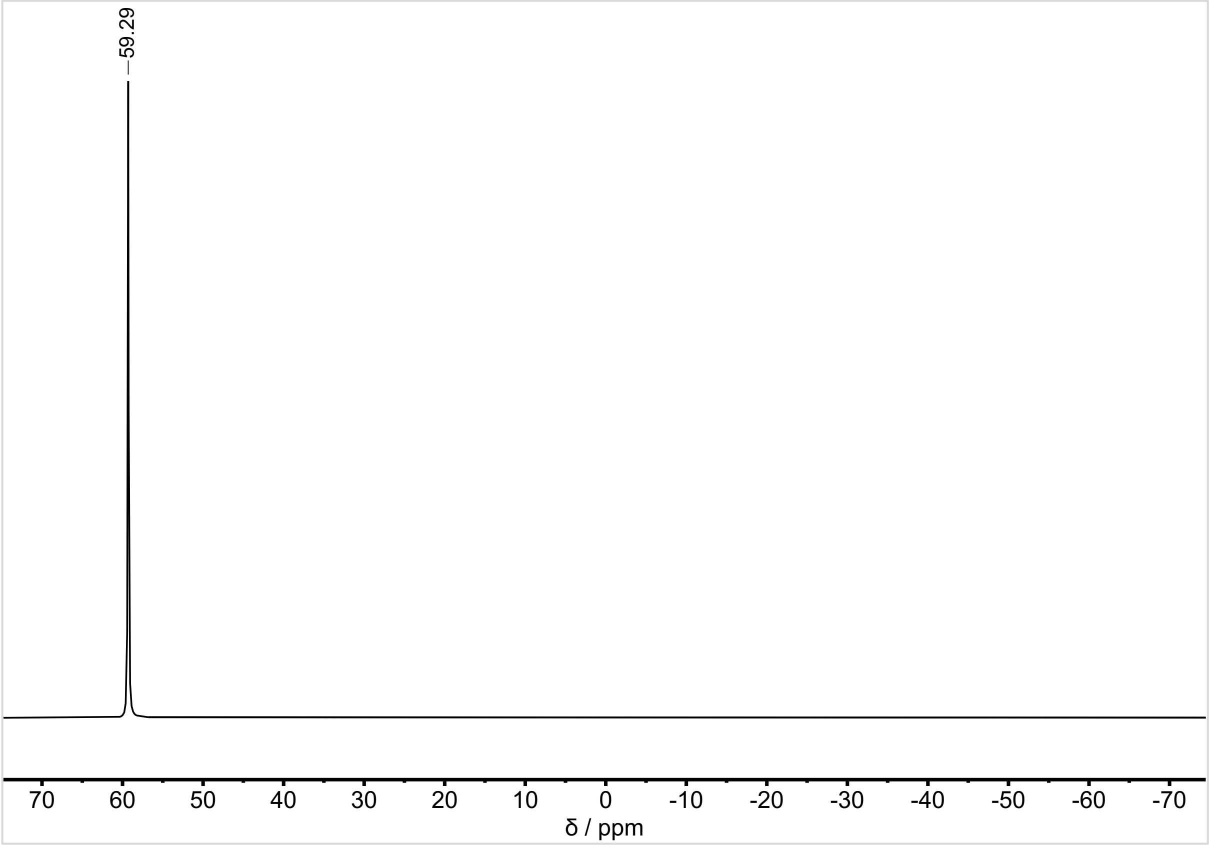

Figure S14: ^{31}P NMR (162 MHz, chloroform-d) spectrum of **9**.

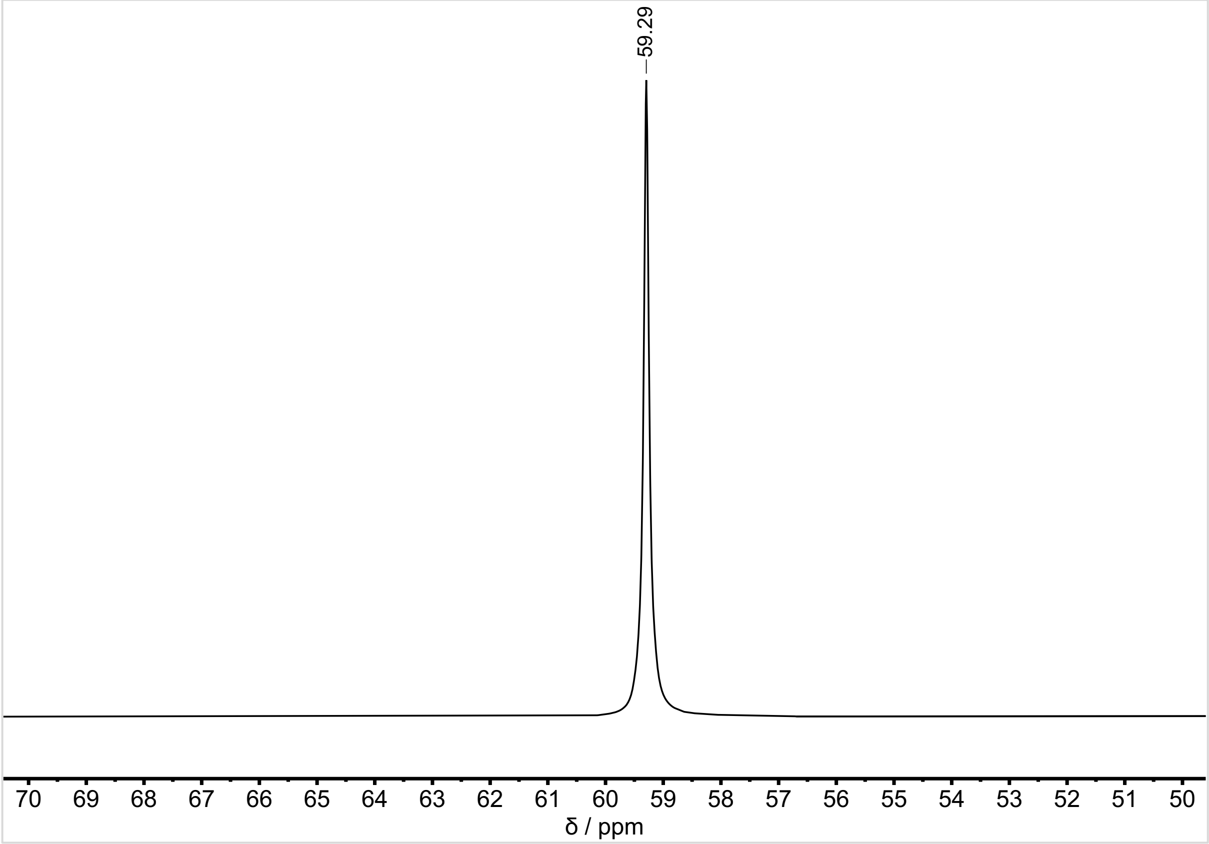

Figure S15: ^{31}P NMR (162 MHz, chloroform-d) spectrum of **9**.

Synthesis of 10

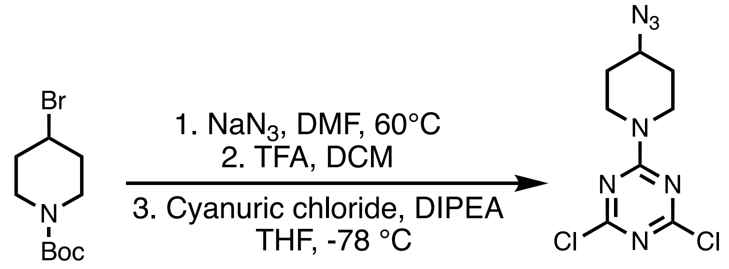

A solution of 1-Boc-4-bromopiperidine (400 mg, 1.51 mmol) and sodium azide (197 mg, 3.03 mmol) in DMF (5 mL) was stirred overnight at 60 °C under N_2 atmosphere. The mixture was diluted with water (20 mL) then extracted with EtOAc (3x). The combined organic layers were washed with water (3x), 5% LiCl soln. (3x) and brine. The organic phase was dried over $MgSO_4$ and then the solvent was removed *in vacuo* to obtain a colourless oil.

The obtained product was dissolved in DCM (6 mL) before TFA (2 mL) was added and the mixture was stirred at r.t. for 30 mins. The solvent and reagent were removed by flushing the mixture under N_2 to obtain a colourless oil.

To a solution of cyanuric chloride (419 mg, 2.27 mmol) in THF (40 mL) at -78 °C was added dropwise a solution of the crude obtained in the previous step and DIPEA (1.05 mL, 781 mg, 6.04 mmol) in THF (10 mL). The solution was stirred at -78 °C for 1 h. The solvent was removed *in vacuo* and the residue was dissolved in EtOAc. The solution was washed with 0.1 M HCl (3x) and brine before the organic phase was dried over $MgSO_4$, then the solvent was removed *in vacuo* to yield the crude product. The crude was purified by flash chromatography (SiO_2, 0-20% gradient of EtOAc in 40-60 petroleum ether) to yield the pure product **10** as a white solid (316 mg, 1.15 mmol, 76% over three steps).

^{1}H NMR (400 MHz, chloroform-d): δ_H 4.19-4.09 (m, 2H), 3.84-3.73 (m, 1H), 3.73-3.62 (m, 2H), 2.02-1.90 (m, 2H), 1.76-1.63 (m, 2H);
^{13}C NMR (101 MHz, chloroform-d): δ_C 170.5, 164.0, 56.7, 41.5, 30.4;
HRMS (ES+): calculated for $C_8H_9Cl_2$ 274.0369 $[M+H]^+$, found 274.0371 $[M+H]^+$;
FT-IR (ATR): ν_{max} /cm^{-1} 2931, 2874, 2092, 1572, 1475, 1349, 1326, 1232, 1194, 1170, 1154, 1134, 1093, 1066.

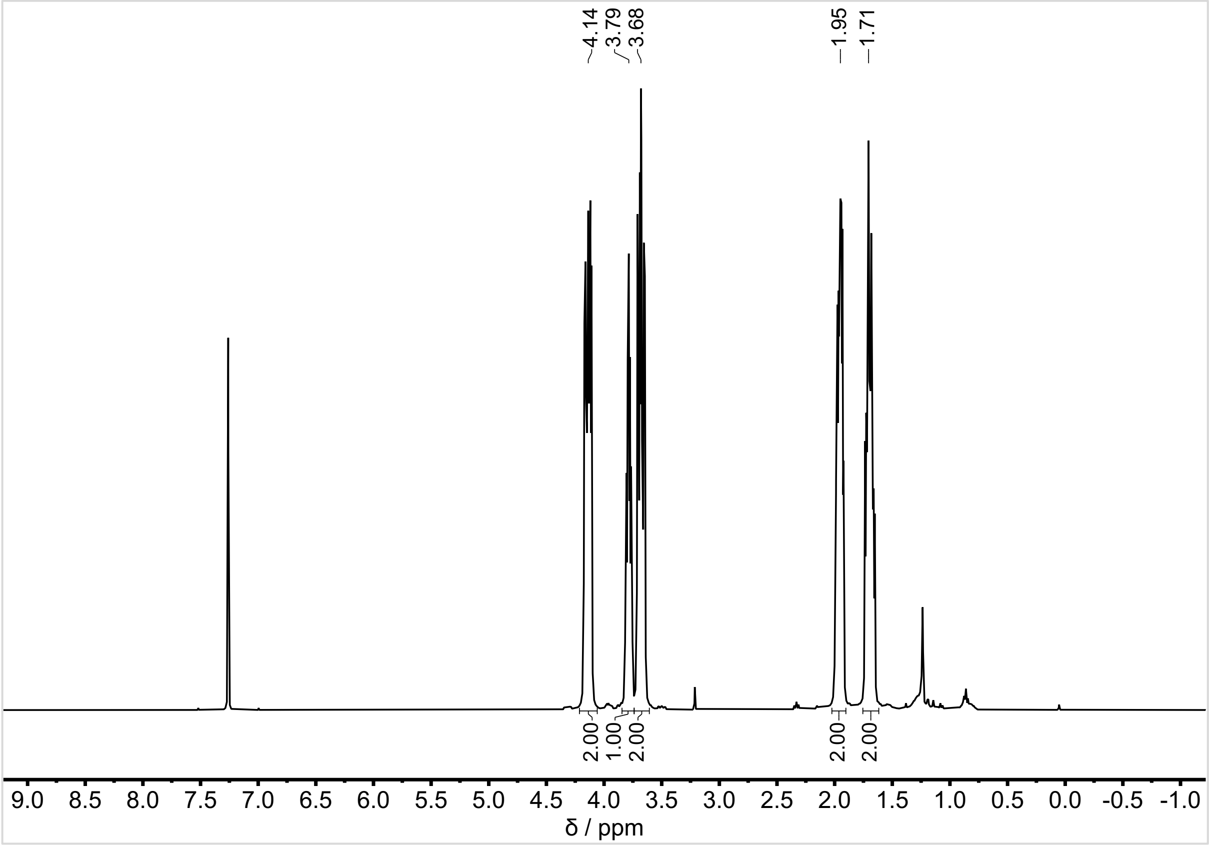

Figure S16: ^{1}H NMR (400 MHz, chloroform-d) spectrum of **10**.

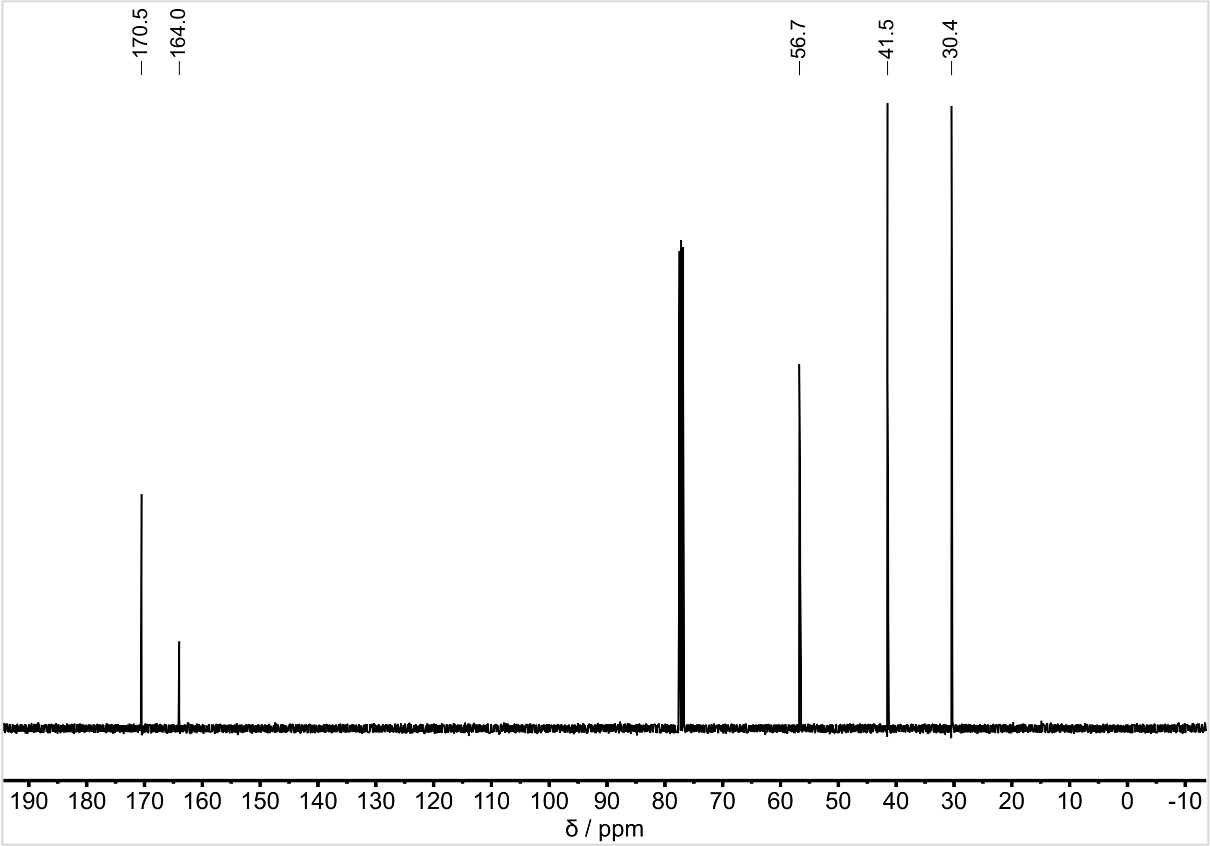

Figure S17: ^{13}C NMR (101 MHz, chloroform-d) spectrum of **10**.

Synthesis of 11

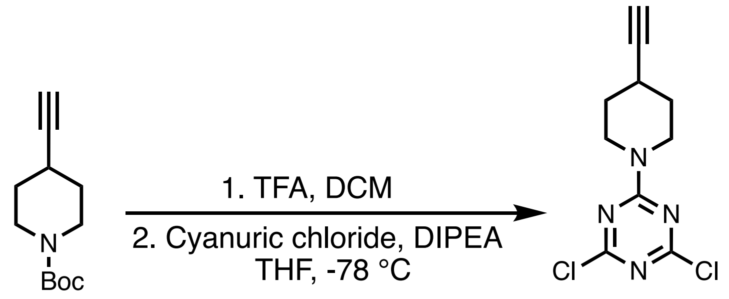

To a solution of 1-Boc-4-ethynylpiperidine (200 mg, 0.96 mmol) in DCM (1 mL) was added TFA (3 mL) before the mixture was stirred at r.t. for 30 mins. The solvent and reagent were removed by flushing the mixture under N_2 to obtain a colourless oil.

To a solution of cyanuric chloride (264 mg, 1.43 mmol) in THF (40 mL) at -78 °C was added dropwise a solution of the crude obtained in the previous step and DIPEA (0.33 mL, 247 mg, 1.91 mmol) in THF (10 mL). The solution was stirred at -78 °C for 1 h. The solvent was removed *in vacuo* and the residue was dissolved in EtOAc. The solution was washed with 0.1 M HCl (3x) and brine before the organic phase was dried over $MgSO_4$, then the solvent was removed *in vacuo* to yield the crude product. The crude was purified by flash chromatography (SiO_2, 0-20% gradient of EtOAc in 40-60 petroleum ether) to yield the pure product **11** as a white solid (238 mg, 0.93 mmol, 97% over two steps).

^{1}H NMR (400 MHz, chloroform-d): δ_H 4.06-3.95 (m, 2H), 3.85-3.75 (m, 2H), 2.82-2.73 (m, 1H), 2.15 (d, J = 2.3 Hz, 1H), 1.92-1.81 (m, 2H), 1.78-1.66 (m, 2H);
^{13}C NMR (101 MHz, chloroform-d): δ_C 170.4, 163.8, 85.2, 70.6, 42.4, 31.0, 26.4;
HRMS (ES+): calculated for $C_{10}H_{10}Cl_2N_2$ 257.0355 $[M+H]^+$, found 257.0354 $[M+H]^+$;
FT-IR (ATR): ν_{max} /cm^{-1} 3225, 2959, 1559, 1461, 1351, 1325, 1273, 1250, 1226, 1192, 1165, 1150, 1082.

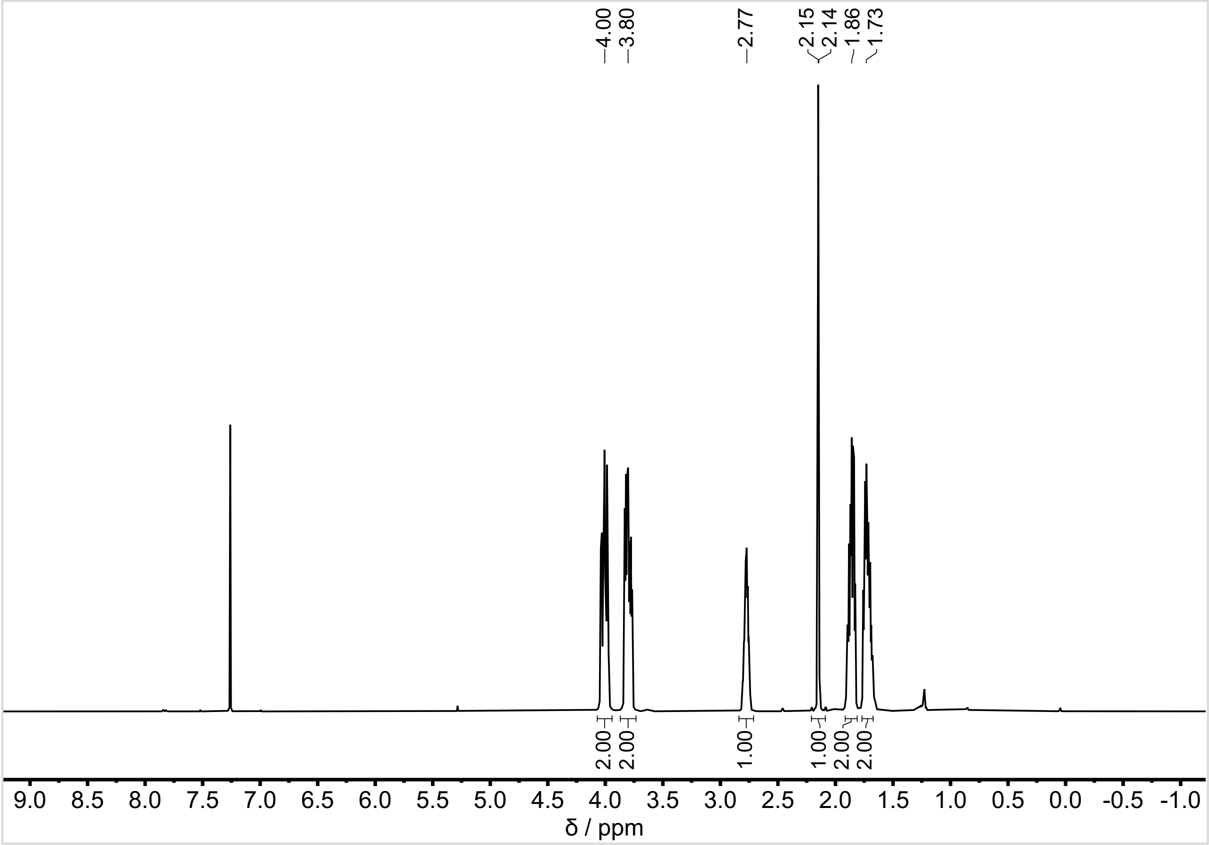

Figure S18: ^{1}H NMR (400 MHz, chloroform-d) spectrum of **11**.

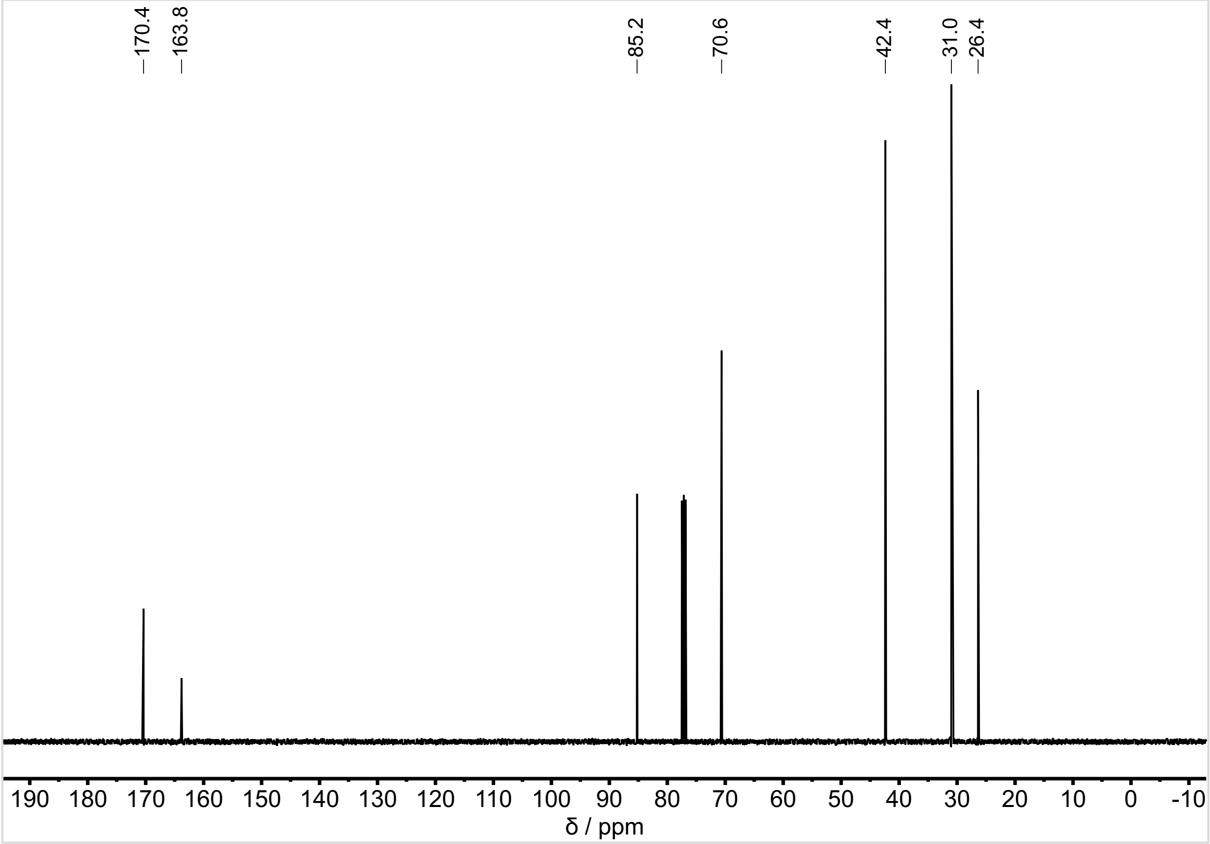

Figure S19: ^{13}C NMR (101 MHz, chloroform-d) spectrum of **11**.

Synthesis of 12

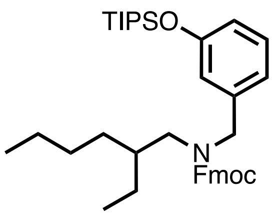

To a solution of **3** (2.00 g, 5.11 mmol) in DCM (20 mL) at 0 °C was added Fmoc chloride (1.45 g, 5.62 mmol), then triethylamine (0.79 mL, 0.57 g, 5.62 mmol) was added dropwise. The reaction was stirred for 2 h at r.t. before the mixture was washed with 1 M K_2CO_3 solution and brine. The organic phase was dried over $MgSO_4$ and then the solvent was removed *in vacuo* to yield the crude product as a yellow oil. The crude was purified by flash chromatography (SiO_2, 0-10% gradient of EtOAc in 40-60 petroleum ether) to yield the product **12** (3.04 g, 4.95 mmol, 97%) as a colourless oil.

The NMR spectra are consistent with the presence of two slowly exchanging rotamers in solution. Where rotamers are distinguishable, the corresponding peaks are listed together.

^{1}H NMR (400 MHz, chloroform-d): δ_H 7.76 & 7.73 (d, *J* = 7.6 Hz, 2H, rotamers), 7.61 & 7.47 (d, *J* = 7.4 Hz, 2H, rotamers)7.42-7.34 (m, 2H), 7.31 & 7.23 (t, *J* = 7.5 Hz, 1H), 7.17-7.12 (m, 1H), 6.81-6.76 (m, 1H), 6.76–6.67 (m, 2H), 4.58 & 4.43 (d, *J* = 5.5 Hz, 2H, rotamers), 4.43 & 4.42 (s, 2H), 4.27-4.18, (m, 1H), 3.20 & 2.90 (m & d, *J* = 7.5 Hz, 2H, rotamers), 1.71-1.61 & 1.50-1.41 (m, 1H, rotamers), 1.33-1.05 (m, 8H), 1.23 (m, 3H), 1.08 (d, *J* = 7.3 Hz, 18H), 0.91-0.83 & 0.74-0.69 (m, 6H, rotamers);
^{13}C NMR (101 MHz, chloroform-d) δ_C 157.2 & 156.5 (rotamers), 156.7 & 156.4 (rotamers), 144.2, 141.5 & 141.4 (rotamers), 139.4, 129.7 & 129.6 (rotamers), 127.7 & 127.1 (rotamers), 125.2 & 124.9 (rotamers), 120.6 & 119.8 (rotamers), 120.0, 119.4 & 118.6 (rotamers), 118.9 & 118.8 (rotamers), 67.5 & 67.0 (rotamers), 50.7 & 49.6 (rotamers), 50.5 & 50.2 (rotamers), 47.6 & 47.5 (rotamers), 37.6 & 37.5 (rotamers), 30.6 & 30.3 (rotamers), 28.8 & 28.5 (rotamers), 23.9 & 23.5 (rotamers), 23.2, 18.0, 14.3 & 14.2 (rotamers), 12.8, 10.7 & 10.6 (rotamers);
HRMS (ES+): calculated for $C_{39}H_{55}NO_3Si$ 614.4024 $[M+H]^+$, found 614.4042 $[M+H]^+$;
FT-IR (ATR): ν_{max} /cm^{-1} 2958, 2944, 2934, 2892, 2867, 1703, 1603, 1486, 1463, 1449, 1421, 1279, 1231.

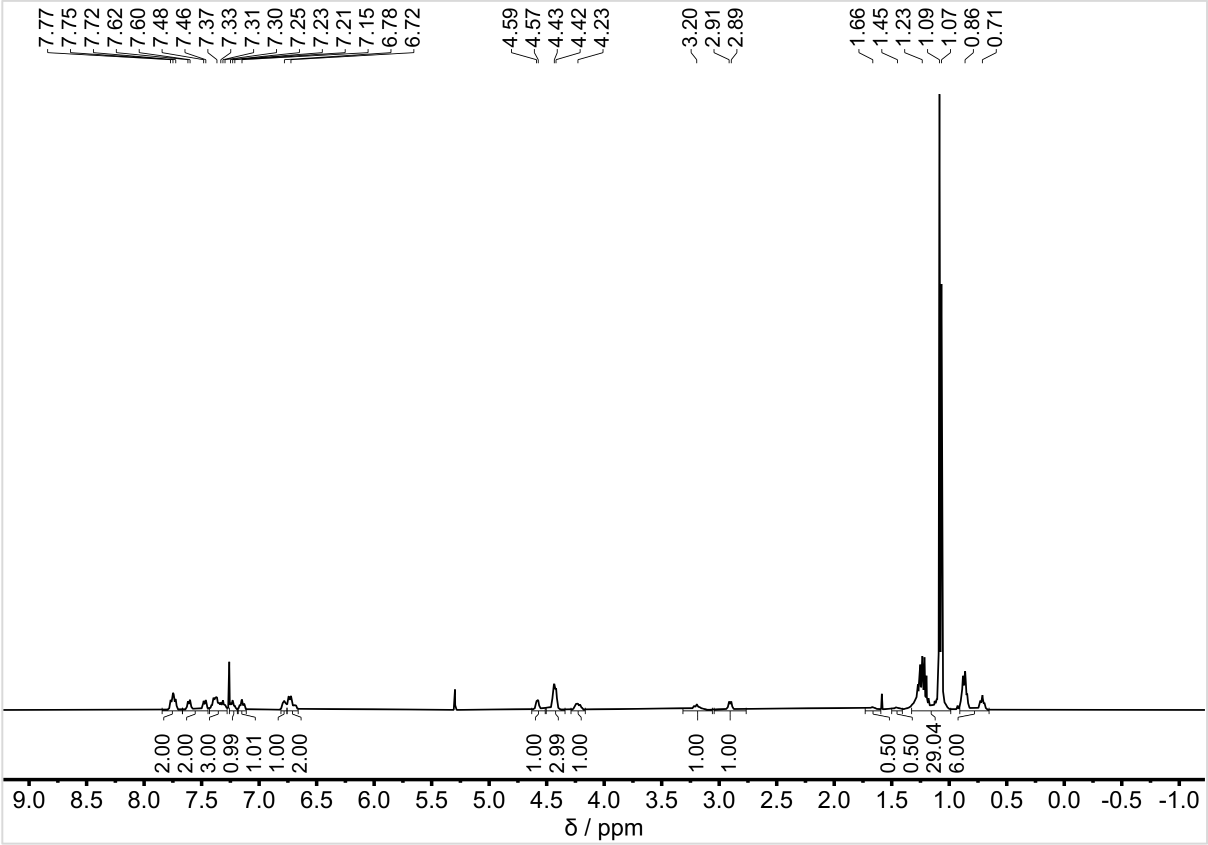

Figure S20: ^{1}H NMR (400 MHz, chloroform-d) spectrum of **12**.

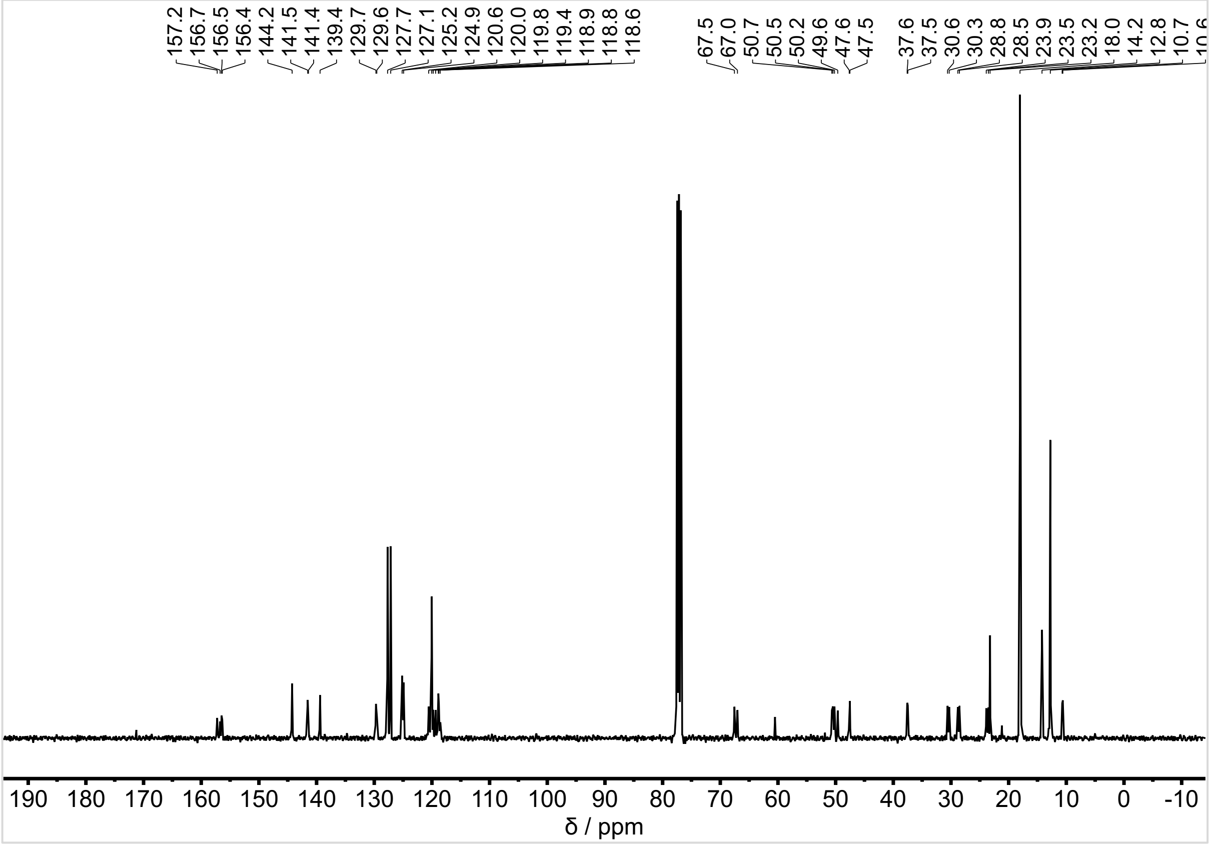

Figure S21: ^{13}C NMR (101 MHz, chloroform-d) spectrum of **12**.

Synthesis of 13

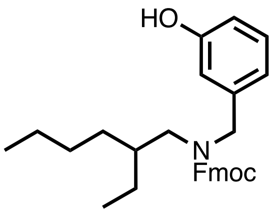

12 (3.00g, 4.89 mmol) was dissolved in THF (40 mL) and the solution was acidified to pH 3 by adding 1 M AcOH. TBAF (9.78 mL, 9.78 mmol, 1 M in THF) was added dropwise and the reaction mixture was stirred at r.t. until complete conversion of the starting material, as monitored by LCMS (approx. 2 h). After completion, the reaction was quenched with 5% aq. soln. HCl and extracted with EtOAc (3x) followed by washing with 5% aq. soln. HCl (3x) and brine. The organic phase was dried over $MgSO_4$ and the solvent was removed *in vacuo* to yield the crude product. The crude was purified by flash chromatography (SiO_2, 0-40% gradient of EtOAc in 40-60 petroleum ether) to yield the pure product **13** (1.85 g, 4.05 mmol, 83%) as a colourless foam.

The NMR spectra are consistent with the presence of two slowly exchanging rotamers in solution. Where rotamers are distinguishable, the corresponding peaks are listed together.

^{1}H NMR (400 MHz, chloroform-d): δ_H 7.74 & 7.70 (d, *J* = 7.5 Hz, 2H, rotamers), 7.55 & 7.41 (d, *J* = 7.5 Hz, 2H, rotamers), 7.39-7.32 (m, 2H), 7.29c & 7.22 (t, *J* = 7.5 Hz, 2H, rotamers), 7.18-7.10 (m, 1H), 6.75 (t, *J* = 8.0 Hz, 1H), 6.68-6.60 (m, 1H), 6.67 & 6.47 (s, 1H, rotamers), 5.00 (br s, 1H), 4.58 & 4.49 (d, *J* = 4.8, *J* = 6.8 Hz, 2H, rotamers), 4.37 & 4.30 (s, 2H, rotamers), 4.21-4.15, (m, 1H), 3.15 & 2.84 (m & d, *J* = 7.6 Hz, 2H, rotamers), 1.63 & 1.40 (m, 1H), 1.32-0.97 (m, 8H), 0.88-0.80 & 0.71-0.65 (m, 6H, rotamers);
^{13}C NMR (101 MHz, chloroform-d): δ_C 157.5 & 156.9 (rotamers), 156.6 & 156.5 (rotamers), 144.1, 141.6 & 141.5 (rotamers), 139.4, 129.9 & 129.8 (rotamers), 127.7 & 127.2 (rotamers), 125.1 & 124.9 (rotamers), 120.1, 119.2, 114.7 & 114.4 (rotamers), 113.6, 67.4 & 66.9 (rotamers), 50.8 & 50.6 (rotamers), 50.1 & 49.9 (rotamers), 47.6 & 47.5 (rotamers), 37.6 & 37.4 (rotamers), 30.5 & 30.2 (rotamers), 28.8 & 28.5 (rotamers), 23.8 & 23.4 (rotamers), 23.2, 14.2, 10.7 & 10.6 (rotamers);
HRMS (ES+): calculated for $C_{30}H_{35}NO_3$ 458.2690 $[M+H]^+$, found 458.2693 $[M+H]^+$;
FT-IR (ATR): ν_{max} /cm^{-1} 3339 (br), 2957, 2928, 1669, 1590, 1478, 1452, 1363, 1233, 1143, 1097.

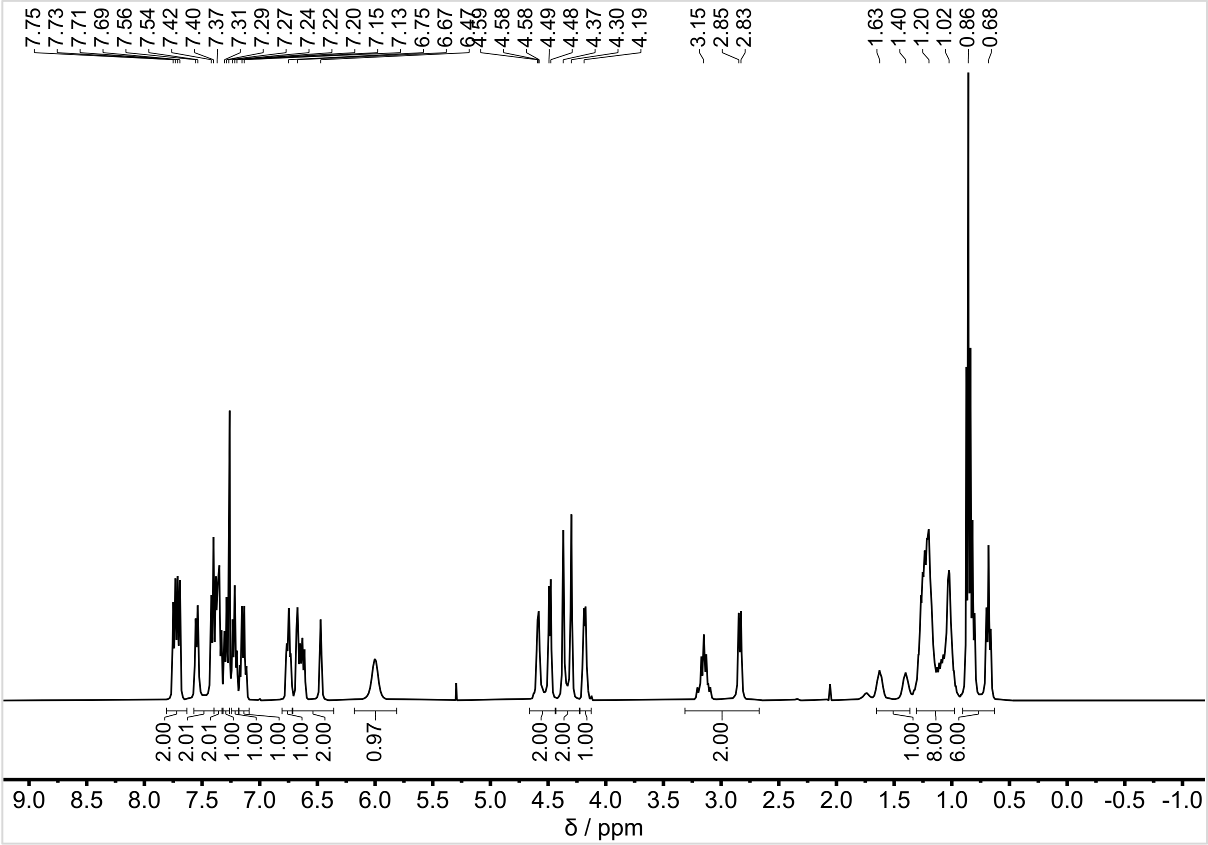

Figure S22: ^{1}H NMR (400 MHz, chloroform-d) spectrum of **13**.

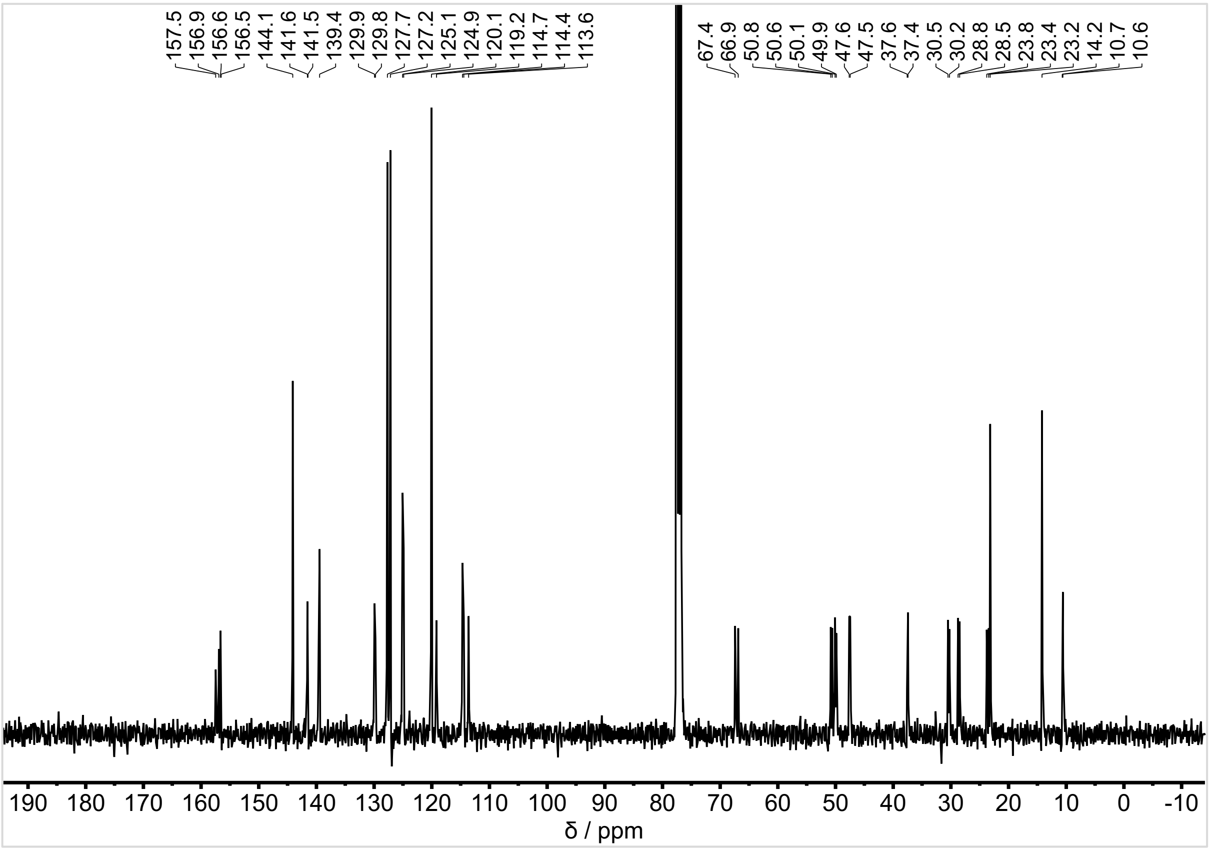

Figure S23: ^{13}C NMR (101 MHz, chloroform-d) spectrum of **13**.

Synthesis of 14

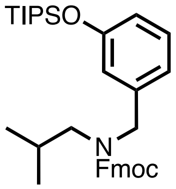

To a solution of **1** (0.720 g, 2.15 mmol) in DCM (8 mL) at 0 °C was added Fmoc chloride (0.610 g, 2.36 mmol), then triethylamine (0.239 g, 0.33 mL, 2.36 mmol) was added dropwise. The reaction was stirred for 2 h at r.t. before the mixture was washed with 1 M K_2CO_3 solution and brine. The organic phase was dried over $MgSO_4$ and then the solvent was removed *in vacuo* to yield the crude product as a yellow oil. The crude was purified by flash chromatography (SiO_2, 0-20% gradient of EtOAc in 40-60 petroleum ether) to yield the product **14** (1.16 g, 2.08 mmol, 97%) as a colourless oil.

The NMR spectra are consistent with the presence of two slowly exchanging rotamers in solution. Where rotamers are distinguishable, the corresponding peaks are listed together.

^{1}H NMR (400 MHz, chloroform-d): δ_H 7.75 & 7.73 (d, J = 7.5 Hz, 2H, rotamers), 7.60 & 7.46 (d, J = 7.2 Hz, 2H, rotamers)7.42-7.33 (m, 2H), 7.32 & 7.23 (t, J = 7.2 Hz, 1H), 7.17-7.12 (m, 1H), 6.81-6.75 (m, 1H), 6.75–6.67 (m, 2H), 4.57 & 4.44 (d, J = 5.5 Hz, 2H, rotamers), 4.45 & 4.43 (s, 2H), 4.25 & 4.20, (t, J = 5.5 Hz, 1H), 3.11 & 2.79 (d, J = 7.4 Hz, 2H, rotamers), 1.98 & 1.69 (non, J = 6.7 Hz, 1H), 1.23 (m, 3H), 1.07 (d, J = 7.2 Hz, 18H), 0.89 & 0.69 (d, J = 6.7 Hz, 6H);
^{13}C NMR (101 MHz, chloroform-d): δ_C 157.1 & 156.7 (rotamers), 156.5 & 156.3 (rotamers), 144.3 & 144.2 (rotamers), 141.6 & 141.4 (rotamers), 139.4 & 139.4 (rotamers), 129.7 & 129.6 (rotamers) 127.7 & 127.2 (rotamers), 125.2 & 124.9 (rotamers), 120.5 & 119.7 (rotamers), 120.0, 119.4 & 118.6 (rotamers), 118.9 & 118.8 (rotamers), 67.6 & 67.1 (rotamers), 54.4 & 53.3 (rotamers), 50.8 & 50.4 (rotamers), 47.6 & 47.5 (rotamers), 27.1, 20.2 & 20.0 (rotamers), 18.0, 12.8;
HRMS (ES+): calculated for $C_{35}H_{47}NO_3Si$ 557.3320 $[M+H]^+$, found 557.3272 $[M+H]^+$;
FT-IR (ATR): ν_{max} /cm^{-1} 2947, 2868, 1694, 1603, 1586, 1486, 1464, 1448, 1421, 1264, 1243, 1149, 1093, 1003, 981, 883, 822, 736.

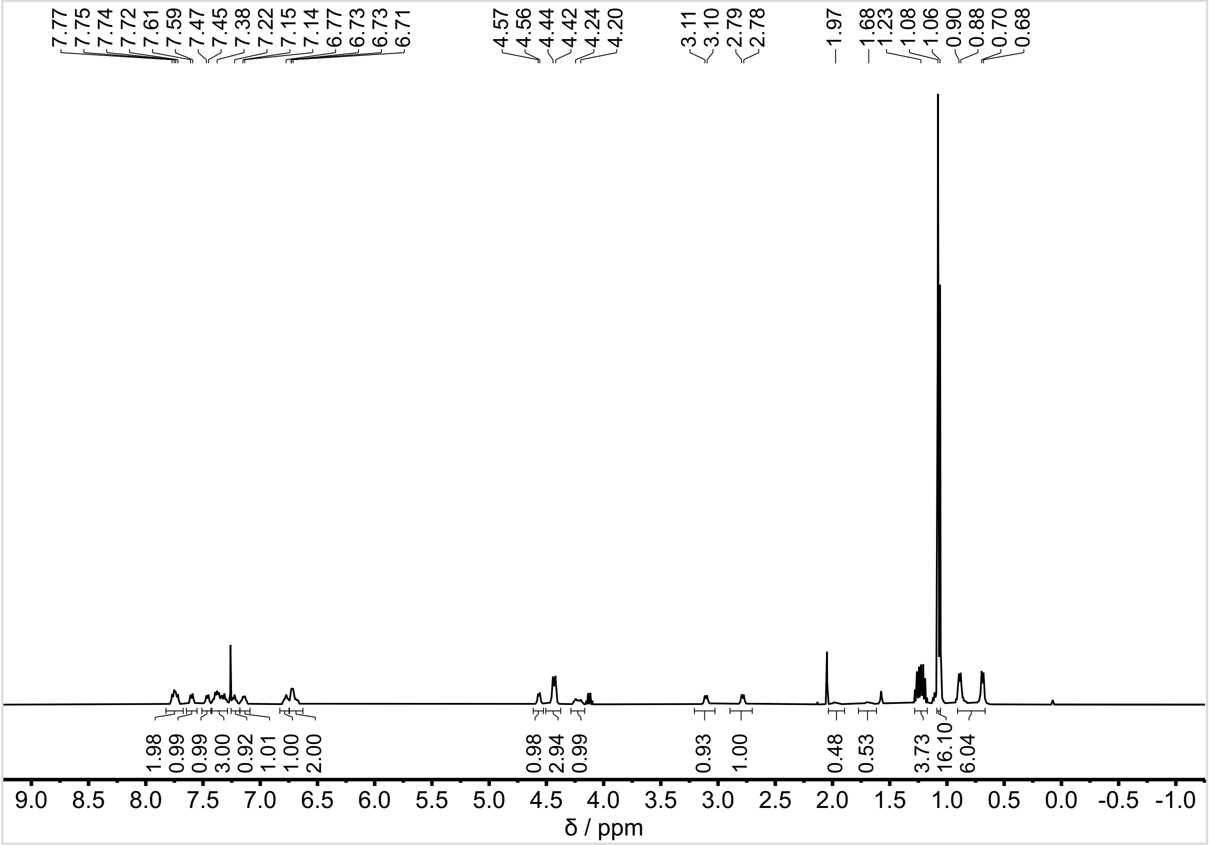

Figure S24: ^{1}H NMR (400 MHz, chloroform-d) spectrum of **14**.

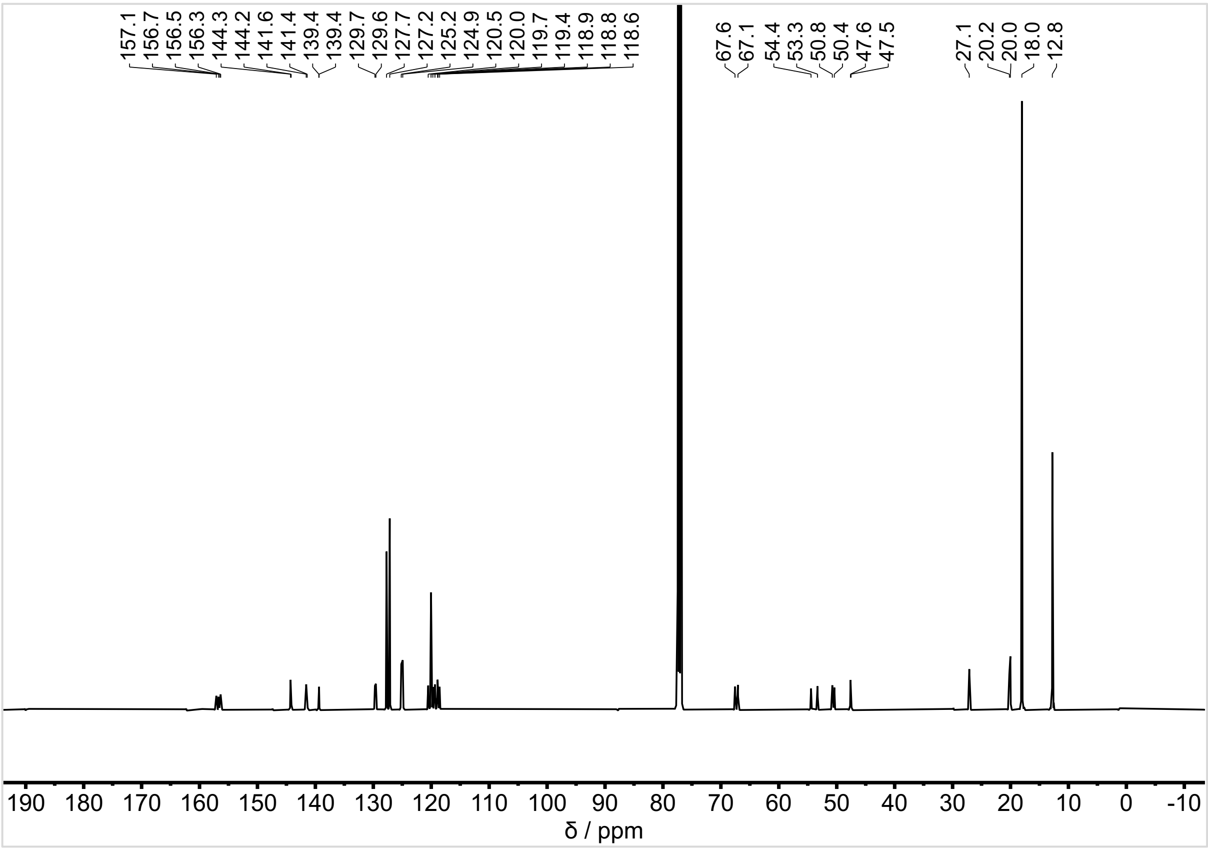

Figure S25: ^{13}C NMR (101 MHz, chloroform-d) spectrum of **14**.

Synthesis of 15

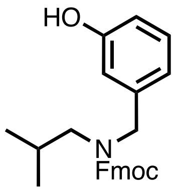

14 (1.00g, 1.79 mmol) was dissolved in THF (15 mL) and the solution was acidified to pH 3 by adding 1 M AcOH. TBAF (3.6 mL, 3.59 mmol, 1 M in THF) was added dropwise and the reaction mixture was stirred at r.t. until complete conversion of the starting material, as monitored by LCMS (approx. 2 h). After completion, the reaction was quenched with 5% aq. soln. HCl and extracted with EtOAc (3x) followed by washing with 5% aq. soln. HCl (3x) and brine. The organic phase was dried over $MgSO_4$ and the solvent was removed *in vacuo* to yield the crude product. The crude was purified by flash chromatography (SiO_2, 0-70% gradient of EtOAc in 40-60 petroleum ether) to yield the pure product **15** (0.592 g, 1.47 mmol, 82%) as a colourless foam.

The NMR spectra are consistent with the presence of two slowly exchanging rotamers in solution. Where rotamers are distinguishable, the corresponding peaks are listed together.

^{1}H NMR (400 MHz, chloroform-d): δ_H 7.75 & 7.70 (d, J = 7.5 Hz, 2H, rotamers), 7.54 & 7.40 (d, J = 7.5 Hz, 2H, rotamers), 7.39-7.32 (m, 2H), 7.29 & 7.21 (t, J = 7.5 Hz, 2H, rotamers), 7.18-7.10 (m, 1H), 6.80-6.72 (m, 1H), 6.68-6.59 (m, 1H), 6.69 & 6.52 (s, 1H, rotamers), 6.43 (br s, 1H), 4.57 & 4.47 (d, J = 5.5, J = 6.2 Hz, 2H, rotamers), 4.38 & 4.33 (s, 2H, rotamers), 4.21-4.14, (m, 1H), 3.08 & 2.73 (d, J = 7.5 Hz, 2H, rotamers), 1.95 & 1.63 (non, J = 6.6 Hz, 1H), 0.84 & 0.65 (d, J = 6.6 Hz, 6H);
^{13}C NMR (101 MHz, chloroform-d): δ_C 157.4 & 157.0 (rotamers), 156.8 & 156.6 (rotamers), 144.1 & 144.0 (rotamers), 141.6 & 141.4 (rotamers), 139.3, 129.9 & 129.7 (rotamers) 127.7 & 127.2 (rotamers), 125.1 & 124.8 (rotamers), 120.5 & 119.7 (rotamers), 120.0, 119.8 & 118.9 (rotamers), 114.7, 114.7 (rotamers), 114.5 & 113.5 (rotamers), 54.4 & 53.3 (rotamers), 67.5 & 67.0 (rotamers), 54.6 & 53.5 (rotamers), 50.9 & 50.3 (rotamers), 47.5 & 47.4 (rotamers), 27.1, 20.1 & 20.0 (rotamers);
HRMS (ES+): calculated for $C_{26}H_{27}NO_3$ 402.2069 $[M+H]^+$, found 402.2057 $[M+H]^+$;
FT-IR (ATR): ν_{max} /cm^{-1} 3333 (br), 2958, 2926, 2871, 1671, 1601, 1590, 1478, 1451, 1426, 1388, 1366, 1249, 1149, 1094, 998, 969, 908, 879, 759, 733, 702, 648.

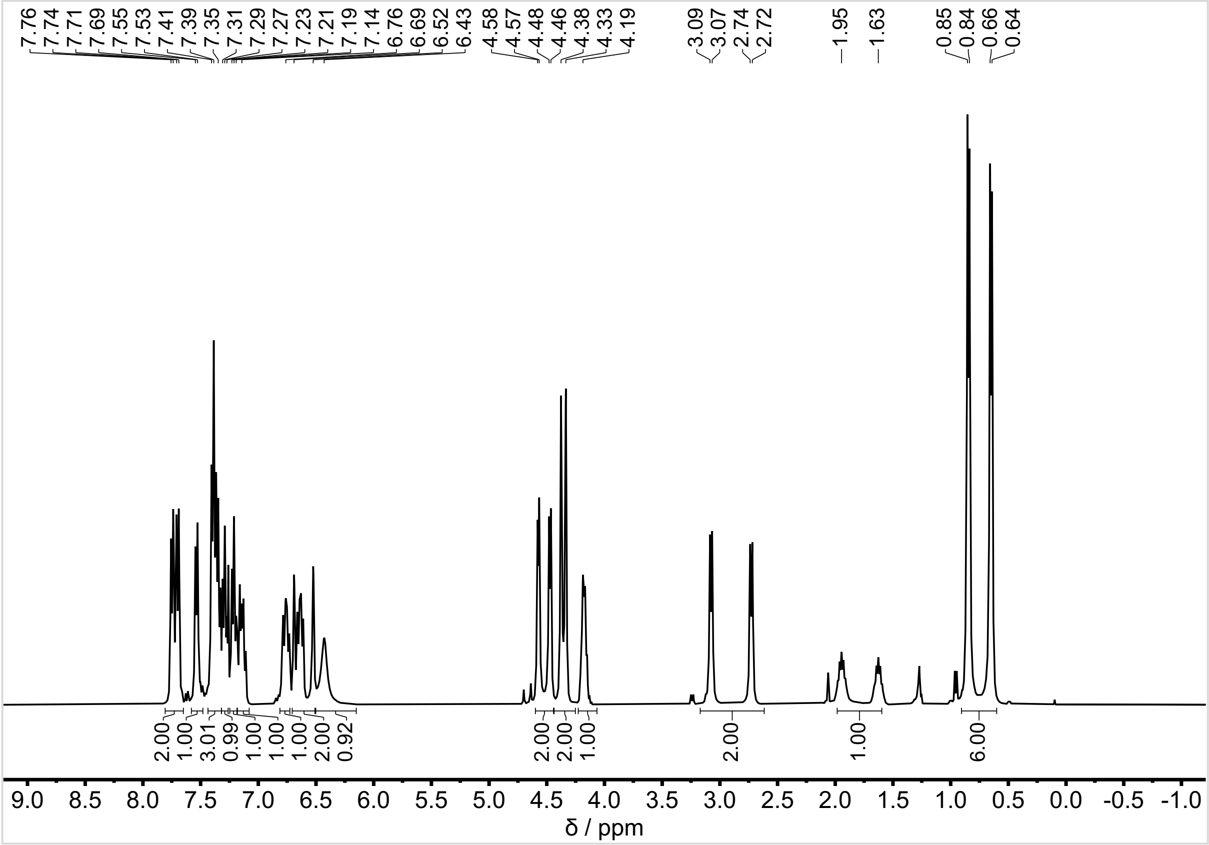

Figure S26: ^{1}H NMR (400 MHz, chloroform-d) spectrum of **15**.

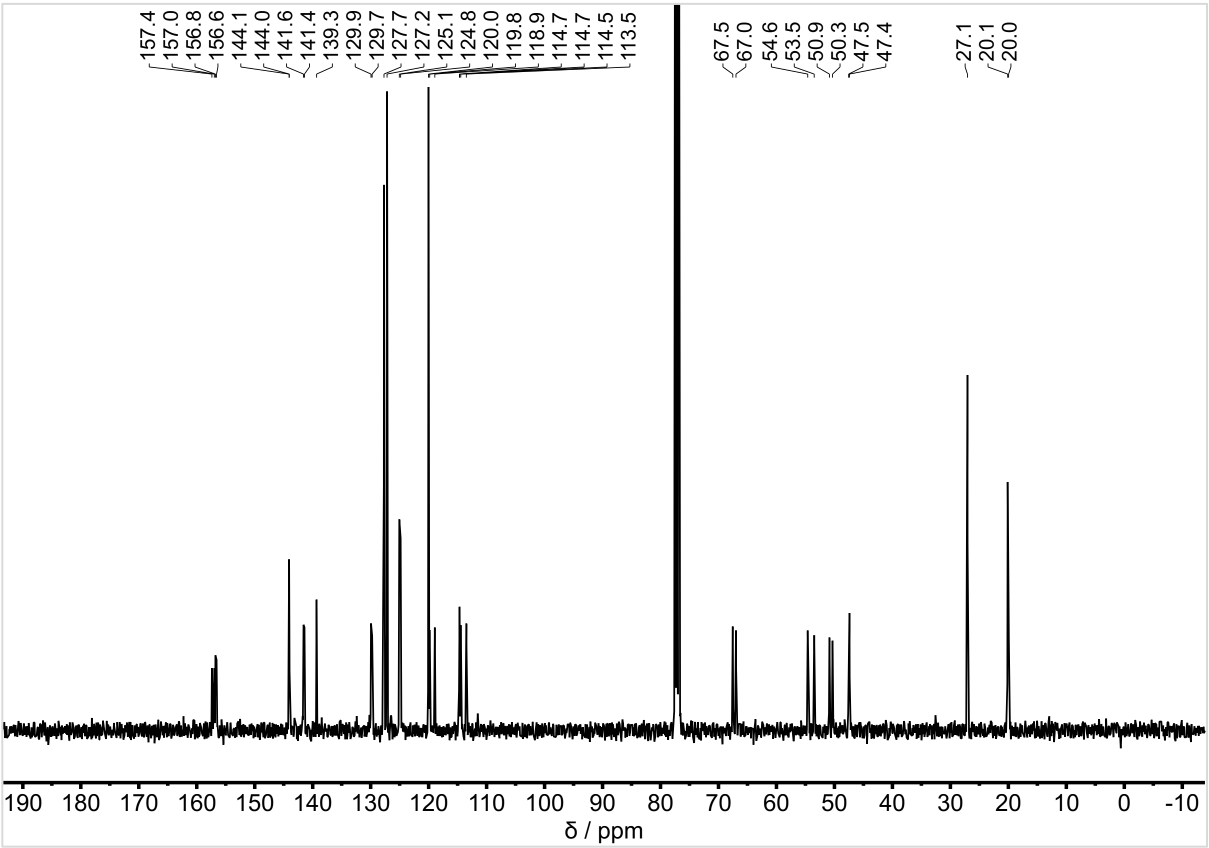

Figure S27: ^{13}C NMR (101 MHz, chloroform-d) spectrum of **15**.

3. Functionalisation of Wang Resin

Synthesis of Resin 1

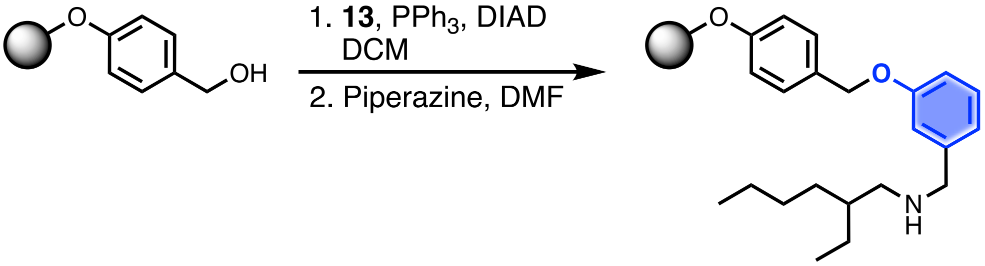

Wang resin (copoly(styrene-1% DVB) 100-200 mesh) (1.00 g, 1.1 mmol based on advertised loading) was swollen in dry DCM for 30 mins before a solution of **15** (0.755 g, 1.65 mmol) and triphenylphosphine (0.433 g, 1.65 mmol) in dry DCM was added to the resin. A solution of diisopropyl azodicarboxylate (0.324 mL, 0.334 g, 1.65 mmol) was diluted 5-fold in dry DCM then added dropwise to the resin. The resin was agitated at r.t. overnight, and then washed alternatingly with DMF (5x) and DCM (5x).

Quantification of resin loading: Functionalised Wang resin was treated with a solution of DBU in DMF (2 mL, 2 vol. %) and agitated for 30 mins. 1 mL of the solution was removed from the resin and diluted with 4 mL acetonitrile. A 1 mL aliquot of the resultant solution was taken and diluted to 12.5 mL with acetonitrile. The absorbance of the DBU-fulvene adduct (λ = 304 nm, ε = 9254 M^{-1} cm^{-1}) was measured to estimate the resin loading (0.36 mmol g^{-1}).

Fmoc deprotection of resin: The functionalised resin was swollen with DCM for 30 mins then washed with DMF (5x) before being treated with a solution of piperidine in DMF (2 x 5mL, 20 vol. %) and agitated for 10 mins. The resin was then washed alternatingly with DMF (5x) and DCM (5x).

4. Protocols for Optimised Automated Solid-Phase Synthesis

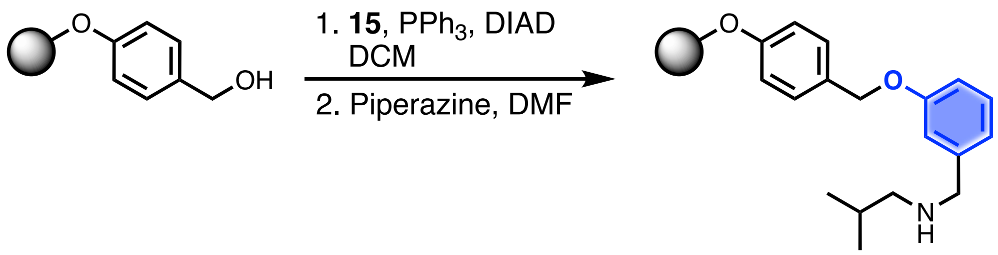

TentaGel Wang Resin (90 µm mesh) (3.00 g, 0.72 mmol based on advertised loading) was swollen in dry DCM for 30 min before a solution of **15** (0.578 g, 1.44 mmol) and triphenylphosphine (0.377g, 1.44 mmol) in dry DCM was added to the resin. A solution of diisopropyl azodicarboxylate (0.284 mL, 0.291 g, 1.44 mmol) was diluted 5-fold in dry DCM then added dropwise to the resin. The resin was agitated at r.t. overnight, and then washed alternatingly with DCM (5x) and DMF (5x) to yield **Resin 2**.

Quantification of resin loading: Functionalised Wang Resin was treated with a solution of DBU in DMF (2 mL, 2 vol. %) and agitated for 30 min. 1 mL of the solution was removed from the resin and diluted with 4 mL acetonitrile. A 1 mL aliquot of the resultant solution was taken and diluted to 12.5 mL with acetonitrile. The absorbance of the DBU-fulvene adduct (λ = 304 nm, ε = 9254 M^{-1} cm^{-1}) was measured to estimate the resin loading (0.14 mmol g^{-1}).

General methods using CEM Liberty Blue Automated Synthesiser: Heated automated Solid-Phase Organic Synthesis (SPS) was performed on a CEM Liberty Blue automated synthesizer on a 50 µmol scale. Solutions of piperazine (0.7 M) in DMF, **2** (0.125 M) in DMF, **7** (0.125 M) in DMF, **10** (0.125 M) in DMF and DIPEA (0.5 M) in DMF were prepared for coupling. General synthetic protocols performed were:

Fmoc deprotection: The loaded Wang resin was agitated in a solution of piperazine in DMF (7 mL, 0.7 M, 2 x 10 min). The deprotection solution was then drained and the resin was washed with DMF (4 x 5 mL).

Coupling cycle: The resin-bound oligomer was first agitated in a solution of **2**, **7** or **10** (0.1 M, 10 eq.) and DIPEA (0.1 M, 10 eq.) in DMF (5 mL) for 10 or 15 mins at 90 °C. The 1st coupling solution was drained, and the resin was washed with DMF (4 x 5 mL). The resin-bound oligomer was then agitated in a solution of piperazine (5 mL, 0.7 M) in DMF for 10 or

15 min at 90 °C. The 2nd coupling solution was drained, and the resin was washed with DMF (4 x 5 mL).

4-Ethynylpiperidine coupling (manual MW-assisted SPS): The resin-bound oligomer was swollen in DMF for 15 min before being agitated in a solution of 4-ethynylpiperidine trifluoroacetate (0.1 M) and DIPEA (1 M) in DMF (5 mL) for 30 mins at 90 °C. The coupling solution was drained, and the resin washed with DMF (4 x 5 mL).

TIPS deprotection: The resin-bound oligomer was swollen in THF for 15 min before being agitated in a solution of TBAF (5 mL, 1 M in THF) at r.t. for 1 h. The solution was drained, and the resin washed with MeOH and THF alternatingly (4 x 5 mL) before being subjected to another deprotection cycle.

Resin Cleavage: The resin was agitated in a mixture of TFA:TIS:DCM (90:5:5 v/v/v) at r.t. for 2 h. The resin was filtered and washed with DCM (5 x 5 mL) and then subjected to another cleavage cycle. The combined filtrates from both cleavage cycles were concentrated under N_2 flow before drying *in vacuo*.

HPLC Purification: Analytical reverse-phase HPLC was performed on an Agilent HP-1100 Series HPLC system composed of a high-pressure binary pump, an autosampler with injector programming capabilities and a diode array detector with a semimicro flow cell (6 mm path length, 5 μL volume). UV/vis absorption was measured at 240 nm and 280 nm (8 nm bandwidth). A Waters XBridge BEH C8 (130 Å, 2.5 μm), 6 x 75 mm column was employed at a flow rate of 0.7 mL/min.

Preparative reverse-phase HPLC was performed on an Agilent HP-1100 Series preparative HPLC system composed of a high-pressure mixing binary pump, dual injector autosampler loops (50 μL/5 mL loops), a variable UV/vis detector (190 nm to 600 nm) and an automatic fraction collector. UV/vis absorption was measured at 240 nm and 280 nm (8 nm bandwidth) and fractions were set to collect automatically based on peak threshold. A Waters XBridge BEH C8 OBD Prep (130 Å, 5 μm), 19 x 250 mm column was employed at a flow rate of 10 mL/min.

Both instruments utilised a mobile phase composed of 95:5 water (HPLC grade):MeCN (HPLC grade) as solvent A, and THF (HPLC grade) as solvent B operating using a linear gradient.

5. Synthesis of Oligomers

Synthesis of zDADAy

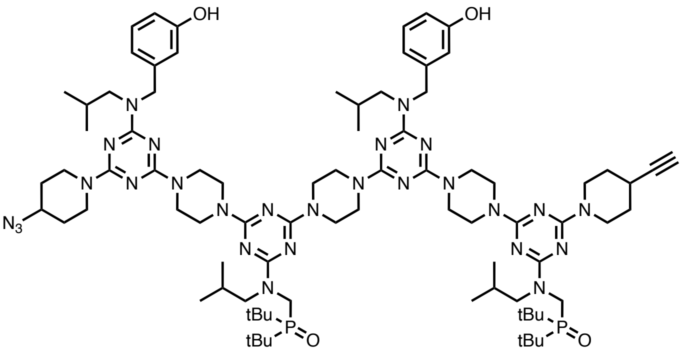

TentaGel Wang resin was loaded with **15** in accordance with the general methods. The loaded resin (50 μmol) was subjected to SPS on a CEM Liberty Blue automated synthesiser, followed by coupling with 4-ethynylpiperidine via manual SPS. TIPS deprotection followed by resin cleavage afforded the crude oligomer. The crude oligomer was purified via preparative HPLC (60% B over 40 mins) to yield the oligomer **zDADAy** (27 mg, 49% based on the initial resin loading) as a white solid.

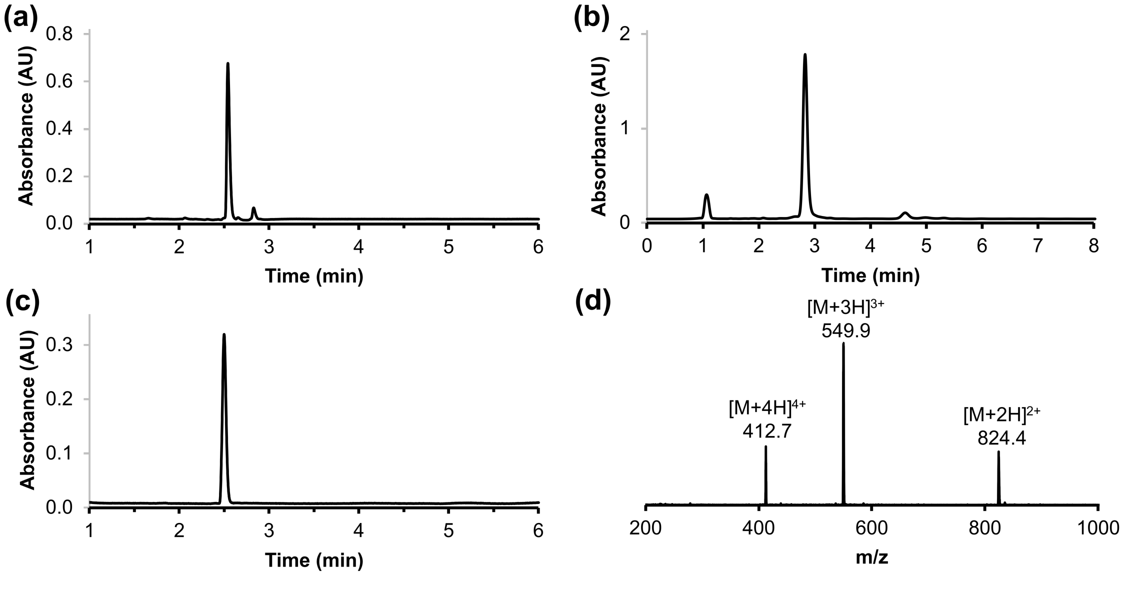

Figure S28: (a) Crude UPLC trace of **zDADAy**. **(b)** Crude analytical HPLC trace of **zDADAy**. **(c)** UPLC trace after purification. **(d)** ESI-MS of **zDADAy**. Calculated Mass: 824.5 $[M+2H]^{2+}$, 550.0 $[M+3H]^{3+}$, 412.8 $[M+4H]^{4+}$; Mass found (ESI^{+}): 824.4 $[M+2H]^{2+}$, 549.9 $[M+3H]^{3+}$, 412.7 $[M+4H]^{4+}$. *UPLC Conditions:* C4 column at 40 °C using a 30-100% gradient of THF/formic acid (0.1%) in water/formic acid (0.1%) over 4 minutes, then 100% THF/formic acid (0.1%) over 2 minutes.

^{1}H NMR (400 MHz, chloroform-d): δ_H 9.17 (br s, 2H), 7.08 (t, *J* = 7.8 Hz, 2H), 6.88-6.74 (m, 4H), 6.74-6.67 (m, 2H), 4.83-4.79 (m, 4H), 4.52-4.26 (m, 6H), 4.17-4.11 (m, 2H), 3.95-3.53 (m, 29H), 3.51-3.39 (m, 2H), 3.37-3.17 (m, 6H), 2.61 (s, 1H), 2.25-2.15 (m, 2H), 2.13-2.05 (m, 3H), 1.98-1.73 (m, 4H), 1.71-1.46 (m, 4H), 1.30 (d, $^{3}J_{PH}$ = 12.6 Hz, 36H), 0.91-0.84 (m, 24H);

^{31}P NMR (162 MHz, chloroform-d): δ_P 61.32;

HRMS (ES+): calculated for $C_{84}H_{133}N_{27}O_4P_2$ 1648.0610 $[M+H]^+$, found 1648.0614 $[M+H]^+$.

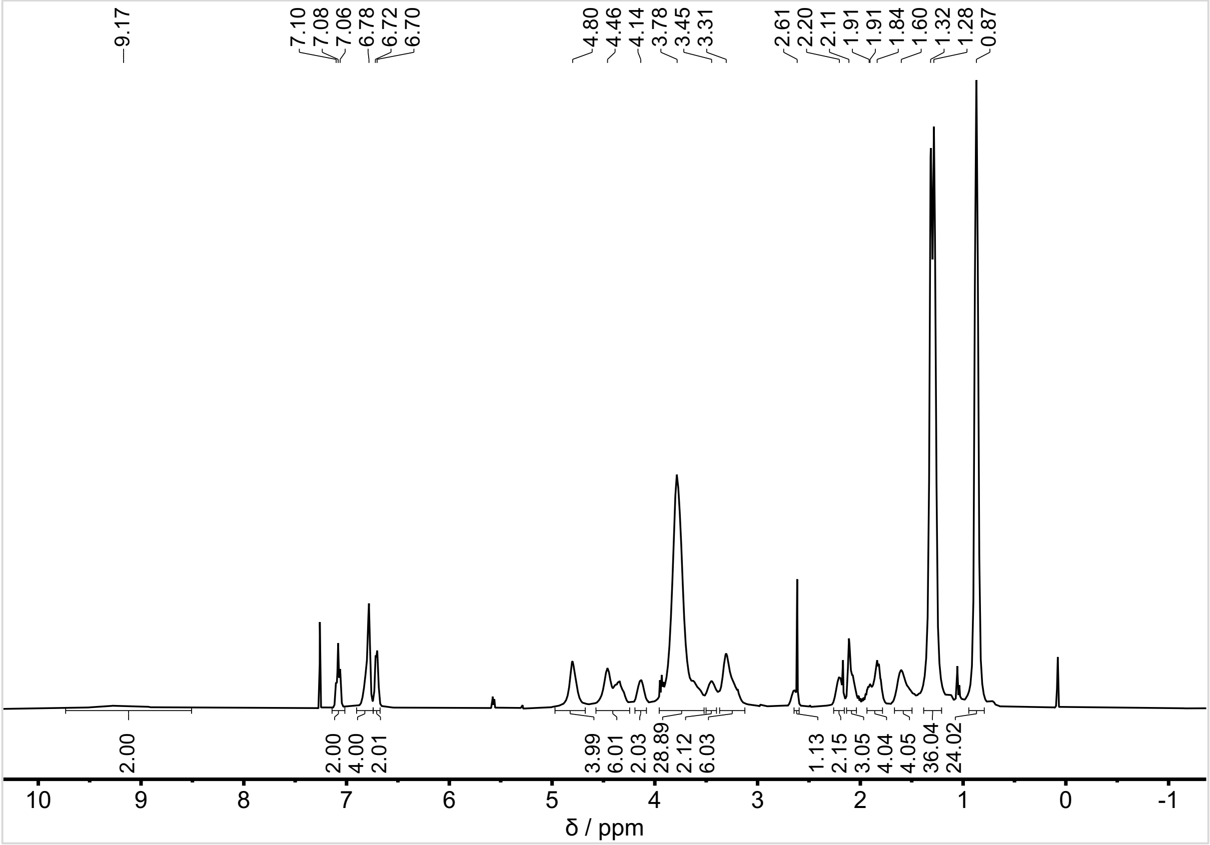

Figure S29: ^{1}H NMR (400 MHz, chloroform-d) spectrum of **zDADAy**.

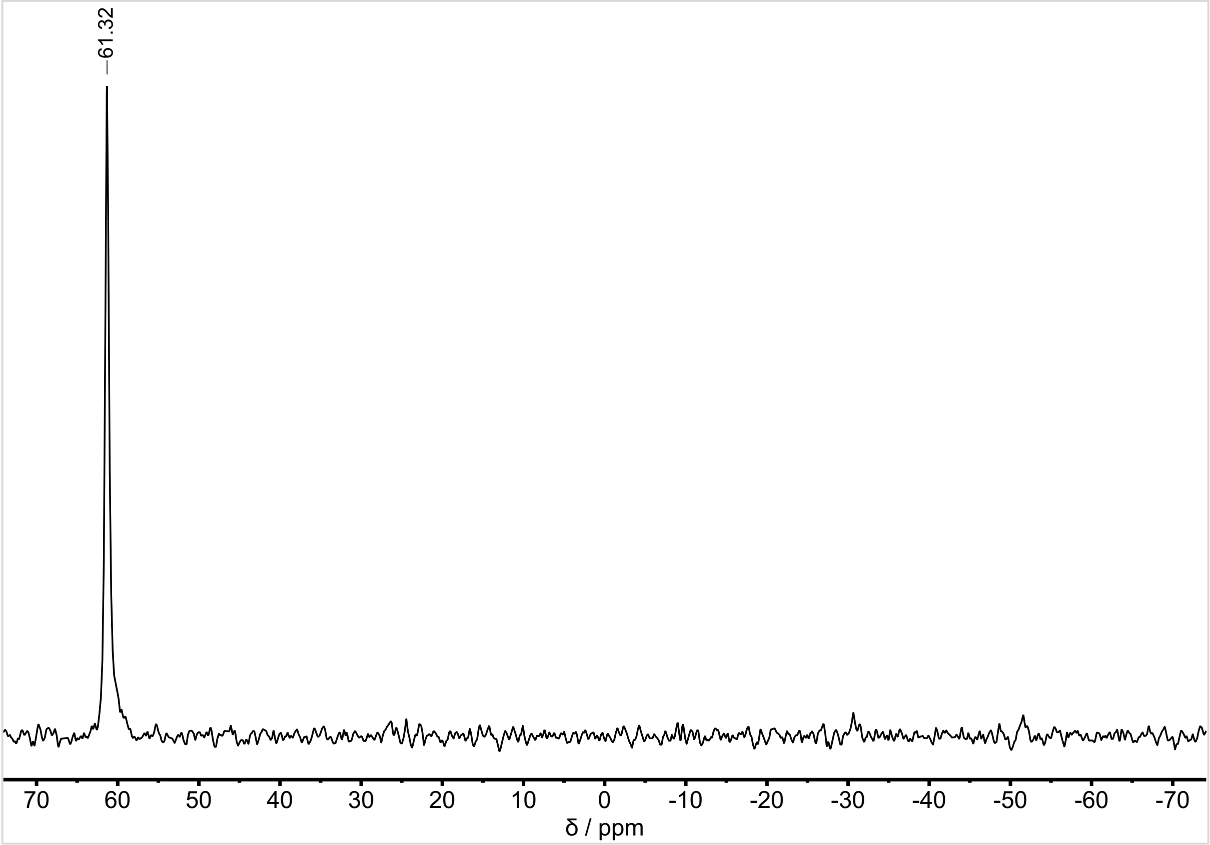

Figure S30: ^{31}P NMR (162 MHz, chloroform-d) spectrum of **zDADAy**.

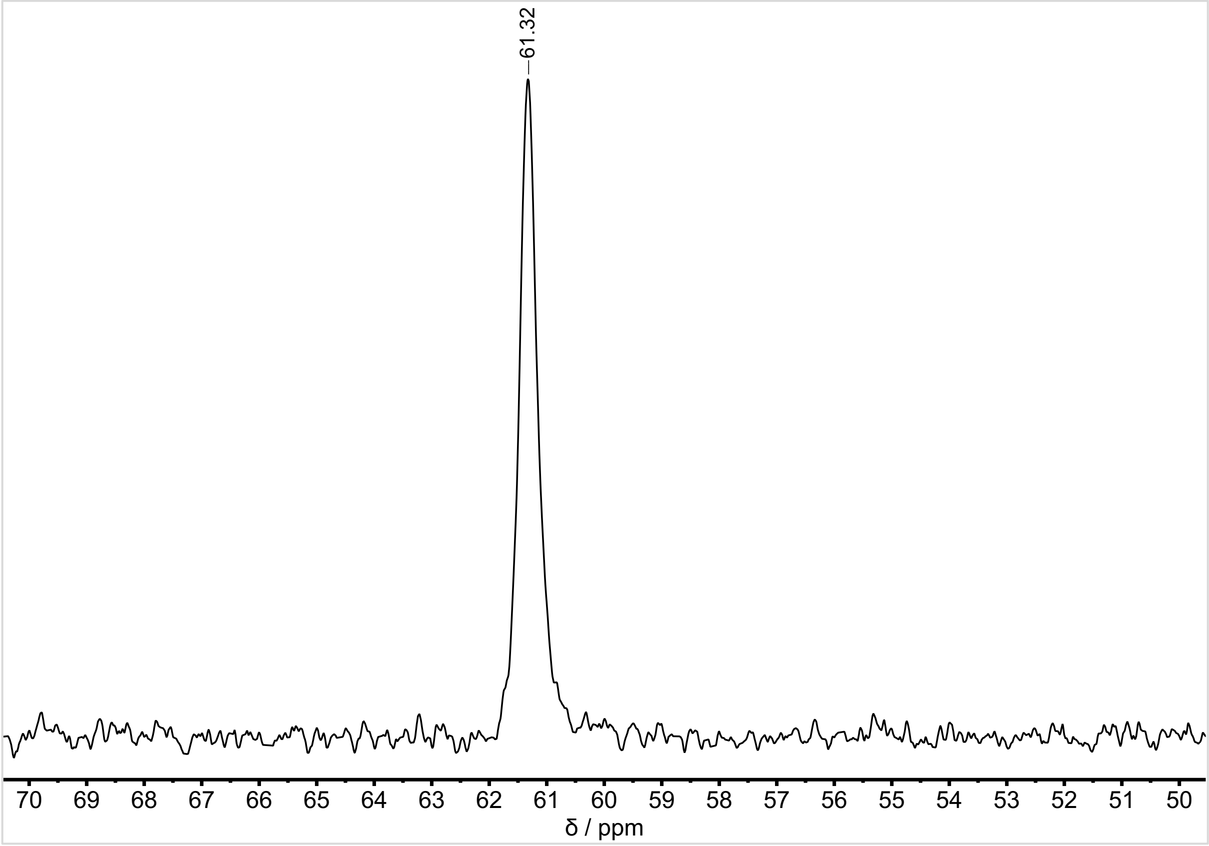

Figure S31: ^{31}P NMR (162 MHz, chloroform-d) spectrum of **zDADAy**.

Synthesis of zDDDDDDDDDDDDy

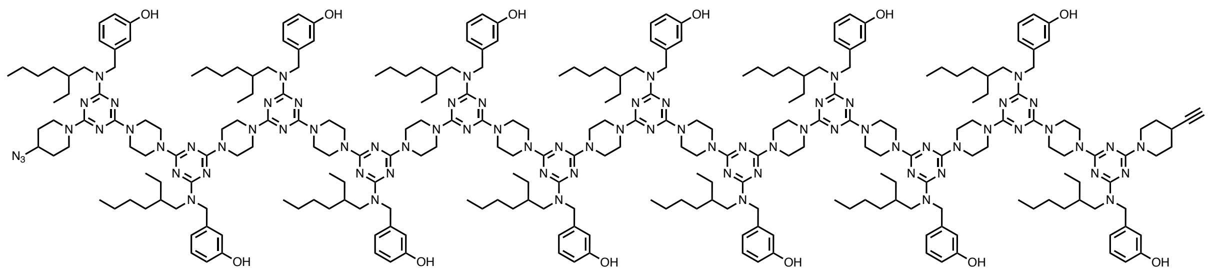

TentaGel Wang resin was loaded with **13** in accordance with the general methods. The loaded resin (50 μmol) was subjected to SPS on a CEM Liberty Blue automated synthesiser, followed by coupling with 4-ethynylpiperidine via manual SPS. TIPS deprotection followed by resin cleavage afforded the crude oligomer. The crude oligomer was purified via preparative HPLC (72.5% B over 38 mins, then 72.5 to 85% B over 4 mins) to yield the oligomer **zDDDDDDDDDDDDy** (68 mg, 28% based on the initial resin loading) as a white solid.

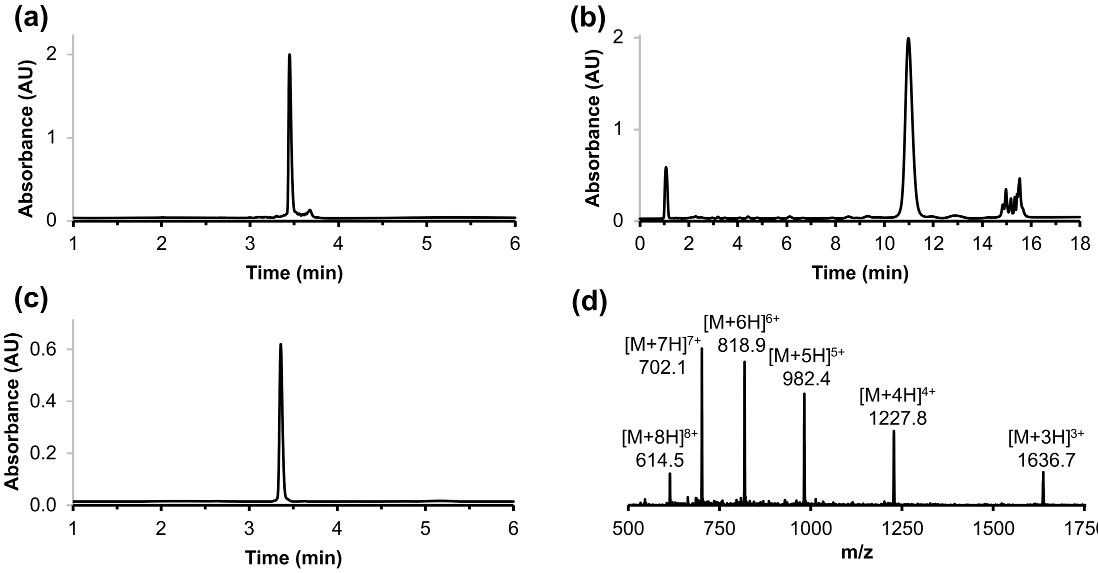

Figure S32: (a) Crude UPLC trace of **zDDDDDDDDDDDDy**. **(b)** Crude analytical HPLC trace. **(c)** UPLC trace after purification. **(d)** ESI-MS of **zDDDDDDDDDDDDy**. Calculated Mass: 1636.8 [M+3H]$^{3+}$, 1227.8 [M+4H]$^{4+}$, 982.5 [M+5H]$^{5+}$, 818.9 [M+6H]$^{6+}$, 702.0 [M+7H]$^{7+}$, 614.4 [M+8H]$^{8+}$; Mass found (ESI^{+}): 1636.7 [M+3H]$^{3+}$, 1227.8 [M+4H]$^{4+}$, 982.4 [M+5H]$^{5+}$, 818.9 [M+6H]$^{6+}$, 702.1 [M+7H]$^{7+}$, 614.5 [M+8H]$^{8+}$. *UPLC Conditions:* C4 column at 40 °C using a 30-100% gradient of THF/formic acid (0.1%) in water/formic acid (0.1%) over 4 minutes, then 100% THF/formic acid (0.1%) over 2 minutes.

^{1}H NMR (400 MHz, DMSO-d$_6$): δ_H 9.27-9.09 (phenol OH, 12H), 7.22-6.44 (aryl CH, 48H), 5.76-2.66 (NCH_2, 145H), 1.84-1.61 (2-ethylhexyl CH, 12H), 1.59-0.93 (CH_2 & C≡CH, 106H), 0.90-0.56 (2-ethylhexyl CH_3, 72H);

HRMS (ES+): calculated for $C_{272}H_{395}N_{75}O_{12}$ 2454.6418 $[M+2H]^{2+}$, found 2454.6449 $[M+2H]^{2+}$.

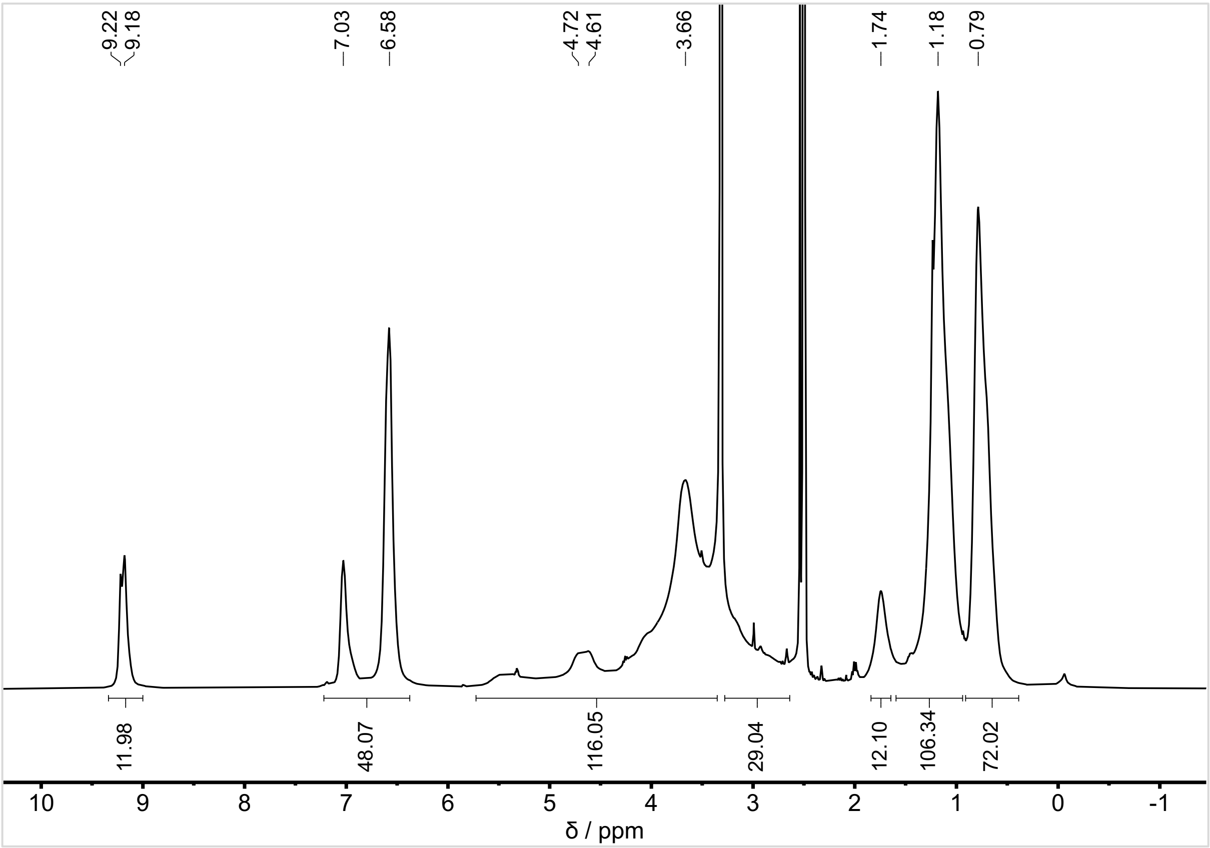

Figure S33: ^{1}H NMR (400 MHz, DMSO-d$_6$) spectrum of **zDDDDDDDDDDDDy**.

Synthesis of zDAAAAAAAAAAAAy

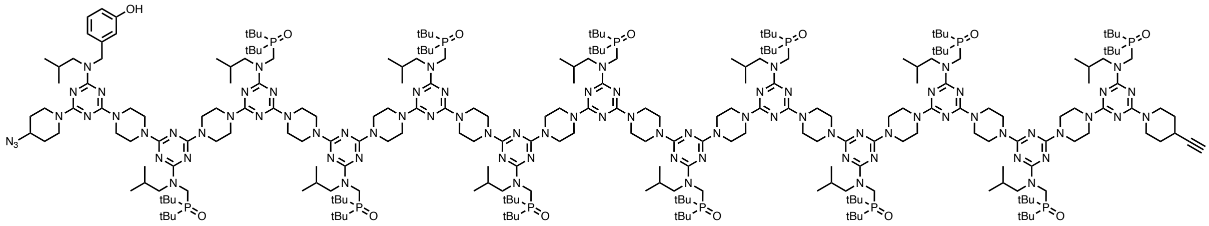

TentaGel Wang resin was loaded with **15** in accordance with the general methods. The loaded resin (50 μmol) was subjected to SPS on a CEM Liberty Blue automated synthesiser, followed by coupling with 4-ethynylpiperidine via manual SPS. TIPS deprotection followed by resin cleavage afforded the crude oligomer. The crude oligomer was purified via preparative HPLC (47.5% B over 40 mins) to yield the oligomer **zDAAAAAAAAAAAAy** (96 mg, 36% based on the initial resin loading) as a white solid.

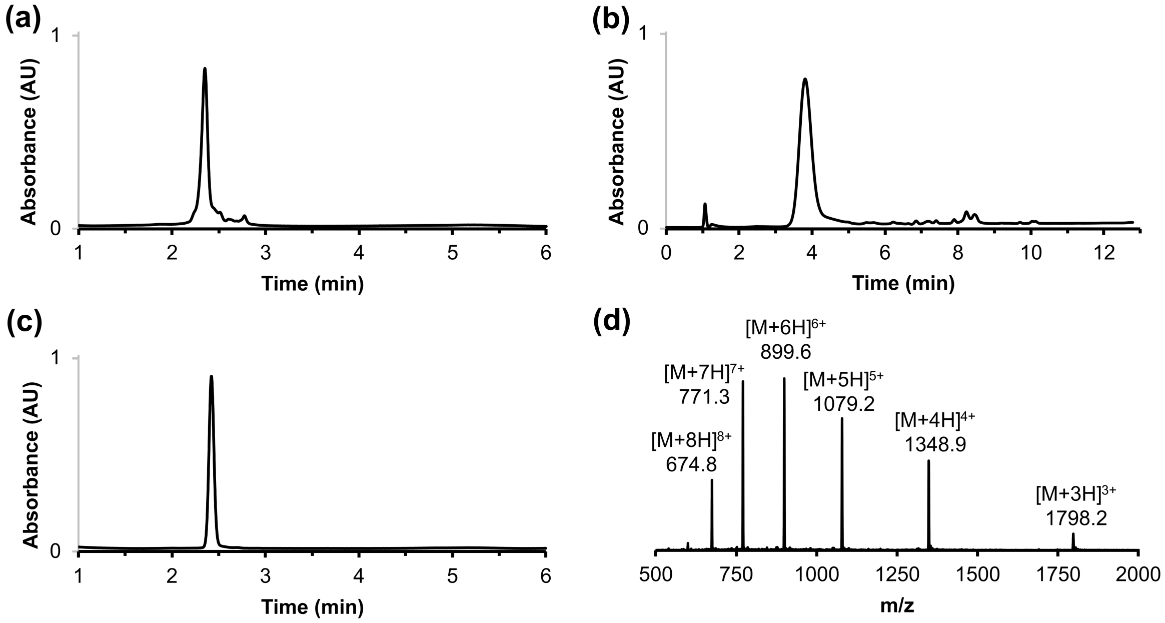

Figure S34: (a) Crude UPLC trace of **zDAAAAAAAAAAAAy**. **(b)** Crude analytical HPLC trace. **(c)** UPLC trace after purification. **(d)** ESI-MS of **zDAAAAAAAAAAAAy**. Calculated Mass: 1798.2 $[M+3H]^{3+}$, 1348.9 $[M+4H]^{4+}$, 1079.3 $[M+5H]^{5+}$, 899.6 $[M+6H]^{6+}$, 771.2 $[M+7H]^{7+}$, 674.8 $[M+8H]^{8+}$; Mass found (ESI$^+$): 1798.2 $[M+3H]^{3+}$, 1348.9 $[M+4H]^{4+}$, 1079.2 $[M+5H]^{5+}$, 899.6 $[M+6H]^{6+}$, 771.3 $[M+7H]^{7+}$, 674.8 $[M+8H]^{8+}$. *UPLC Conditions:* C4 column at 40 °C using a 30-100% gradient of THF/formic acid (0.1%) in water/formic acid (0.1%) over 4 minutes, then 100% THF/formic acid (0.1%) over 2 minutes.

^{1}H NMR (400 MHz, chloroform-d): δ_H 7.14-6.69 (aryl CH, 4H) 4.84-3.21 (NCH_2, 157H), 2.66 ((HC≡C)C<u>H</u>, 1H), 2.29-1.55 (CH_2, *i*-Bu CH & C≡CH, 21H), 1.39-1.22 (*t*-Bu CH_3, 216H) 0.93-0.81 (*i*-Bu CH_3, 78H);

^{31}P NMR (162 MHz, chloroform-d): δ_P 61.44, 58.76;

HRMS (ES+): calculated for $C_{266}H_{479}N_{81}O_{13}P_{12}$ 2696.8196 $[M+2H]^{2+}$, found 2696.8337 $[M+2H]^{2+}$.

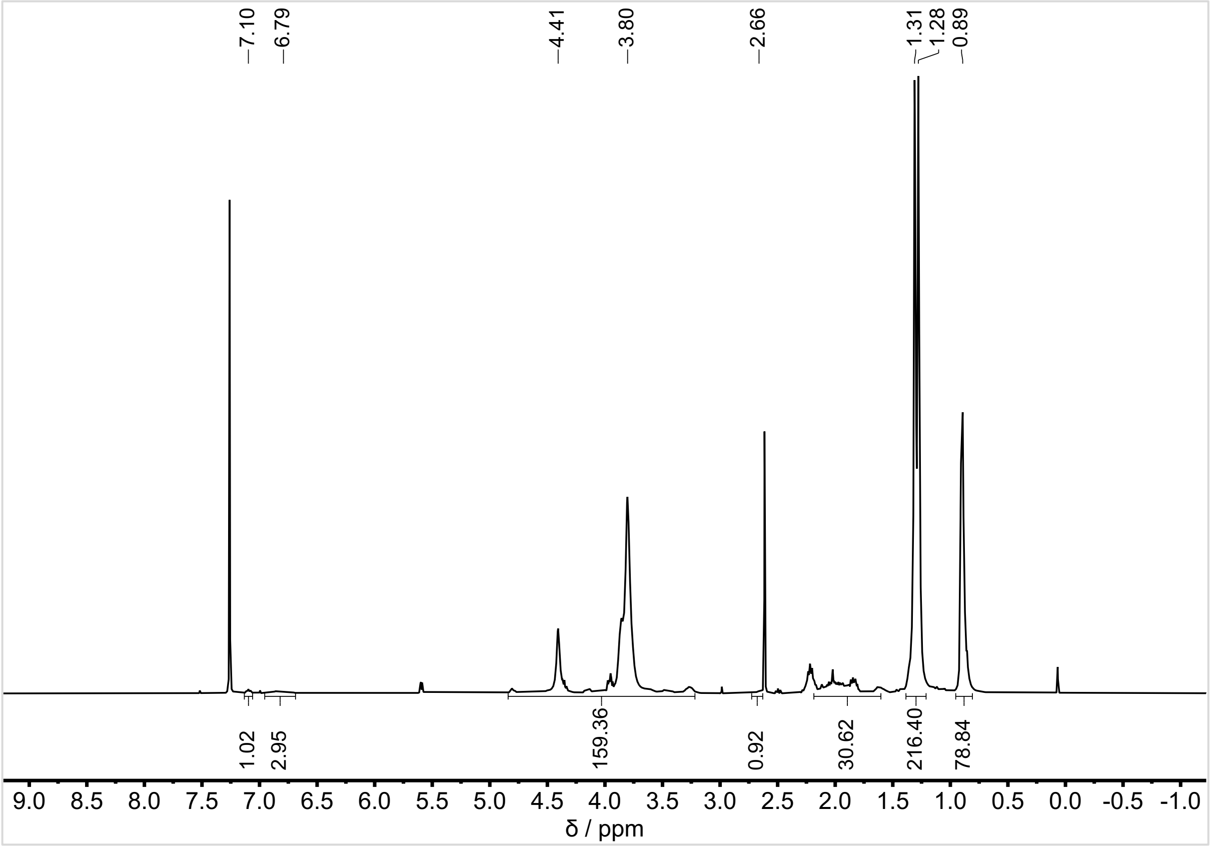

Figure S35: ^{1}H NMR (400 MHz, chloroform-d) spectrum of **zDAAAAAAAAAAAAy**.

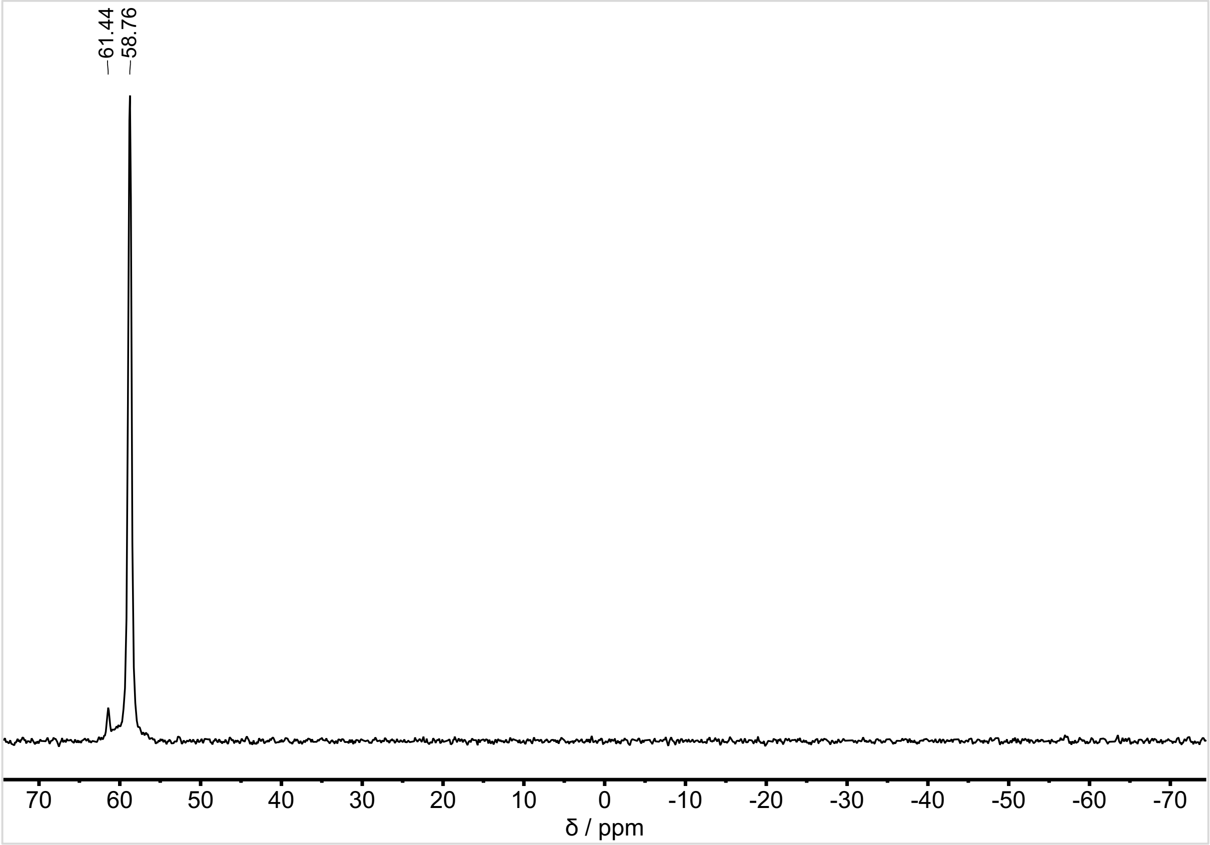

Figure S36: ^{31}P NMR (162 MHz, chloroform-d) spectrum of **zDAAAAAAAAAAAAy**.

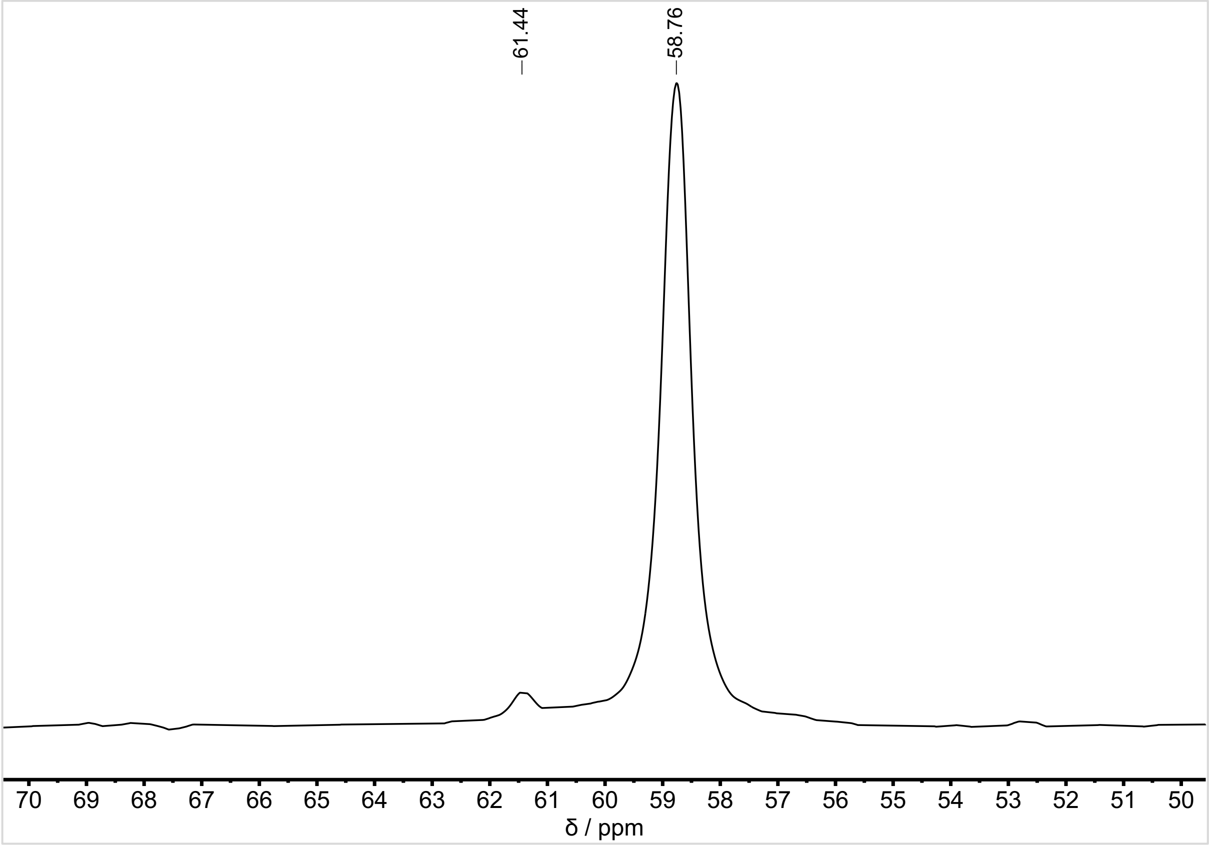

Figure S37: ^{31}P NMR (162 MHz, chloroform-d) spectrum of **zDAAAAAAAAAAAAy**.

Synthesis of 42-mer

zDDDDDDADDDDDADDDADDADAAAAAADAAAAADAAADAADAy

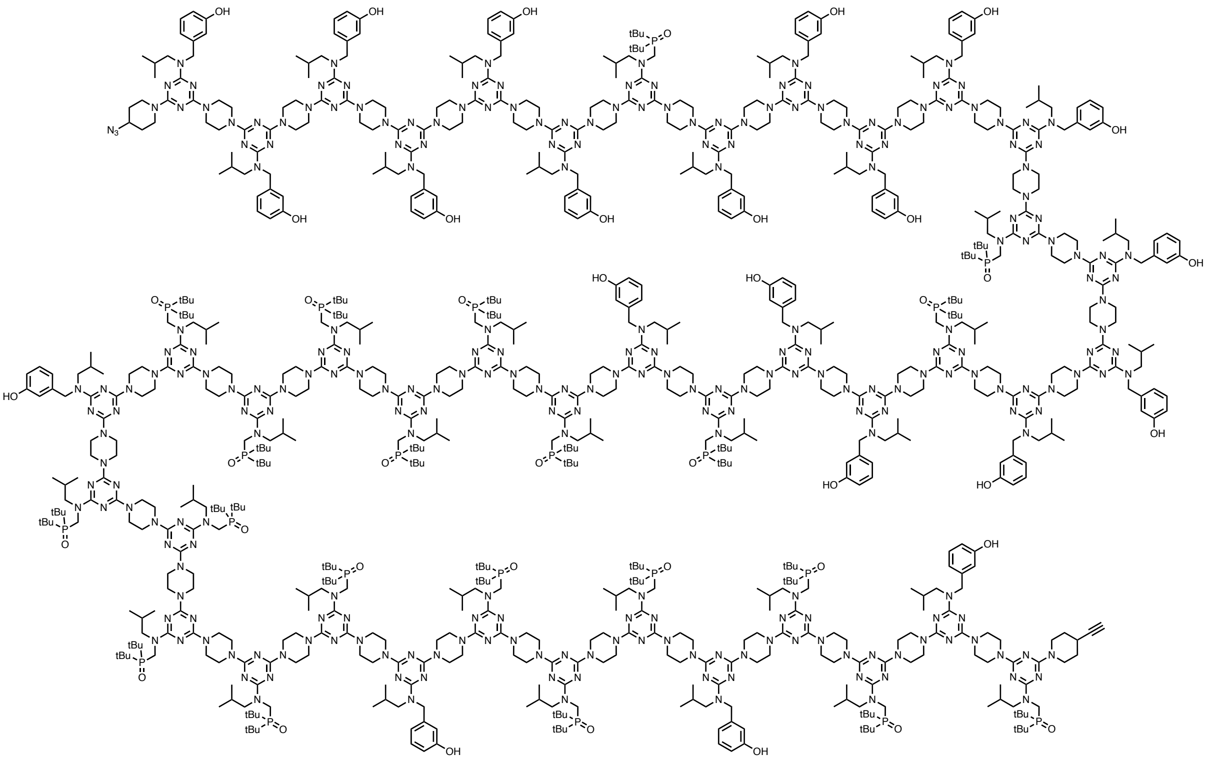

TentaGel Wang resin was loaded with **15** in accordance with the general methods. The loaded resin (50 μmol) was subjected to SPS on a CEM Liberty Blue automated synthesiser, followed by coupling with 4-ethynylpiperidine via manual SPS. TIPS deprotection followed by resin cleavage afforded the crude oligomer.

The crude oligomer was purified via preparative HPLC (57.5% to 62.5% B over 40 mins) to yield **zDDDDDDADDDDDADDDADDADAAAAAADAAAAADAAADAADAy** (155 mg, 22% based on the initial resin loading) as a white solid.

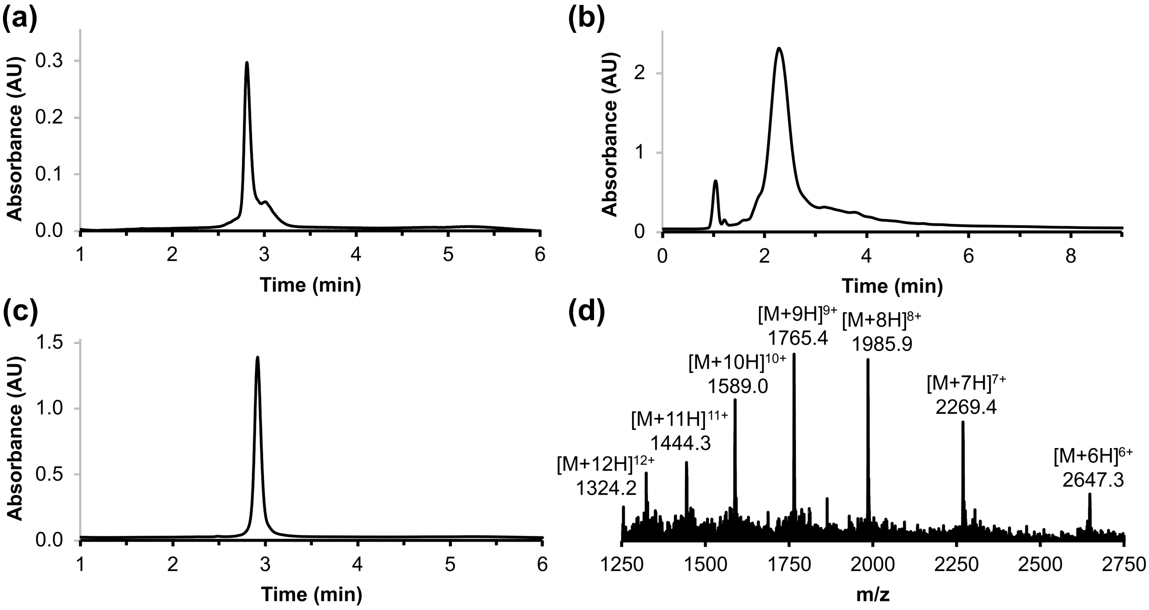

Figure S38: **(a)** Crude UPLC trace of 42-mer **zDDDDDDADDDDDADDDADDADAAAAAADAAAAADAAADAADAy**. **(b)** Crude analytical HPLC trace. **(c)** UPLC trace after purification. **(d)** ESI-MS of 42-mer **zDDDDDDADDDDDADDDADDADAAAAAADAAAAADAAADAADAy**. Calculated Mass: 2647.2 $[M+6H]^{6+}$, 2269.3 $[M+7H]^{7+}$, 1985.9 $[M+8H]^{8+}$, 1765.2 $[M+9H]^{9+}$, 1588.8 $[M+10H]^{10+}$, 1444.3 $[M+11H]^{11+}$, 1324.1 $[M+12H]^{12+}$; Mass found (ESI^{+}): 2647.2 $[M+6H]^{6+}$, 2269.4 $[M+7H]^{7+}$, 1985.9 $[M+8H]^{8+}$, 1765.4 $[M+9H]^{9+}$, 1589.0 $[M+10H]^{10+}$, 1444.3 $[M+11H]^{11+}$, 1324.2 $[M+12H]^{12+}$. *UPLC Conditions:* C4 column at 40 °C using a 30-100% gradient of THF/formic acid (0.1%) in water/formic acid (0.1%) over 4 minutes, then 100% THF/formic acid (0.1%) over 2 minutes.

^{1}H NMR (400 MHz, MeOD:THF-d_8, 1:9 v/v): δ_H 7.14-6.38 (aryl CH, 84H) 4.85-3.17 (NCH_2 region), 2.29-1.55 (CH_2, *i*-Bu CH region), 1.34-1.22 (*t*-Bu CH_3, 378H) 0.97-0.80 (*i*-Bu CH_3, 252H).

Some regions of the ^{1}H NMR spectrum could not be easily integrated due to overlapping THF and MeOH signals.

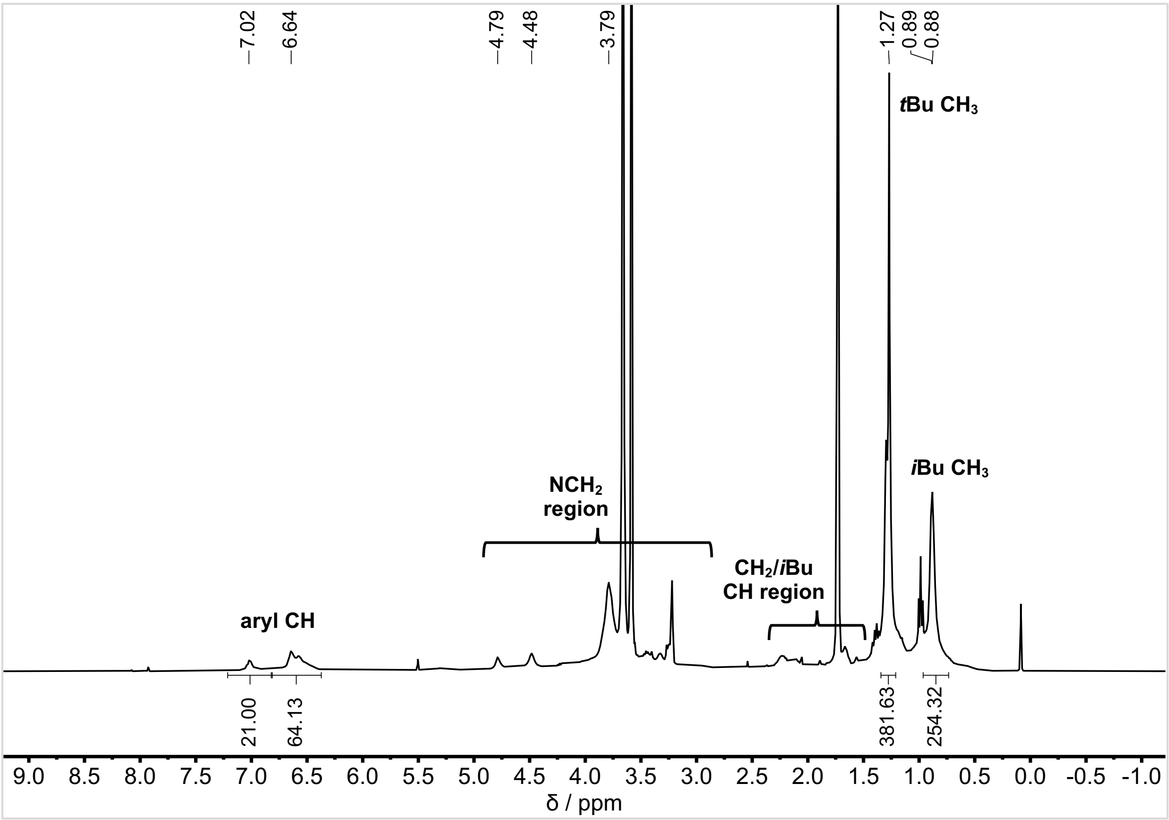

Figure S39: ^{1}H NMR (400 MHz, THF-d_8/methanol-d) spectrum of the 42-mer **zDDDDDDADDDDDADDDADDADAAAAAADAAAAADAAADAADAy**.
